# Dynamic contrast-enhanced ultrasound predicts microsphere biodistribution in real-time during transarterial radioembolization

**DOI:** 10.1038/s43856-026-01760-x

**Published:** 2026-06-30

**Authors:** J. L. van der Hoek, T. J. Snoeijink, A. Visser, K. D. E. de Bree, M. E. Krommendijk, J. G. M. Greve, H. R. Liefers, W. M. Brink, M. Versluis, J. Arens, S. Manohar, E. Groot Jebbink

**Affiliations:** 1 Multi-Modality Medical Imaging Group, TechMed Centre, Faculty of Science and Technology, University of Twente, Enschede, The Netherlands; 2 Department of Medical Imaging, Radboud University Medical Centre, Nijmegen, The Netherlands; 3 TechMed Simulation Centre, University of Twente, Enschede, The Netherlands; 4 Physics of Fluids Group, TechMed Centre, Faculty of Science and Technology, University of Twente, Enschede, The Netherlands; 5 Engineering Organ Support Technologies Group, Faculty of Engineering Technology, University of Twente, Enschede, The Netherlands

**Keywords:** Targeted therapies, Radiotherapy, Blood flow, Ultrasound, Drug delivery

## Abstract

**Background:**

Transarterial radioembolization (TARE) treatment efficacy critically depends on the microsphere biodistribution in the liver. Current treatment planning protocols rely on a pre-treatment scout procedure to evaluate this biodistribution for a selected catheter configuration. However, replication of the catheter configuration during treatment is challenging and often results in suboptimal tumor targeting. Here we present and experimentally validate a concept to improve tumor targeting using dynamic contrast-enhanced ultrasound (DCE-US) imaging to predict the microsphere biodistribution in real-time, using ultrasound contrast microbubbles as microsphere precursors.

**Methods:**

DCE-US-guided TARE was performed in three ex vivo perfused porcine livers using a customized normothermic machine-perfusion platform. In each liver, one lobe was selected for targeting, and a 9L4 ultrasound transducer was fixed to image this location. A microcatheter was inserted in the hepatic artery under fluoroscopic guidance. Several catheter positions were analyzed in real-time using ultrasound contrast microbubbles and time-intensity curves (TICs). The catheter position was optimized based on peak TIC intensity, which was retained for subsequent injection of non-irradiated ^165^Ho-microspheres. The resulting microsphere biodistribution was evaluated by R2^*^ mapping in MRI.

**Results:**

Here we show that DCE-US can be used to identify feeding arteries, enabling optimization of the catheter placement to maximize peak intensity within the target lobe. MRI confirms the relative microsphere biodistribution as predicted by DCE-US in two quantifiable cases (Case 2: 99.2% [MRI] vs. 98% [DCE-US]; Case 3: 70.2% [MRI] vs. 80.9% [DCE-US]).

**Conclusions:**

DCE-US enables accurate prediction of microsphere biodistribution, providing real-time feedback for immediate intra-operative optimization of the catheter position during TARE.

## Plain language summary

Transarterial radioembolization is a treatment for liver tumors in which small radioactive particles are injected into an artery that feeds the tumor. For the treatment to work well, these particles must reach the tumors while avoiding healthy liver tissue. However, doctors currently have limited ability to see where the particles will go during the procedure. In this study, we investigated whether imaging technologies using microscopic bubbles could help predict the distribution of these particles in real time. Using pig livers, we show how these bubbles can be used to test how well different injection strategies can selectively target specific regions of the liver. The predictions closely matched the final particle distribution. This approach could help physicians adjust the injection strategy during treatment to better target tumors and potentially improve treatment outcomes.

## Abstract (web summary)

van der Hoek et al. demonstrate a real-time prediction method for microsphere biodistribution in transarterial radioembolization using dynamic contrast-enhanced ultrasound in ex vivo perfused porcine livers. The approach predicts microsphere distributions and can be used to iteratively optimize the catheter position.

## Introduction

Transarterial radioembolisation (TARE) is an established locoregional treatment for unresectable and chemorefractory liver cancer^1^. The procedure involves the transcatheter injection of microspheres, which contain the radioactive isotope yttrium-90 (^90^Y) or holmium-166 (^166^Ho), into the hepatic artery or its branches^2–4^. Due to their size (typical average diameter of 25–32 µm^5^), these microspheres lodge in the hepatic arterioles, interrupting the local blood flow while simultaneously delivering targeted radiation over time.

TARE treatment efficacy is directly related to the realized microsphere biodistribution. After microsphere injection, a high tumor-absorbed dose with a low non-tumor-absorbed dose (high T/N ratio) is associated with a higher median overall survival. A sub-analysis of the SARAH trial demonstrated that patients receiving a median tumor-absorbed dose of ≥ 100 Gy had a significantly longer median overall survival (14.1 months) compared to those receiving <100 Gy (6.1 months)^6^, while a mean non-tumor-absorbed dose of ≤ 40 Gy is recommended for safety^7^. Although microspheres exhibit a natural tendency to accumulate in tumors due to their enhanced arterial vascularity^8^, the microsphere biodistribution is significantly influenced by the catheter configuration used, including injection flow velocity^9–11^, injection flow profile^12,13^ and catheter tip location^14–16^. Consequently, the choice of catheter configuration for TARE has a direct impact on treatment outcome.

Current treatment planning strategies for TARE involve injecting scout particles using the candidate catheter configuration, typically one to two weeks before the treatment procedure. In this scout procedure, technetium-99m macroaggregated albumin (^99m^Tc-MAA) beads (95% between 10 – 100 µm diameter^17^) are commonly used as scout particles, which can be visualized by single-photon emission computed tomography/computed tomography (SPECT/CT) to assess their biodistribution^18,19^. Alternatively, it was shown that a small amount of ^166^Ho-microspheres can also be used as a scout dose for ^166^Ho-TARE^20^. The use of identical particles in both the scout and therapeutic procedures, combined with magnetic resonance imaging (MRI) dosimetry enabled by the paramagnetic properties of ^166^Ho-microspheres^21^, was shown to improve the prediction of intrahepatic microsphere biodistribution^22^.

The predictive value of the scout procedure is compromised, however, by the long time interval between the scout and therapeutic TARE procedures (typically 1–2 weeks). This increases the likelihood of changes in hemodynamics at the time of treatment (e.g., alterations in blood pressure or hepatic arterial flow distribution due to tumor progression) and errors in replicating the chosen catheter configuration, resulting in suboptimal microsphere deposition^23,24^. Moreover, the scout procedure is limited to evaluating a single injection strategy per injection location, as accumulated microsphere lodging complicates iterative optimization of the catheter configuration.

We propose an augmented TARE protocol that integrates real-time ultrasound feedback by using ultrasound-contrast microbubbles as microsphere precursors for improved control over the microsphere biodistribution. Our recently conducted in-vitro study demonstrated that ultrasound-contrast microbubbles and non-radioactive ^165^Ho-microspheres follow similar trajectories downstream of the first major arterial bifurcations when injected using the same catheter configuration^25^. Given that these microbubbles are sufficiently small to pass through the capillary bed (mean diameter of 2.5 µm, with 90% of all microbubbles smaller than 8 µm^26^), unlike embolizing microspheres, sequential microbubble injections can be used to iteratively optimize the catheter configuration for improved tumor targeting during TARE. The resulting microbubble biodistribution can then be quantified using dynamic contrast-enhanced ultrasound (DCE-US), thereby establishing what we term the DCE-US-guided TARE approach. However, it remains to be shown whether microbubbles can accurately predict the microsphere biodistribution in a realistic perfused liver model.

This study presents a proof-of-concept for DCE-US guided TARE in healthy ex-vivo machine-perfused porcine livers. We show that real-time DCE-US feedback can be used to optimize catheter positioning for targeting specific regions. Microsphere injections after optimization produce depositions that correlate with the DCE-US predictions, demonstrating that microbubbles can accurately predict the microsphere biodistribution. These results suggest that DCE-US could be a promising tool to improve treatment planning for TARE, although the findings are preliminary and clinical feasibility remains to be established.

## Methods

### Approach

**Fig. 1:**
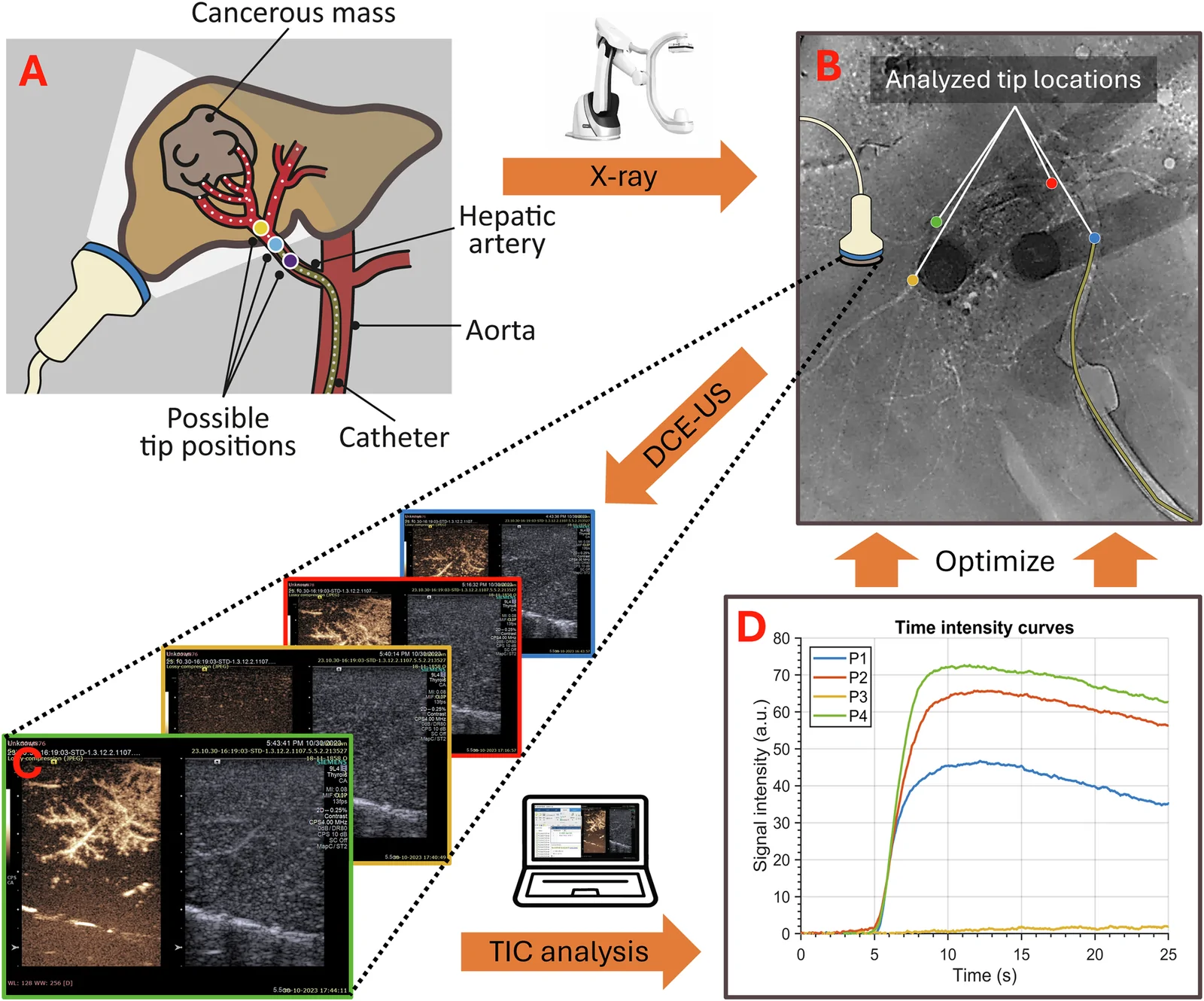
Overview of the envisioned clinical dynamic contrast-enhanced ultrasound-guided radioembolization procedure. **A** After identification of a tumor (or target site in this study), the ultrasound transducer is focused on this region and **B** the catheter is inserted under fluoroscopic guidance to several locations that are deemed suitable for targeting the tumor. **C** Microbubbles are injected at these catheter positions, **D** which are directly quantified to provide real-time feedback of the suitability of a catheter position by the resulting peak of the time intensity curve (TIC). The optimum catheter positions can then be chosen for the injection of radioactive 166Ho microspheres.

The envisioned iterative optimization of the catheter configuration in DCE-US-guided TARE is illustrated in Fig. 1. Three healthy porcine livers were used to demonstrate the augmented TARE procedure. An arbitrary location, such as part of a liver lobe, was designated as the target region to which the microsphere biodistribution should be confined. An ultrasound transducer was fixed to image this region (Fig. 1A) to evaluate catheter configurations in terms of their ability to deliver microbubbles to the target region (Fig. 1B). Real-time quantification of the microbubble presence in tissue was performed using DCE-US and time intensity curve (TIC) analysis (Fig. 1C, D). Using the optimized catheter configuration, ^165^Ho-microspheres were then injected, and their resulting biodistribution was assessed using MRI to evaluate the accuracy of DCE-US-based treatment planning.

### Organ and blood procurement

Porcine livers were retrieved from a local slaughterhouse (Vion Groenlo BV, Groenlo, The Netherlands). Slaughtering was done following the guidelines of the Netherlands Food and Consumer Product Safety Authority (NVWA). The liver was prepared according to a custom protocol inspired by the procurement protocol for human livers of XVIVO (XVIVO B.V., Groningen, The Netherlands), which is described by Snoeijink et al.^27^. Within 30 minutes from cardiac arrest^28^, the portal vein (PV) was cannulated and the inferior vena cava was dissected in order to flush the liver through the PV cannula with a cold (1–5 °C) saline solution (NaCl 0.9% infusion solution, B. Braun, Melsungen, Germany). The flush included an initial 1 L of heparinized (Heparin-Natrium LEO, LEO Pharma GmbH, Ballerup, Denmark) saline solution (25.000 IU/L) to prevent blood clot formation in the liver vasculature, followed by 2 L of non-heparinized saline solution to flush out remaining blood. After cannulation of the PV and initiation of the flush, the liver was isolated and put on ice for transport. For the perfusate, 5 to 10 L of non-autologous blood was collected as well, with additives including 6 mL/L of saline solution, 1.8 mL/L of 50% glucose solution (G-50, B. Braun, Melsungen, Germany), and 15.000 IU/L heparin.

After transport, the aorta was cannulated on ice, and all arteries from the aorta up to the bifurcation of the hepatic artery (HA) were ligated, such that the blood supply through the aorta and HA was only directed to branches that supplied the liver^27^. Excess tissue was dissected, and the liver was weighed (Kitchen scale 15 kg, Hendi, De Klomp, The Netherlands). Cold ischemia time was limited to 4 hours, including transport.

The liver condition was investigated after liver preparation by using magnetic resonance imaging (MRI). A 1.5 T MRI (MAGNETOM Aera, Siemens, Erlangen, Germany) with an 18-channel body array coil and a 32-channel spine coil was used to acquire anatomical images using a three-dimensional T1-weighted DIXON sequence. Sequence parameters were: TR/TE1/TE2 = 6.75/2.39/4.77 ms, flip angle = 10°, field-of-view = 400 × 364 mm, voxel size = 1.1 × 1.1 × 1.1 mm, number of slices = 128.

### Machine perfusion: the LiverTwin

An experimental machine perfusion platform developed in-house, coined the LiverTwin, was used for ex-vivo normothermic perfusion of porcine livers. The main components of the system are shown in Supplementary Materials 1**(**Fig. S1**)**. For the HA side, flow was facilitated by an iLA active console (**I**) (Xenios AG, Heilbronn, Germany) with a ¼ inch DP3 mixed-flow rotary pump head (**II**), which can provide a pulsatile flow with control over flow rate, amplitude, frequency, and systolic fraction. For the PV side, flow was facilitated by a Maquet Rotaflow pump console (**III**) (Maquet Cardiopulmonary GmbH, Rastatt, Germany) with a ⅜ inch centrifugal pump head (**IV**), which can regulate non-pusatile flow rates. Two hollow-fiber oxygenators with integrated heat exchangers were used: on the HA side a PLS-i (**V**) (Maquet, Rastatt, Germany) and on the PV side a MiniLung petite (**VI**) (MEDOS Medizintechnik AG, Stolberg, Germany). The blood temperature was regulated by the integrated heat exchangers and a Hico Variotherm 550 heater/cooler system (**VII**) (Hico Medical Systems, Köln, Germany).

**Fig. 2:**
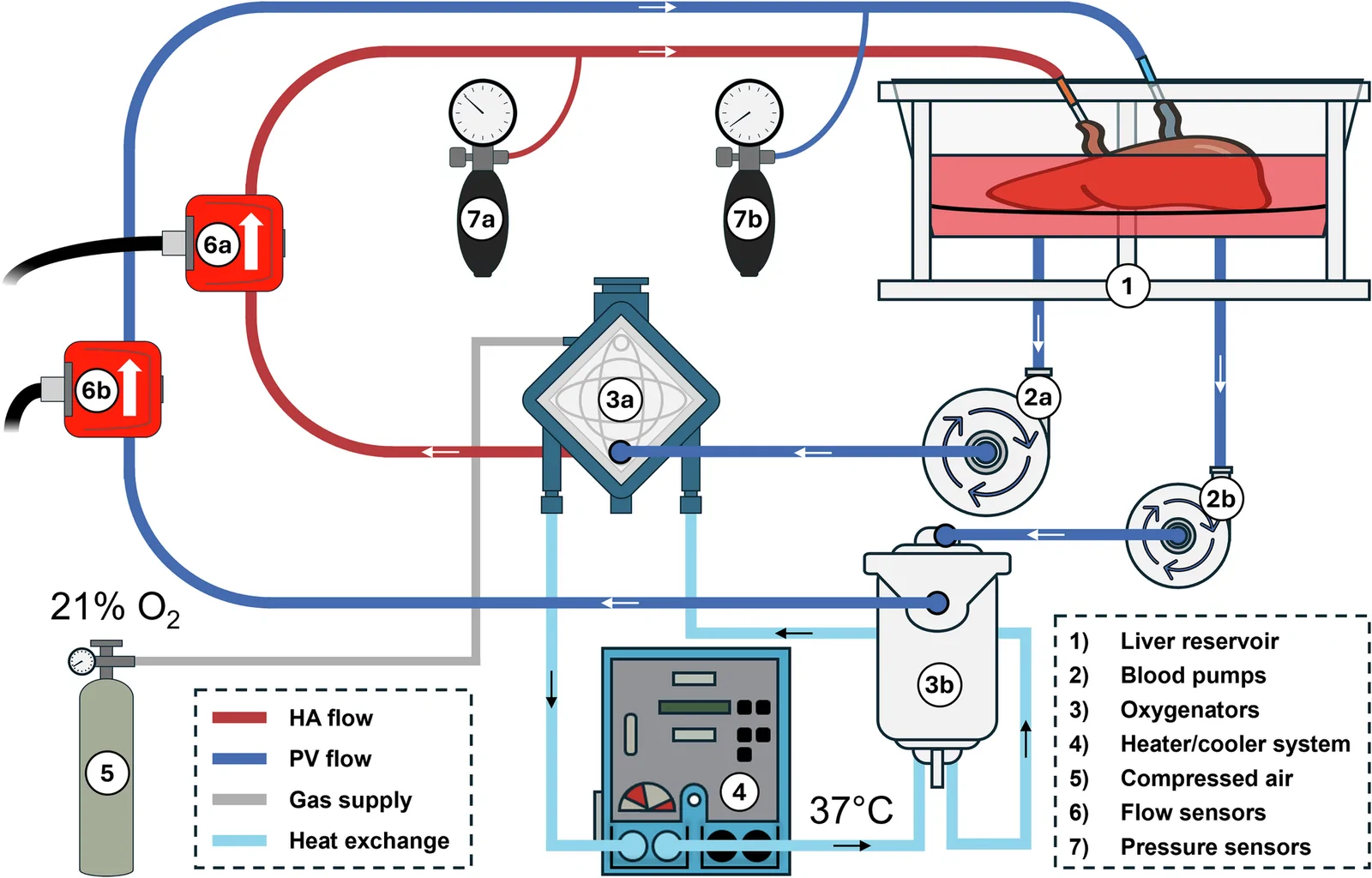
Schematic overview of the setup used for ex-vivo liver perfusion. Flow circuits for ex-vivo normothermic machine perfusion of porcine livers, including the hepatic arterial (HA) and portal venous (PV) blood flow circuits for the dual blood supply to the liver, a gas supply to the HA oxygenator, and the heat exchange flow circuits connected to both oxygenators.

The dual blood supply to the liver was facilitated by creating the flow circuits as shown in Fig. 2. The liver was placed in a custom-built reservoir (**1**) with a height-adjustable organ net that facilitates multimodal medical imaging. The liver remained blood-submerged throughout the experiment to support MR imaging and prevent dehydration of the superficial tissue. The outlet connections of the reservoir were located at the base to ensure direct recirculation of sedimenting red blood cells. From the reservoir, blood is pumped through two separate blood flow circuits for the HA and PV side using rotary blood pumps (**2a**, **2b**), which was then directed to the oxygenators (**3a**, **3b**). Tempered water was supplied to both oxygenators by the heater/cooler system to maintain a blood temperature of 37 °C (**4**). Compressed air (21% O_2_) was supplied to the oxygenator on the HA side (**5**), whereas the oxygenator on the PV side only functioned as a heat-exchanger to mimic the lower oxygen saturation in the PV^29^. Ultrasound clamp-on flow sensors (Sonoflow CO.55/140 v2.0, Sonotec GmbH, Halle, Germany) were installed downstream of the oxygenators on both the HA and PV side (**6a**, **6b**). This placement enabled correction for flow damping caused by the HA side oxygenator, ensuring that the correct flow profile reached the arterial liver vasculature. The blood pressure was monitored by including sphygmomanometers (Durashock DS54, Welch Allyn, Skaneateles Falls, US) on both sides (**7a**, **7b**), attaching the sensors to pressure tubing (Edwards Lifesciences B.V., Breda, The Netherlands) that was connected close to the HA and PV cannulas.

Blood gas analysis was performed by using an i-STAT Alinity blood gas analyzer (Abbott Point of Care, Chicago, IL, USA) with CG4+ and CG8+ cartridges, allowing monitoring of several metabolic parameters (e.g., lactate, pH, hemoglobin concentration, and pCO_2_/pO_2_).

### Perfusion protocol

Normothermic machine perfusion of the liver was performed according to a modified version of the controlled oxygenated rewarming DHOPE-NMP protocol^30^. An overview of this protocol can be found in Table 1, which was established by logging the temperature over several liver perfusion experiments during the development phase using the integrated thermocouples of the ultrasound clamp-on flow sensors (Sonoflow CO.55/140 v2.0, Sonotec GmbH, Halle, Germany). Initially, the system was primed with saline solution to de-air the oxygenators and wet the tubing. Then, the saline solution was replaced by the blood perfusate, and the liver was submerged in the reservoir. The system was connected to the HA and PV cannulas, and perfusion was initiated. At the start of perfusion, 80 mL of 8.4% sodium bicarbonate (Fresenius Kabi Nederland B.V., Huis Ter Heide, The Netherlands) was added to correct for acidosis. Flow, pressure, and air flow were logged and adjusted in accordance with Table 1 aiming to establish normothermic perfusion after 50 minutes.

**Table 1:** Controlled oxygenated rewarming normothermic machine perfusion (COR-NMP) liver perfusion protocol, with a constant portal vein (PV) flow, a pulsatile hepatic artery (HA) flow with the given flow limits corresponding to 20% pulsatility, and a sweep gas flow to the hepatic artery side oxygenator with pressurized air (21% O_2_)

COR-NMP liver perfusion							
	Rewarming				Normothermic		
Time (hh:mm)	**00:00**	**00:15**	**00:30**	**00:45**	**00:50**	**→**	**END**
Temperature (°C)	20	25	30	35	37	…	37
PV flow (mL/min)	300	450	750	1150	1300	…	1300
HA Flow (mL/min)	80 – 120	120 – 180	200 – 300	300 – 450	360 – 540	…	360 – 540
Sweep gas flow (mL/min)	100	150	200	300	500	…	500

Perfusion was terminated (END) after approximately 4 hours for all experiments.

### Fluoroscopy: catheter navigation and analysis points

**Fig. 3:**
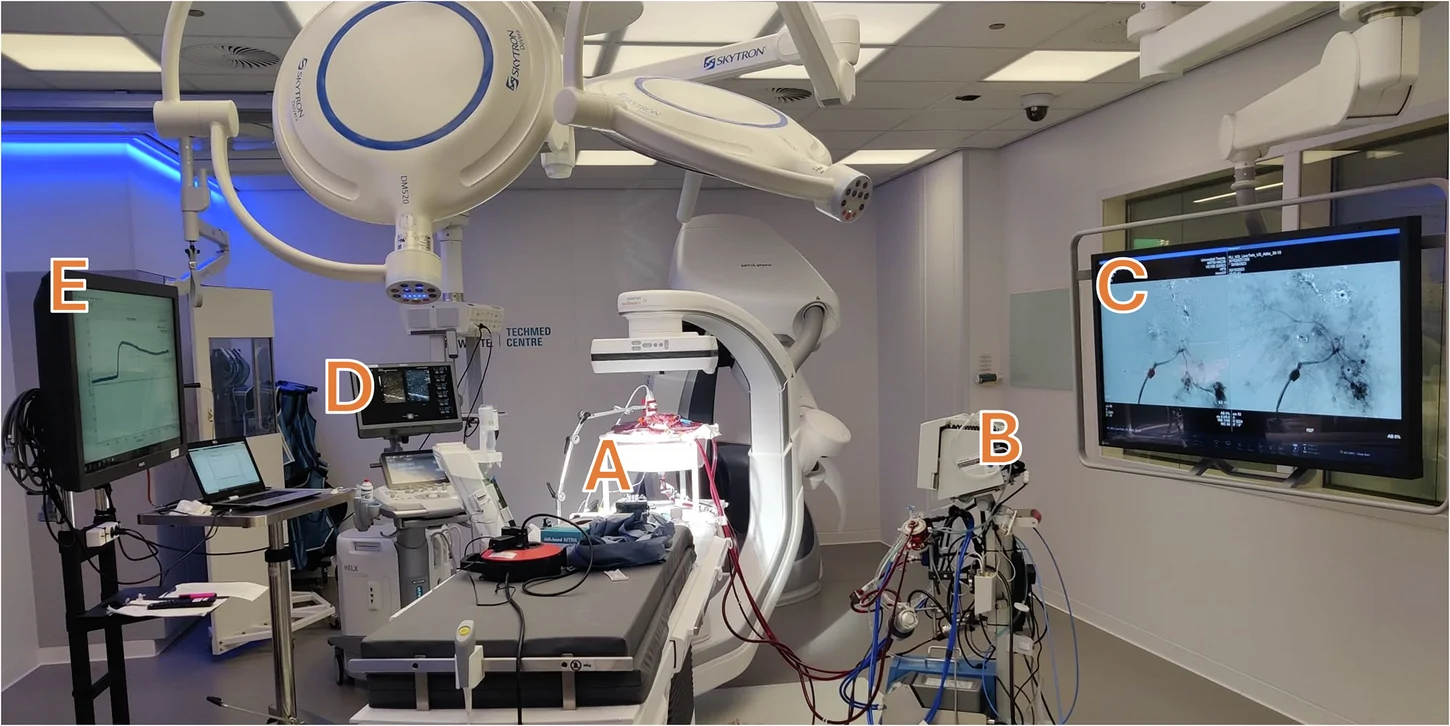
Overview of the experimental setup in the hybrid operation room. **A** A perfused porcine liver raised above the reservoir for visualization purposes, including a 9L4 ultrasound imaging transducer fixed at the target location, **B** The LiverTwin perfusion platform for normothermic oxygenated blood perfusion of the porcine liver, **C** Fluoroscopy images for microcatheter guidance using a digital subtraction angiography (DSA), **D** An ACUSON S2000 ultrasound system showing both a contrast mode and a B-mode image and **E** A display showing the analyzed ultrasound data in TICs for multiple injection locations. Note that a second ultrasound system was used in two of the three measurements.

The clinical radioembolization setting was replicated by conducting the experiments in a hybrid operating room, as shown in Fig. 3. The microbubbles and microspheres were injected using a microcatheter (Progreat 2.7 Fr. 130 cm, Terumo Interventional Systems, Zaventem, Belgium) that is commonly used in TARE^22^. The microcatheter was inserted through a hemostatic valve installed directly upstream of the arterial cannula and advanced within the arterial vasculature to investigate multiple injection positions. Navigation of the microcatheter was performed under fluoroscopic guidance (ARTIS Pheno, Siemens Healthineers, Erlangen, Germany). Digital subtraction angiography (DSA) was performed using an iodine-based contrast agent (Iomeron® 300 mg/ml, Bracco Imaging, Milan, Italy), administering 60 mL at 3 mL/s. This DSA was then inverted and overlaid onto live fluoroscopy imaging to create a roadmap for visualization and navigation of the microcatheter within the vasculature.

In each experiment, the catheter was initially positioned in the proper HA. For subsequent catheter positions, the catheter was navigated past downstream bifurcations to find the artery feeding the target area. In the third experiment, small deviations in the catheter position were also analyzed after identifying the feeding artery, to investigate the sensitivity of DCE-US to the catheter position.

### Dynamic contrast-enhanced ultrasound: prediction of ^165^Ho-microsphere biodistribution

**Fig. 4:**
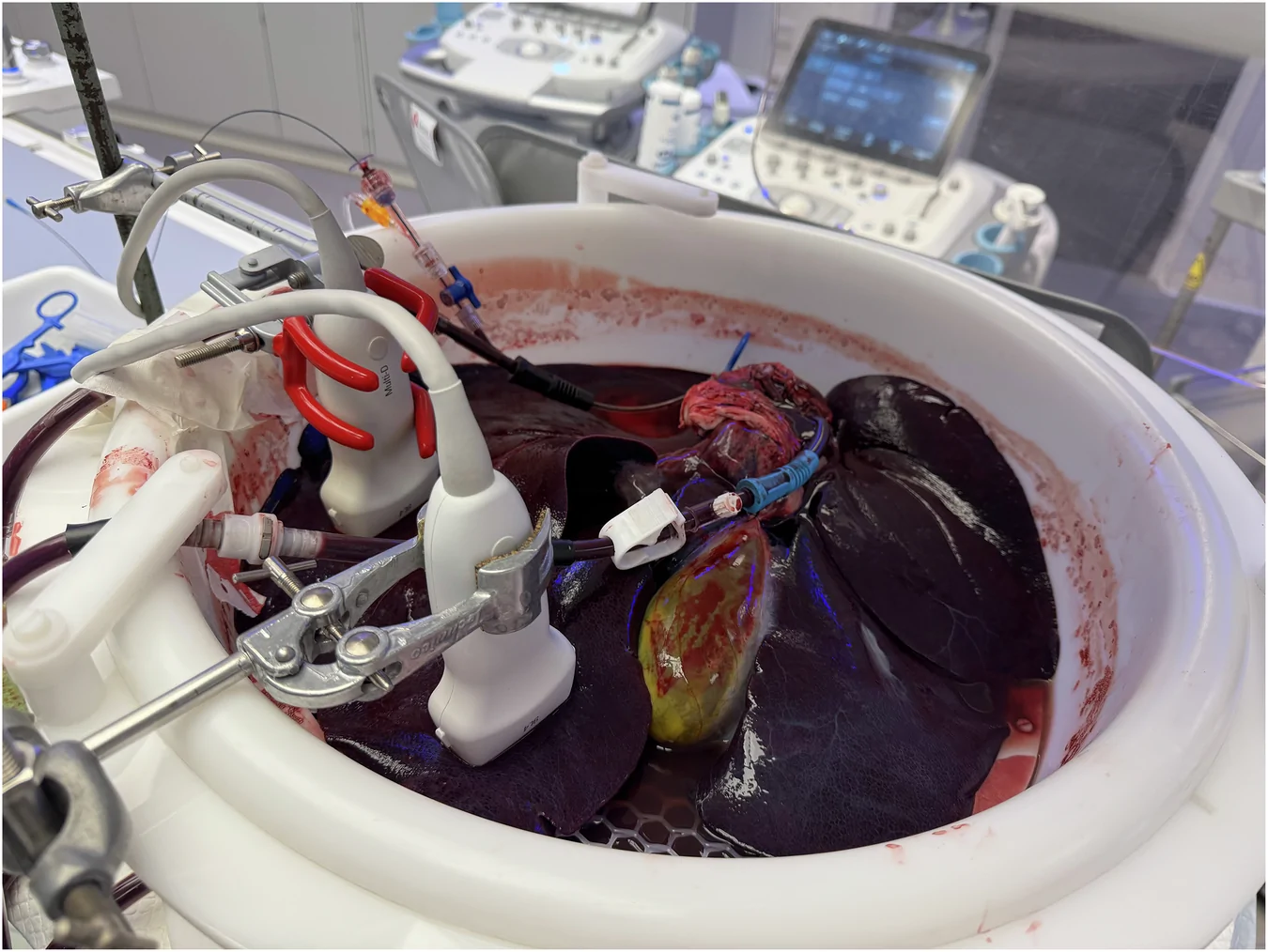
Overview of the target and reference ultrasound probe fixation. Photo of an ex vivo perfused porcine liver used for development of the methods, showing two fixed 9L4 ultrasound transducers positioned at the target and reference sites for optimized targeting while monitoring off-target deposition in an adjacent lobe.

Before each experiment, a target region was selected based on an initial DSA to represent a treatment area within the healthy liver tissue. A 9L4 ultrasound transducer connected to the Acuson S2000 ultrasound system (Siemens Healthineers, Erlangen, Germany) was fixed to image at this location, enabling assessment of a chosen catheter position in its ability to target the treatment area. During the fixation of the transducer, its positioning was carefully selected for the presence of major vessels, which may introduce signal non-linearity and should be avoided in DCE-US comparison^31^. In experiments 2 and 3, a second 9L4 transducer was fixed to image at a location close to the target site, serving as a reference site that represents healthy liver tissue where microsphere deposition should be avoided (Fig. 4). This reference transducer was used solely for experimental quantification and comparison and is not envisioned for use in the clinical setting.

Contrast-enhanced ultrasound imaging was performed using the clinical Acuson S2000 ultrasound system. A non-linear pulsing scheme, Contrast Pulse Sequencing, was used, with a power of 0.25%, transmit frequency of 4 MHz, imaging depth of 3.5–4.5 cm, frame rate of 13 Hz, dynamic range of 80 dB, and contrast gain of 10 dB. The rationale for these settings was presented in previous work^32^. The imaging depth was set to include the full cross-section of the lobe, varying per liver. This resulted in a mechanical index (MI) between 0.07 and 0.09 during acquisition, ensuring that MI < 0.1, which is associated with minimal microbubble destruction^33^. The focus depth was set as deep as possible to achieve the most homogeneous acoustic field across the imaged region. The acquisition time was set to 60 seconds to capture the wash-in and wash-out of microbubble injections.

The clinically approved ultrasound contrast agent Sonovue (Bracco, Geneva, Switzerland) was used for DCE-US acquisition^34^. The microbubble injection solution was prepared by resuspending a Sonovue vial with 5 mL of phosphate-buffered saline (PBS) solution (Dulbecco’s phosphate-buffered saline, Sigma-Aldrich, St. Louis, USA), swirled until suspension was reached visually. The solution was drawn into a 1 mL syringe, which was gently swirled before each injection to keep the microbubbles suspended. The injection solution was then loaded into the microcatheter by manually injecting 0.2 mL of the injection solution into tubing coupled to the microcatheter. Subsequently, a controlled microbubble injection was realized by initiating a 5 mL PBS flush using an injection pump (PHD ULTRA, Harvard Apparatus, Holliston, USA) at a steady flow rate of 10 mL/min.

Real-time feedback on microbubble arrival dynamics from a chosen catheter position was achieved by DCE-US. With this technique, microbubble injections were quantified directly after acquisition by a time-intensity curve (TIC) algorithm developed in-house. The direct quantification was facilitated by feeding the live ultrasound images into this algorithm via a frame grabber (HDMI video capture, Earkings, Bemmel, The Netherlands). Before the first acquisition, a region of interest (ROI) was defined on the B-mode image to capture the contrast-enhanced imaging. After initiating the microbubble injection, live ultrasound frames were converted to grayscale and stored for analysis. Immediately after acquiring the last frames, the brightness values of each frame were summed to quantify the intensity. The baseline offset given by the intensity of the first frame was subtracted from each frame to obtain the final TIC. Optimization of the catheter position was performed iteratively based on peak intensity, as illustrated in Fig. 1.

The TICs were plotted directly on a screen positioned next to the ultrasound system. At each catheter position, three injections were analyzed to determine repeatability. Before each injection, residual microbubbles from previous injections were eliminated using a high-pressure ultrasound burst to destroy the microbubbles. For each measurement, the recorded TIC was added to the measurement set to allow direct analysis of the new catheter position with respect to the previous positions. For experiments 2 and 3, TICs of the reference position (control) were also determined in real-time, plotted in the same figure as the target TICs for comparison. For all experiments, the TIC with the highest peak intensity was highlighted on the screen, with its corresponding catheter position indicated to directly identify the optimal catheter placement.

### Holmium microsphere injection & analysis

Non-radioactive ^165^Ho-microspheres were injected at the optimized catheter position as identified via DCE-US analysis. The injected solution consisted of 300 mg ^165^Ho in a poly-L-lactic acid (PLLA) shell (QuiremSpheres^TM^, Quirem Medical B.V., Deventer, The Netherlands), suspended with 4.5 mL PBS and 0.5 mL of pluronic buffer solution (0.1% pluronic, Quirem Medical B.V., Deventer, The Netherlands)^35^. The ^165^Ho-microspheres were injected using the injection pump with the same injection settings as those used for the microbubbles to avoid differences in biodistribution^9,12^, using microsphere injection fractions of 0.5 mL followed by a 5 mL PBS flush.

Following administration of the ^165^Ho-microspheres, perfusion of the liver was stopped, and the liver was transported back to the MRI to assess liver condition after perfusion and to determine the ^165^Ho-microsphere biodistribution. Holmium-sensitive T2*-weighted images were acquired using a multi-echo gradient recalled echo (mGRE) sequence, which were then stored for post-processing to assess the microsphere biodistribution. Sequence parameters were: TR/TE1/ΔTE = 1040/1.7/2.4 ms, number of echoes = 12, flip angle = 90°, field-of-view = 400 × 400 mm, voxel size = 1.56 × 1.56 mm, slice thickness = 4.0 mm, number of slices = 34. R2* maps were generated by fitting the multi-echo data within the liver to a mono-exponential signal model, in which increased values indicate the presence of ^165^Ho-microspheres^36^. Baseline liver signal was removed through thresholding at the maximum value measured in lobes without visible microsphere deposition, followed by median filtering with a kernel size of 5 × 5 pixels to improve visualization of the spatial biodistribution of the microspheres. The resulting R2* maps were finally normalized to the maximum R2* value, yielding a qualitative ^165^Ho-microsphere biodistribution map within the liver.

The inclusion of the reference ultrasound transducer in the second and third cases enabled comparison between the DCE-US prediction and the MRI-derived ^165^Ho-microsphere biodistribution. For quantification, regions of interest were defined on the R_2_* maps at the transducer positions, with the physical transducer footprint extended by a margin of 1.5 times the transducer size to account for uncertainty in transducer placement. The transducer position was determined either from fluoroscopy or, if the transducer was outside the fluoroscopy imaging region, from a photograph of the placement on the liver, with the gallbladder used as an anatomical landmark.

### Statistics and Reproducibility

Three ex vivo livers were analyzed on a case-by-case basis. For each case, all generated TICs were normalized to the maximum peak intensity measured at the target position within that case. For each injection location, mean peak intensities were calculated from three measurements, and the corresponding standard deviations were reported (±σ). In addition, two ratios were computed using the mean peak intensities: the relative signal improvement with respect to the first injection location, calculated separately for the target and reference positions (T_P_/T₁ and R_P_/R₁), and the target-to-reference signal ratio (T_P_/R_P_).

## Results

### Real-time DCE-US prediction

**Fig. 5:**
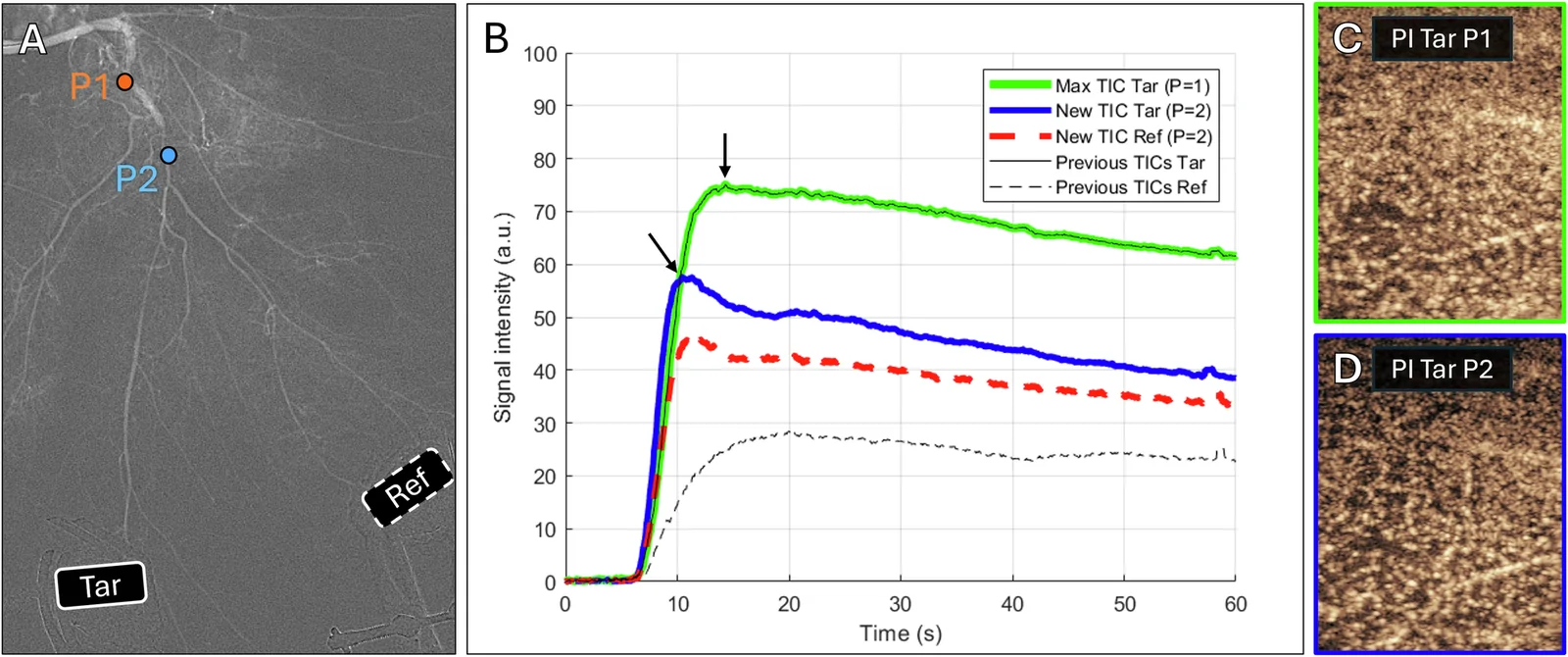
Real-time DCE-US prediction example. **A** fluoroscopy image with the two analyzed catheter positions P1 and P2, **B** time-intensity curve (TIC) analysis displaying the new TICs from P2 for the target (blue) and reference (red) transducers, previously recorded TICs (target: continuous black, reference: dashed black), and the target transducer TIC with the maximum peak intensity (green), and **C**, **D** contrast-enhanced ultrasound images at peak intensity (PI, time instance indicated in **B**) for the target and reference transducers corresponding to positions P1 and P2, respectively.

**Fig. 6:**
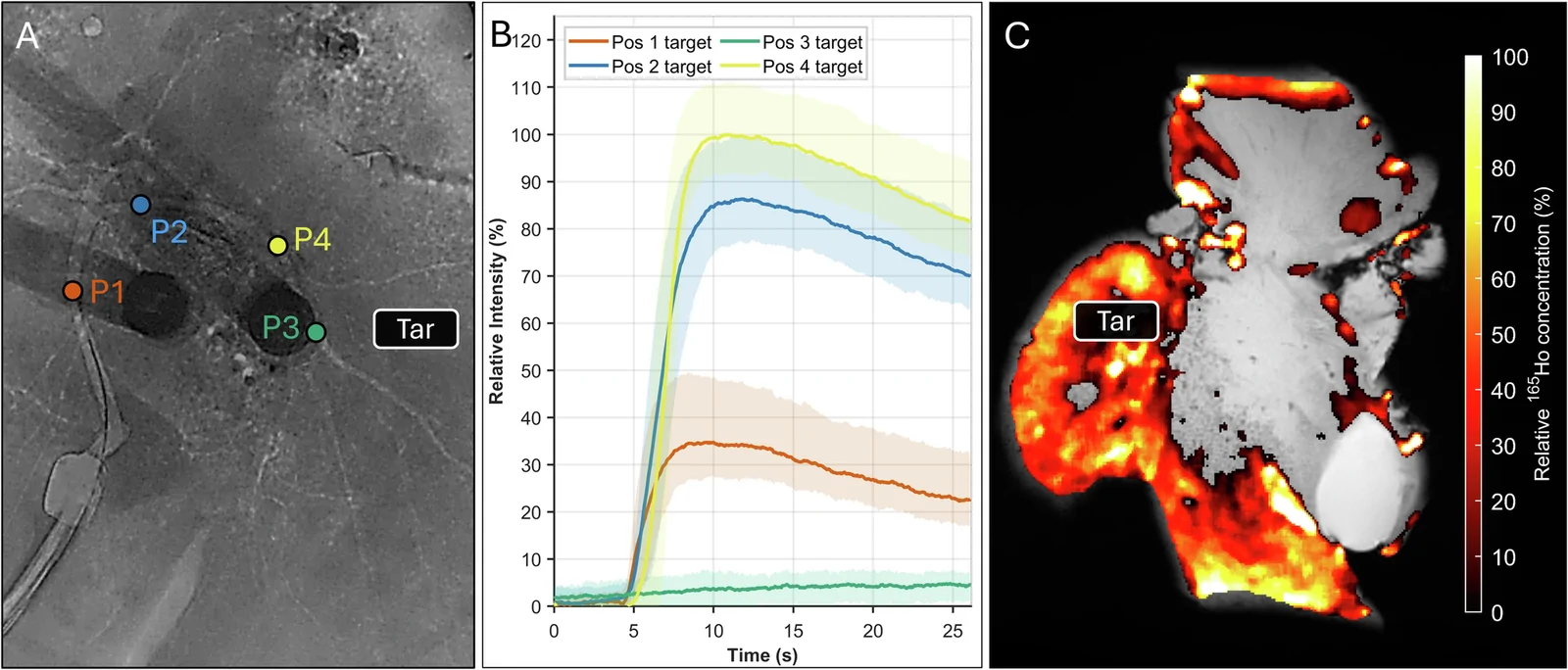
Overview of fluoroscopy data, TICs after microbubble injection and resulting ^165^Ho-microsphere distribution for case 1. **A** Fluoroscopy roadmap image, showing the location of the ultrasound transducer (Tar) and the analyzed catheter positions (P1, P2, P3 & P4), **B** dynamic contrast-enhanced ultrasound, averaged time-intensity curves from microbubble injections at the analyzed catheter positions including standard deviation as error bands (1 σ), and **C** MRI, T2*-weighted scan with thresholded R2* overlay, illustrating relative deposition of ^165^Ho-microspheres and the ultrasound transducer position.

Direct feedback on injections at different catheter positions was achieved using real-time DCE-US prediction through TIC analysis, as illustrated in Fig. 5 with data from an early experiment used for method development and optimization. The figure presents an intermediate result of the experiment, showing measurements from both the target and reference ultrasound transducers following injections at two catheter positions (P1 and P2), marked on the fluoroscopy image in Fig. 5A. The fluoroscopy images that have been used for determining the exact catheter and ultrasound transducer positions can be found in Supplementary Materials 2**(**Figs. S2**, S3)**. The corresponding TICs are shown in Fig. 5B, which were automatically updated after each injection. In this case, the newly recorded TICs from P2 are shown, with the target transducer signal in red and the reference transducer signal in blue, alongside the previously acquired TICs from P1, where continuous lines represent the target transducer and dashed lines stand for the reference transducer. The TIC with the highest peak intensity, along with its associated catheter position (P1), is highlighted. Contrast-enhanced ultrasound images at peak intensity for the target transducer are given in Fig. 5C and D, emphasizing the increased signal intensity following injection at P1 compared to P2. (Fig. 6)

#### Case 1

The liver used in this experiment had a weight of 2,678 g. HA and PV flow rates were slightly below the targeted values, measuring 900 mL/min and 300 ± 60 mL/min, respectively. Sweep gas flow through the oxygenator connected to the HA circuit was maintained at 500 mL/min throughout the procedure with an oxygen content of 21%. Pressure and blood gas parameters were not monitored during this experiment. Post-perfusion anatomical and Holmium-sensitive MR scans are provided in Supplementary Materials 3**(**Figs.  S17, S18**)**.

**Fig. 7:**
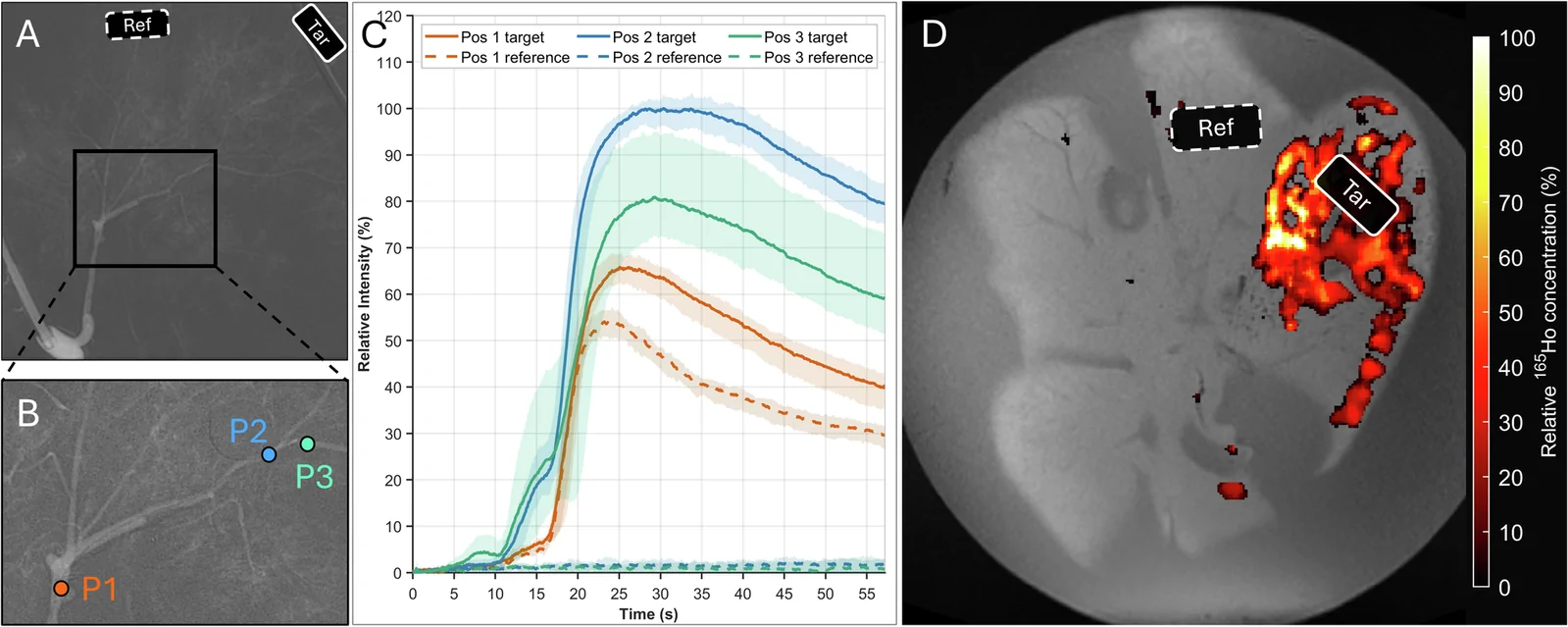
Overview of fluoroscopy data, TICs after microbubble injection and resulting ^165^Ho-microsphere distribution for case 2. **A** Fluoroscopy roadmap image, showing the approximate target (Tar) and reference (Ref) ultrasound transducer locations, **B** fluoroscopy roadmap image, zoomed-in with analyzed catheter positions (P1, P2 and P3) indicated, **C** dynamic contrast-enhanced ultrasound, averaged time-intensity curves for the target and reference transducer curves from microbubble injections at the analyzed catheter positions including standard deviation as error bands (1 σ), and **D** MRI, T2*-weighted scan with thresholded R2* overlay, illustrating relative deposition of ^165^Ho-microspheres and the target and reference ultrasound transducer positions.

A total of four catheter positions were evaluated, indicated in the fluoroscopy image in Fig. 7A by P1 to P4 Representative fluoroscopy images for each catheter position are provided in Supplementary Materials 2**(**Figs. S4–**S7)**. Peak intensity statistics are given in Supplementary Materials 6**(**Table S7**)**. The mean standard deviation was 8.8%. From position P1 to P4, peak intensity increased by T_4_/T_1_ = 2.9. At position P3, the signal dropped to near-zero, suggesting that the catheter had been advanced beyond the feeding artery supplying the target area. Placement of the catheter in front of the feeding artery at position P4 resulted in the highest peak intensity, where ^165^Ho-microspheres were injected. The MRI data in Fig. 7C demonstrates high signal within the target lobe, corresponding to a relatively high microsphere concentration in this lobe. A substantial microsphere concentration was also observed in the adjacent lobe, as a result of placing the catheter before, rather than inside, the feeding artery.

#### Case 2

The liver used in this experiment weighed 2453 g. The oxygenated rewarming protocol was applied, and hemodynamic parameters during rewarming are given in Supplementary Materials 4**(**Table S1**)**. Upon achieving the target HA and PV flow rates of 450 ± 90 mL/min and 1300 mL/min, respectively, measured pressures were 240 mmHg (HA) and 122 mmHg (PV). Sweep gas flow through the oxygenator connected to the HA circuit was increased from 100 to 500 mL/min during rewarming and subsequently maintained at this level, with an oxygen content of 21%. Blood gas parameters were monitored at several time instances during the experiment and are provided in Supplementary Materials 5**(**Tables S3, S4**)**. Pre- and post-perfusion anatomical and Holmium-sensitive MR scans are provided in Supplementary Materials 3**(**Figs. S19–S2^1^**)**.

**Fig. 8:**
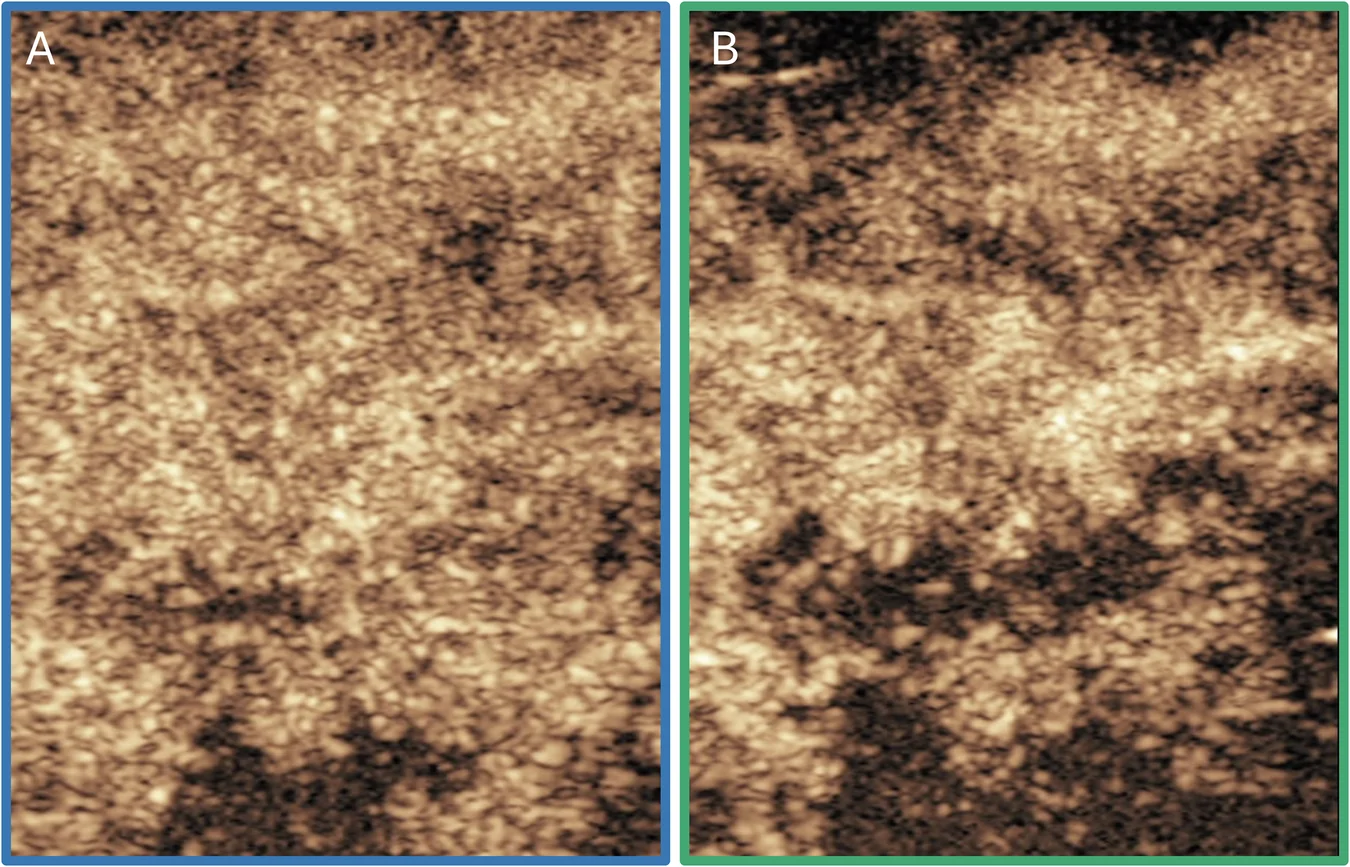
Contrast-enhanced ultrasound images at peak intensity of the time-intensity curves for Case 2. **A** position 2 (Pos 2 target), a homogeneous microbubble biodistribution corresponding to the higher intensity curve, and **B** position 3 (Pos 3 target), a heterogeneous microbubble biodistribution associated with the lower intensity curve.

A total of three catheter positions were evaluated, with an overview of the results shown in Fig. 7. Fluoroscopy images for each catheter position are provided in Supplementary Materials 2**(**Figs S8–**S10)**. Peak intensity statistics are given in Supplementary Materials 6**(**Table S8**)**. The mean standard deviation was 4.0%. From position P1 to P2, peak intensity at the target area increased by T_2_/T_1_ = 1.5. For positions P2 and P3, the signal in the reference area dropped to R_2_/R_1_ = 0.04 and R_3_/R_1_ = 0.03, indicating catheter advancement beyond the feeding artery of the reference area. From position P2 to P3, signal intensity decreased noticeably due to only targeting a part of the ROI, evidenced by the heterogeneous biodistribution of microbubbles resulting from injection at position P3, as shown in Fig. 8. While a higher target-to-reference signal ratio was found for position P3 (T_2_/R_2_ = 48, T_3_/R_3_ = 58), microspheres were injected at position P2 for better coverage of the target region. Within the analyzed regions, a microsphere deposition was found to be high at the target transducer and minimal at the reference transducer (99.2% vs. 0.8%). For the TIC analysis, a similar fraction was found comparing peak intensities from the target transducer and reference transducer (98% vs 2%). Low R2*-values were also observed in the reference area and other liver regions.

#### Case 3

The liver used in this experiment weighed 2,930 g. Hemodynamic parameters during rewarming are given in Supplementary Materials 4**(**Table S2**)**. Upon achieving the target HA and PV flow rates of 450 ± 90 mL/min and 1,300 mL/min, respectively, measured pressures were 168 mmHg (HA) and 80 mmHg (PV). Sweep gas flow through the oxygenator connected to the HA circuit was increased from 100 to 400 mL/min during rewarming and subsequently maintained at this level, with an oxygen content of 21%. Blood gas parameters were monitored at several time instances during the experiment and are provided in Supplementary Materials 5**(**Tables S5, S6**)**. Pre- and post-perfusion anatomical and Holmium-sensitive MR scans are provided in Supplementary Materials 3**(**Figs. S22–S2^4^**)**.

**Fig. 9:**
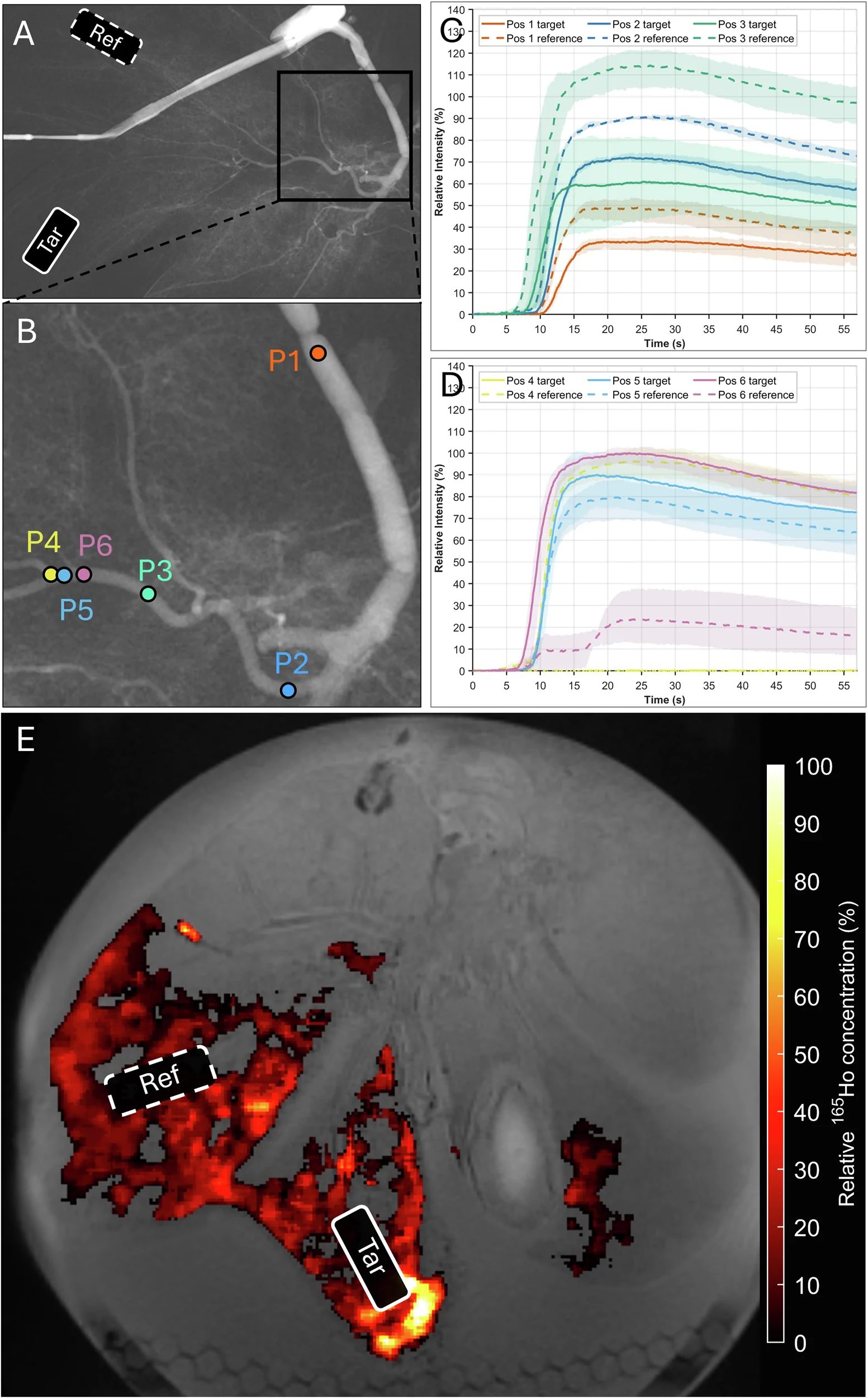
Overview of fluoroscopy data, TICs after microbubble injection and resulting ^165^Ho-microsphere distribution for case 3. **A** Fluoroscopy roadmap image, showing the approximate target (Tar) and reference (Ref) ultrasound transducer locations, **B** fluoroscopy roadmap image, zoomed-in with analyzed catheter positions (P1 to P6) indicated, **C**, **D** dynamic contrast-enhanced ultrasound, averaged time-intensity curves for the target and reference transducer curves from microbubble injections at the analyzed catheter positions including standard deviation as error bands (1 σ), with **C**) for P1, P2 and P3 corresponding to large catheter position deviations and **D**) for P4, P5 and P6 corresponding to small catheter position deviations, and **E** MRI, T2*-weighted scan with thresholded R2* overlay, illustrating relative deposition of ^165^Ho-microspheres and the target and reference ultrasound transducer positions.

A total of six catheter positions were evaluated, with an overview of the results presented in Fig. 9. Fluoroscopy images for each catheter position are provided in Supplementary Materials 2**(**Figs S11–S1^6^**)**. Peak intensity statistics are given in Supplementary Materials 6**(**Table S9**)**. The mean standard deviation was 7.4%. The positions were categorized into two groups, where both major displacements (P1 – P3) and minor displacements (P4 – P6) were evaluated to investigate the sensitivity of the method. From the initial position P1 to the final position P6, peak intensity at the target area increased by T_6_/T_1_ = 3.0, while the peak intensity at the reference area decreased to R_6_/R_1_ = 0.6. For positions P1 to P5, the signal intensity at the reference area consistently exceeded that of the target area. Small displacements of approximately 1 mm resulted in significant differences between target and reference signals. Microspheres were injected at position P6, where the target-to-reference signal first exceeded 1 (T_6_/R_6_ = 3.4), resulting in a microsphere deposition in the target area and the reference area (70.2% vs. 29.8%), based on analysis regions defined similarly to the previous case. For the TIC analysis, peak intensities from the target transducer and reference transducer yielded a slightly different distribution (80.9% vs 19.1%).

## Discussion

In this proof-of-concept study, the potential of dynamic contrast-enhanced ultrasound (DCE-US) guided transarterial radioembolization (TARE) was investigated. We demonstrated that real-time feedback from DCE-US could be used to achieve targeted microsphere biodistributions in the liver. In the first case, a substantial ^165^Ho-microsphere deposition was observed both within the target region as well as in an adjacent lobe, which was not monitored during catheter positioning due to fixation of the transducer during measurements. Notably, as the liver was not fully submerged in blood during MRI acquisition, increased R2* values were also observed along the border of the organ due to susceptibility artefacts that do not reflect the presence of ^165^Ho-microspheres. In the second case, the microsphere deposition was specific to the target region with minimal deposition in the reference region (99.2% vs. 0.8%), closely matching the DCE-US prediction (98% vs 2%). In the third case, a more evenly distributed microsphere deposition was found, which was higher in the target area compared to the reference area (70.2% vs 29.8%), consistent with the DCE-US prediction (80.9% vs 19.1%).

The study was designed to evaluate the performance of DCE-US guided TARE. First, the method demonstrated moderate repeatability, as reflected by distinguishable TICs and a mean standard deviation below 10% across all cases. Some variability in the TICs can be caused by differences in the injected microbubble bolus. As the injected volume and concentration were only controlled visually, improved control over these parameters may improve repeatability. Second, DCE-US demonstrated its potential to achieve a highly specific microsphere biodistribution, largely confined to the target region. In the second and third cases, the microsphere biodistribution was predominantly limited to the areas monitored by the two fixed ultrasound transducers, which were used solely for quantitative assessment of the DCE-US prediction, and only one transducer is envisioned for clinical use. In the first case, where only a single fixed transducer was used, microsphere depositions were also observed outside the target region, suggesting that the catheter position could have been further optimized to achieve a more targeted distribution. Finally, DCE-US demonstrated its advantage as a real-time analysis method by capturing the impact of small deviations in catheter position. Minor displacements near the feeding artery led to significant differences in the peak intensity of microbubble injections. These results not only confirm the high sensitivity of radioembolization to catheter placement but also highlight the key challenge of the current procedure in terms of accurate replication of the catheter configuration between the scout and therapeutic procedures.

The accuracy of the DCE-US prediction method is influenced by several factors. First, the difference in size and density of the microbubbles and microspheres results in different dynamics in the flow^5,26^. Despite these differences, we demonstrated a moderately strong correlation between microbubbles and microspheres trajectories in a previous in-vitro study (p < 0.05, r = 0.88)^25^. The observed discrepancies were most likely caused by these inherent physical differences. Although ^165^Ho-microspheres were used in both the previous and current studies, the DCE-US treatment planning method is also expected to be applicable to ^90^Y-based microspheres, given their comparable physical properties (e.g., size and density)^37^. Second, accurate quantification of microbubble concentrations is a challenge in DCE-US. Current methods for quantitative DCE-US imaging rely on multiple assumptions and simplifications^38^, such as the microbubble size distribution, inter-bubble distances, and the elasticity of the blood vessel wall^39,40^. Our in-house developed algorithm prioritized computational speed to enable rapid assessment, rather than accurate quantification of microbubble concentrations. Additional quantification results from a preliminary experiment (provided in Supplementary Materials 7**(**Figure S25 & S2^6^, Table S10**)**) were compared to those obtained using the commercial software VueBox (Bracco, Switzerland, Geneva), an off-line post-processing application for DCE-US-based tissue perfusion quantification^41^. As VueBox outputs the TICs in dB, a linear regression between both result sets was performed. This fit showed good agreement, with a maximum mean difference of 0.59 dB, indicating that a high computational speed of our DCE-US algorithm is achieved with only minimal loss of accuracy. Finally, DCE-US did not provide a three-dimensional overview of the expected microsphere biodistribution, as the region of analysis is restricted to the two-dimensional imaging plane. In this study, the assessment of different catheter positions relied on comparing their resulting peak intensities, which required transducer fixation and provided limited spatial coverage. To address this limitation, the transducer could be moved during an additional microbubble injection to scan a wider area and help identify the biodistribution boundaries of the candidate catheter configuration.

The MR-based ¹⁶⁵Ho-microsphere biodistribution was evaluated using a custom-made MATLAB script that generated relative dose maps based on the linear R2* relaxivity of ^165^Ho-microspheres^42^. While this approach is conceptually similar to the clinically used Q-Suite software (Quirem Medical B.V., Deventer, The Netherlands), Q-Suite produced unreliable results in this study due to the extended interval between pre- and post-treatment MRI scans. As a result, the relative dose maps could not be directly compared with Q-Suite outcomes, and the accuracy of the generated maps in this study could not be assessed. While the determined relative microsphere distributions visually corresponded to the T2*-weighted multi-gradient MRI scans (see Supplementary Materials 3), it should be considered an approximate representation rather than an exact measurement.

While ex-vivo perfused livers provide a suitable platform to demonstrate the proof-of-concept of DCE-US guided TARE, the platform also presents certain challenges. One key limitation encountered in this study was the difficulty in maintaining a stable liver condition throughout the experiments. This was reflected by suboptimal blood gas analysis results, particularly a consistently low pH (6.85–7.15) outside of the physiological range (7.35–7.45)^43^, and insufficient oxygen saturation (69–86% in case 2; 41–51% in case 3). Although bicarbonate was administered at the start to correct for acidosis, the dosage was likely insufficient to fully restore physiological pH. Despite these metabolic imbalances, the anatomical MR scans did not reveal significant deterioration of liver parenchyma, aside from minor spots in the periphery of certain lobes. Furthermore, the homogeneous biodistribution of microbubbles in the region of interest and peripheral deposition of microspheres suggest that the deterioration in liver condition did not significantly impact the macro- and microvasculature.

Previous studies have explored the use of contrast-enhanced ultrasound to assess the biodistribution in transarterial chemoembolization^44–46^. In these studies, intra-arterial injections of contrast microbubbles were used to identify tumor feeding arteries, providing feedback for the catheter position prior to injection of chemoembolization particles. However, these applications focused on qualitative assessment rather than microbubble signal intensity analysis, as vessel identification relied solely on visualizing the presence of contrast enhancement downstream of arterial bifurcations. Quantitative analysis provides greater control in treatment planning by enabling direct comparison of different catheter configurations. Particularly in cases where contrast enhancement is observed at multiple locations and visual comparison is non-trivial (e.g., Case 3, Fig. 9), quantification supports a more objective and substantiated choice for the optimal catheter configuration. Furthermore, quantitative evaluation facilitates assessment of tumor perfusion, contributing to more comprehensive tumor localization and potentially enabling detection of lesions not evident on other imaging modalities^31,47^. To the best of our knowledge, this study is the first to employ ultrasound-based analysis to quantitatively optimize the biodistribution in locoregional therapy.

Other methods have also been studied to improve prediction of the microsphere biodistribution in TARE. For contrast-enhanced cone-beam CT, a perfusion approach was demonstrated to offer similar predictive accuracy to the conventional ^99m^Tc-MAA-based scout analysis, although both approaches tended to overestimate tumor dose at higher activity levels^48^. A recent retrospective study examined the use of intra-procedural cone-beam CT for predicting the tumor-absorbed dose based on contrast enhancement patterns^49^. While a strong correlation was found for tumor regions, the agreement was moderate for non-tumor tissue. Due to the small patient cohort, results could not be generalized. For MRI, the EMERITUS-1 trial investigated the feasibility of intra-procedural MRI-based dosimetry^50^. In two presented cases, intra-procedural MRI dose mapping suggested alternative treatment strategies. However, repositioning of the microcatheter could not be performed within the MRI, meaning that direct optimization of the catheter configuration was not possible in this setting.

The DCE-US approach shows potential to improve TARE treatment planning, building on the established safety and efficacy of contrast microbubbles, combined with the advantages of ultrasound imaging in this setting, i.e., real-time and portable. Ultrasound is non-ionizing and has a small equipment footprint, making it highly suitable for use in hybrid operating rooms or interventional suites. In such settings, an ultrasound system is often readily available, making the incorporation of DCE-US with a single transducer for enhanced treatment planning feasible. The real-time feedback offered by DCE-US can moreover aid in the diagnosis and grading of tumors^51^, which can be used to further enhance treatment planning and monitoring.

Microbubbles are already well-established in diagnostic imaging and offer a safe alternative to ^99m^Tc-MAA. Microbubbles typically consist of an inert gas core and a stabilizing shell, which are both excreted by expiration and metabolism, respectively^52^. They are associated with a very low prevalence of minor adverse effects (approximately 1 in 3000 examinations^53^). Clinically approved microbubbles such as SonoVue/Lumason (Bracco, Geneva, Switzerland) and Optison (GE Healthcare, Princeton, NJ) have a short intravascular half-life of approximately one minute, depending on the formulation^54,55^. As a result, the DCE-US prediction iterations can be performed on short intervals and microbubble accumulation is unlikely, also because of the low injection volume needed (0.1–0.2 ml per injection). Although these agents are approved for intravenous use and thus intra-arterial use is currently outside their indication for use, several studies have explored intra-arterial administration of various clinically approved microbubble formulations^56–58^.

In current clinical practice, catheter configuration during radioembolization is typically assessed using contrast-enhanced cone-beam computed tomography (CBCT). The introduction of DCE-US offers several advantages over this approach. First, current results indicate that ultrasound contrast microbubbles can predict the microsphere biodistribution, a relationship that has yet to be established for iodinated contrast agents used in CBCT. However, contrast-enhanced ultrasound (CEUS) has already been shown in transarterial chemoembolization to alter treatment planning by identifying superselective feeding arteries that were subsequently targeted instead of those selected based on angiography^59,60^. Second, repeated CBCT acquisitions for assessment and optimization of the catheter configuration increase patient radiation exposure, whereas optimization using DCE-US can be performed with minimal safety concerns. Third, iodinated contrast agents are unsuitable for patients with renal failure and may cause contrast-induced nephropathy^61,62^, whereas ultrasound contrast microbubbles represent a much safer alternative with low rates of adverse effects^51^. Finally, CBCT provides only static images, which limits its utility in predicting depositions in non-tumor tissue and in tumors with different vascularity^49^, whereas CEUS enhancement patterns have been reported to outperform CBCT-derived patterns in delineating tumor boundaries to enhance treatment planning^63^.

Adoption of the DCE-US prediction in TARE is facilitated by a series of applications of contrast microbubbles that have emerged in TARE over the last few years^64^. One of these applications is the monitoring of radiation response after TARE by contrast-enhanced ultrasound (CEUS). For high doses of radiation, tumor blood vessels are destroyed in addition to damaging the DNA^65^, such that changes in the microvasculature can be monitored after TARE. Using CEUS, changes in the microvasculature were detected within 2 weeks after TARE that correlated with the response as monitored by contrast-enhanced MRI^66^. Another technology that has emerged is ultrasound-triggered microbubble destruction (UTMD) for improving radiosensitization of tumors^67^. A randomized pilot clinical trial by Eisenbrey et al. showed promising results for the application of UTMD within TARE, with a greater tumor response for a group of 15 participants that underwent sonoporation in combination with TARE (93%) compared to a control group of 10 participants that solely underwent TARE (50%)^68^. These results further encourage the adoption and integration of the investigated DCE-US guided TARE approach, which could precede UTMD.

While real-time feedback from DCE-US predictions has the potential to improve targeting accuracy compared to the current scout procedure, the latter cannot be omitted entirely. In addition to determining the radiation dose, the scout procedure is essential for detecting extrahepatic depositions and assessing the lung shunt fraction to determine eligibility for TARE. For identifying extrahepatic depositions, contrast-enhanced ultrasound might be employed by scanning organs surrounding the liver where extrahepatic depositions are common, such as the gallbladder and duodenum, for microbubble presence directly after an injection^69^. However, detecting microbubbles in deeper lying organs such as the duodenum may be challenging. For assessment of the lung shunt fraction, contrast-enhanced ultrasound cannot be used in the same way, as microbubbles pass the capillary bed, such that microbubbles are expected in the lungs following injection. Although DCE-US cannot currently replace the scout procedure, future development of alternative methods for estimating the lung shunt fraction may make this feasible.

A limitation of this study is that the DCE-US guided TARE protocol was demonstrated in the context of super-selective TARE, targeting a single lobe or segment of the liver. While this approach is clinically relevant for treating localized tumors, it does not reflect scenarios where a microsphere biodistribution across multiple lobes is required, for example for multifocal tumors. As a result, the applicability of the current protocol remains to be demonstrated in that context.

Another limitation of the study is that microbubble-based prediction was evaluated only for catheter position changes, using standard injection parameters. Alternative injection strategies, such as pulsatile injections, increased injection velocities, or the use of different catheters, may influence microbubble and microsphere biodistribution patterns^70^, potentially improving or compromising the accuracy of the prediction method. On the other hand, the real-time feedback provided by DCE-US could prove to be ideal to assess these parameters, which may instead further improve optimization of the catheter configuration.

Finally, the use of a healthy ex-vivo perfused porcine liver to demonstrate DCE-US guided TARE limits the direct clinical translation of the prediction method. First, the absence of tumors results in different hemodynamic conditions, as microspheres lack a preferential deposition site after arterial injection. Second, the ex-vivo liver allowed direct fixation of the ultrasound transducer, enabling stable and consistent ultrasound imaging. In-vivo, however, factors such as the presence of surrounding organs or structures, respiratory motion may pose additional challenges. Nevertheless, these difficulties can largely be mitigated through current ultrasound scanning protocols, in which imaging from an alternative angle or under breath-hold helps prevent acoustic shadowing (e.g. by the rib cage), as well as through appropriate patient preparation, such as fasting to reduce the effects of bowel gas^71^. It should also be noted that the deeper location of the liver and the presence of tumors in vivo can introduce variations in ultrasound attenuation, for which several strategies exist to enhance the robustness of the demonstrated DCE-US approach, such as defining tumor-specific ROIs or using alternative TIC-derived parameters, including time to peak or area under the curve^72^. Third, microbubble recirculation was not observed in the ex vivo perfused liver. This is likely due to microbubble destruction in the oxygenators and buoyancy toward the free interface of the reservoir, preventing re-entry into the perfusion circuit. In vivo, however, microbubbles recirculate and can produce delayed secondary peaks after approximately 60 seconds^60^, potentially affecting TIC-derived parameters that rely on the wash-out phase and requiring correction of TICs when comparing curves for rapid catheter position optimization in clinical practice. Lastly, although anatomical and vascular structures are similar between porcine livers and human livers, differences in their overall shape may affect the generalizability of the findings in this study.^74^

## Conclusions

Real-time feedback from DCE-US guided TARE enabled effective optimization of catheter positioning to realize targeted microsphere depositions in ex-vivo porcine livers. Microsphere depositions were consistently observed in the target lobe across all livers, and microbubble biodistributions corresponded with the ^165^Ho-microsphere biodistribution between target and reference regions. These results demonstrate that DCE-US guided TARE can enhance microsphere targeting and hold promise for improved treatment outcomes, warranting further clinical investigation.

### Ethics approval and consent to participate

Animals were slaughtered in accordance with NVWA guidelines. The porcine livers used in this study were obtained as by-products of routine slaughter and did not interfere with standard slaughter procedures.

## Supplementary information

Transparent Peer Review file

Supplementary Materials

Description of Additional Supplementary Files

Supplementary Data 1

## Data Availability

All data not related to medical imaging that were generated or analyzed during this study are included in this published article and its Supplementary information files. The source data for all time-intensity curves can be found in the Supplementary Data. Full medical imaging datasets are available from the corresponding author upon request.
