# Supplementary material for: Dynamic contrast-enhanced ultrasound predicts microsphere biodistribution in real-time during transarterial radioembolization
Source: Commun Med (Lond). 2026 Jun 30;6:546. doi: 10.1038/s43856-026-01760-x (PMC13683390; doi:10.1038/s43856-026-01760-x)
Supplement: Supplementary file 1 [file 43856_2026_1760_MOESM1_ESM.pdf]

nature portfolio

Peer Review File

Dynamic contrast-enhanced ultrasound predicts microsphere biodistribution in real-time during transarterial radioembolization

Corresponding Author: Dr Erik Groot Jebbink

This file contains all reviewer reports in order by version, followed by all author rebuttals in order by version.

Attachments originally included by the reviewers as part of their assessment can be found at the end of this file.

Version 0:

Reviewer comments:

Reviewer #1

(Remarks to the Author)
Summary:
This manuscript presents a proof-of-concept study investigating the use of dynamic contrast-enhanced ultrasound (DCE-US) for real-time prediction and optimization of microsphere biodistribution during transarterial radioembolization (TARE). Using ex-vivo perfused porcine livers, the authors injected ultrasound contrast microbubbles at various catheter positions and analyzed time-intensity curves (TICs) to optimize catheter placement. Holmium-165 microspheres were subsequently administered, and their distribution was assessed via MRI. Results demonstrated a strong correlation between microbubble and microsphere biodistribution, suggesting that DCE-US could provide intra-procedural feedback to improve targeting accuracy.

Major Strengths:
1) Innovative Concept and Clinical Relevance
The integration of DCE-US for real-time feedback during TARE addresses a critical limitation of current scout-based planning methods, which suffer from time delays and replication errors.

2) Proof-of-Concept Validation
The study demonstrates a strong correlation between microbubble and microsphere biodistribution (e.g., Case 2: 99.2% vs. 98%), supporting the feasibility of the proposed approach.

3) Practicality and Feasibility
The method leverages widely available ultrasound systems and clinically approved contrast agents, making it potentially easy to implement in hybrid operating rooms without major infrastructure changes.

Major Weaknesses:
1) Limited Clinical Translation
Experiments were performed on healthy ex-vivo porcine livers, which do not replicate tumor vascularity or in-vivo physiological conditions. This significantly limits the direct applicability of findings to human patients.

2) Restricted Scope of Optimization
The study only evaluated catheter position changes using standard injection parameters. Other clinically relevant factors—such as injection velocity, pulsatile flow, or alternative catheter designs—were not explored.

3) Two-Dimensional Imaging Limitation
DCE-US analysis was restricted to a fixed transducer position, providing only a 2D view of microbubble distribution. This limited spatial coverage and contributed to off-target deposition in Case 1.

4) Perfusion Model Instability
Blood gas analysis revealed suboptimal pH and oxygen saturation during perfusion, which could influence vascular dynamics and microbubble/microsphere behavior, introducing uncertainty in experimental validity.

Minor Comments:
1) Figures are well-illustrated but could benefit from clearer labeling of transducer positions and catheter paths for reproducibility.
2) The discussion section is comprehensive but could be condensed for clarity, focusing on clinical implications and future directions.
3) Consider adding quantitative correlation metrics (e.g., Pearson correlation coefficients) between TIC peak intensities and MRI-derived microsphere distributions for stronger statistical support.

Reviewer #2

(Remarks to the Author)
I received the manuscript entitled “Dynamic contrast-enhanced ultrasound predicts microsphere biodistribution in real-time during transarterial radioembolization” authored by
van der Hoek et al. for review.
Th article described the use of contrast enhance ultrasound scans prior to prior to TARE using e.h. Ho or Y-beads to optimize cathether position. Such an optimization may provide better bead coverage of the intended target area. As US contrast agenst and TARE-beads differ about a factor 10 and more in diameter, a different biodistribution and flow pattern amy arise, which was sbject of the investigation. To this aim, the authors performed ex vivo experiments in a perfused pig liver model and compared US based contrast agent distrbution with MRI based quatification of Ho-beads.
The authors suggest that CE-US is a suitable tool to predict and optimize the TARE procedure, thus prviding improved outcome.
Overall the article is very well written with a detailed description fo all experimental details. The introduction puts the work into current state of the art and highlights how clinical practice could be improved. The analysis is well done and provides a comprehensive data set that substatiates the aims as well as the claims of the paper. Overall, I am absolutely supportive of publication of this article as this procedure once shown in clinical practice may improve treatment outcome. Also, the entire workflow should be easilty translatable into at least a clinical trial as now substatial new equipment or contrast agents are used, requiring extensive regulatory hurdles.
I also appreciate the very detailed discussion on limitations, which highlights all issues that need to be adressed e.g. in a clinical study to establish this procedure for routine use.
However, the most important aspect in my underwtanding is mentioned in the end, page 24 until end of the section.

“Finally, the use of a healthy ex-vivo perfused porcine liver to demonstrate DCE-US guided TARE limits the direct clinical translation of the prediction method. First, the absence of tumors results in different hemodynamic conditions, as microspheres lack a preferential deposition site after arterial injection. Second, the ex-vivo liver allowed direct fixation of the ultrasound transducer, enabling stable and consistent ultrasound imaging. In-vivo, however, factors such as the presence of surrounding organs and respiratory motion may pose additional challenges. Lastly, although anatomical and vascular structures are similar between porcine livers and human livers, differences in their overall shape may affect the generalizability of the findings in this study [67].

Indeed, all experiments were performed ex vivo on a healthy, perfused pig liver. Doing in vivo pig studies myself, I do understand the effort it takes to perform in vivo studies and also the necessity to start with ex-vivo experiments. A few aspects could however be discussed in more depth also taking existing literature into account.
1) How different is US imaging ex-vivo vs. in vivo? Especially for ultrasound imaging? Wouldn’t it be easily to simply test this? If for example the presence of the rib cage is a problem, then this would also hamper the translation into clinical practice.
2) New ultrasound approaches use particle tracking, which can compensate also for motion. Could this potentially be an alternative to simply follow the flow trajectories of CA with US.
3) The authors mention the different hemodynamics due to the presence of a tumor. This problem may change the distribution of particles and contrast agents due to their different size, as the particles are embolizing, while the US -CA are not. In the context of TACE, a very quick literature search showed me that contrast enhanced US has been compared to particle distribution in TACE procedures, which is a similar problem for this work.
Few aspects are mention on Page 23, “Previous studies have explored the use of contrast-enhanced ultrasound to assess the biodistribution in transarterial chemoembolization [44], [45], [46]….” . It is highlighted that only qualitative analysis was done to optimize the procedure.

I find it an important aspect to show that these “qualitative” approaches don’t yield the same outcome, or in other words, does the current approach really approve it?

A quick literature search shows more articles in this direction:
See for example:
doi: 10.4254/wjh.v10.i1.95
doi: 10.3390/diagnostics11020291

DOI: 10.21037/tcr.2020.04.17
Doi: 10.1007/s00261-021-03059-y
More article may exist, but I didn't do a systematic search.

Also, the publication 25 by the same first author contains on this aspect already some more details worth to expand here.

Could the authors expand the discussion and also the section of limitations taking above questions into account?

Nevertheless, after revision, this article should certainly be published.

Reviewer #3

(Remarks to the Author)
van der Hoek et al. present an interesting study on using catheter-based delivery of ultrasound contrast agents to predict TARE microsphere distribution. This is a clinically relevant problem and current approaches are suboptimal. The team demonstrates that CEUS predicts microsphere delivery in three ex vivo porcine livers, with confirmation determined using MRI of the homlmium spheres. Analysis is robust, the ex vivo model quite elegant, and the manuscript well written. Reults are very preliminary (n=3 with limited analysis), but given the novelty and stated goal of demonstrating feasibility I do not believe this to be a major weakness. I have only minor suggested edits below.

- Figure 1: I understand that this is the envisioned approach but was initially confused by the presence of tumor in the diagram. I would make this clearer in the legend.

- Some of the included material is likely not needed and the manuscript is quite long given the n=3. I would recommend incorporating the information in Table 1 into the text and likely removing Figures 2 and 4.

- Thinking of the TIC curves in eventual clinical practice, you would expect a delayed arrival of contrast agents after the initial first vascular pass given the blood is circulating. This presumably does not happen in your ex vivo setup due to filtering (?) but this should be clarified and discussed.

- 10% variability is described across acquisitions. It would be interesting to note if this was a gradual increase or decrease. I ask because authors state high MI imaging was used to clear contrast between injections, but some work as demonstrated alterations in perfusion resulting from bubble cavitation.

- A more detailed analysis is fluoro data would be helpful. Can contrast of the iodanted agents show similar results? This may be more practical in an actual tumor model but would be helpful to describe in more detail.

- The overall sample size is quite limited but it would still be nice to try and show some level of correlation across the entire dataset (similar to reference 25 from the group's in vitro work).

-Moving into in vivo or clinical trials, solely basing results on signal intensity may be challenging due to variations in attenuation. Has the group considered looking at perfusion or possibly tumor to tissue enahcnement ratios? This seems like it will be a challenge moving forward.

Reviewer #4

(Remarks to the Author)
I co-reviewed this manuscript with one of the reviewers who provided the listed reports. This is part of the Communications Medicine initiative to facilitate training in peer review and to provide appropriate recognition for Early Career Researchers who co-review manuscripts.<sup[A]

Version 1:

Reviewer comments:

Reviewer #1

(Remarks to the Author)
The authors have adequately responded to reviewer concerns.

Reviewer #2

(Remarks to the Author)

Reviewer #3

(Remarks to the Author)

The authors have responded to all prior concerns and I believe the manuscript is not suitable for publication.

Reviewer #4

(Remarks to the Author)
I co-reviewed this manuscript with one of the reviewers who provided the listed reports. This is part of the Communications Medicine initiative to facilitate training in peer review and to provide appropriate recognition for Early Career Researchers who co-review manuscripts.

Open Access This Peer Review File is licensed under a Creative Commons Attribution 4.0 International License, which permits use, sharing, adaptation, distribution and reproduction in any medium or format, as long as you give appropriate credit to the original author(s) and the source, provide a link to the Creative Commons license, and indicate if changes were made.
In cases where reviewers are anonymous, credit should be given to 'Anonymous Referee' and the source.
The images or other third party material in this Peer Review File are included in the article's Creative Commons license, unless indicated otherwise in a credit line to the material. If material is not included in the article's Creative Commons license and your intended use is not permitted by statutory regulation or exceeds the permitted use, you will need to obtain permission directly from the copyright holder.
To view a copy of this license, visit https://creativecommons.org/licenses/by/4.0/

This manuscript, *"Dynamic contrast-enhanced ultrasound predicts microsphere biodistribution in real-time during transarterial radioembolization,"* presents an interesting and technically strong idea. Using DCE-US for real-time guidance in TARE is innovative and addresses a real clinical gap. The work is promising, but in its current form it requires major revision before it could be considered for publication in *Communications Medicine*.

1- The main assumption of the paper (that microbubble distribution on DCE-US reliably reflects Y-90/Ho-166 microsphere deposition), is not fully convincing as currently presented. The Background section should more clearly acknowledge the physical and flow-related differences between microbubbles and microspheres, and frame this relationship as something being tested rather than treated as established.
2- Although the technical setup is impressive, the Methods section lacks a clear, predefined framework for evaluating how accurate the predictions really are. The very small sample size (n = 3), lack of power calculations, and absence of reproducibility testing make it hard to judge how robust or generalizable the findings are.
3- The Results are mostly descriptive. Much of the evidence relies on visual comparisons and qualitative interpretation of time–intensity curves and MRI maps. The manuscript would be much stronger if it included formal quantitative analyses (e.g., correlations, error metrics, confidence intervals) to support the claims of predictive accuracy.
4- A major concern is that the "ground truth" used for comparison (MRI R2* maps) could not be validated against a clinical standard such as Q-Suite. This creates a logical gap, since the method is being validated against a reference whose accuracy is itself uncertain within this study.
5- The Discussion at times feels overly optimistic about clinical translation. Phrases suggesting near-term clinical readiness are difficult to justify given that the work was done in an ex vivo, non-tumor, metabolically unstable porcine liver model. The conclusions should be more clearly framed as early proof-of-concept results.

Reviewer: 1

Comments to the Author

Summary:

This manuscript presents a proof-of-concept study investigating the use of dynamic contrast-enhanced ultrasound (DCE-US) for real-time prediction and optimization of microsphere biodistribution during transarterial radioembolization (TARE). Using ex-vivo perfused porcine livers, the authors injected ultrasound contrast microbubbles at various catheter positions and analyzed time-intensity curves (TICs) to optimize catheter placement. Holmium-165 microspheres were subsequently administered, and their distribution was assessed via MRI. Results demonstrated a strong correlation between microbubble and microsphere biodistribution, suggesting that DCE-US could provide intra-procedural feedback to improve targeting accuracy.

Major Strengths:

1) Innovative Concept and Clinical Relevance: The integration of DCE-US for real-time feedback during TARE addresses a critical limitation of current scout-based planning methods, which suffer from time delays and replication errors.

2) Proof-of-Concept Validation: The study demonstrates a strong correlation between microbubble and microsphere biodistribution (e.g., Case 2: 99.2% vs. 98%), supporting the feasibility of the proposed approach.

3) Practicality and Feasibility: The method leverages widely available ultrasound systems and clinically approved contrast agents, making it potentially easy to implement in hybrid operating rooms without major infrastructure changes.

We thank the reviewer for highlighting and acknowledging these strengths of our study.

Major Weaknesses:

1) Limited Clinical Translation: Experiments were performed on healthy ex-vivo porcine livers, which do not replicate tumor vascularity or in-vivo physiological conditions. This significantly limits the direct applicability of findings to human patients.

We acknowledge this limitation and note that the present study warrants further clinical investigation to establish the predictive capability of DCEUS-guided TARE in vivo. However, preferential uptake is expected to influence distribution rather than the predictive capability of microbubbles (i.e. microbubbles and microspheres are still expected to follow similar trajectories in tumorous livers):

Page **29**, line **21-23**: 'Finally, the use of a healthy ex-vivo perfused porcine liver to demonstrate DCE-US guided TARE limits the direct clinical translation of the prediction method. First, the absence of tumors results in different hemodynamic conditions, as microspheres lack a preferential deposition site after arterial injection.'

2) Restricted Scope of Optimization: The study only evaluated catheter position changes using standard injection parameters. Other clinically relevant factors—such as injection velocity, pulsatile flow, or alternative catheter designs—were not explored.

The scope of this paper is a proof-of-concept demonstration. The influence of TARE parameters on microsphere distribution patterns has already been well established in numerous in vivo, ex vivo, and in vitro studies, as summarized in the systematic literature review by Snoeijink et al., which has now been added to the references ([71], Page **29**, line **18**). Although investigating these parameters in the context of the demonstrated DCE-US approach would be of interest for future work, this was beyond the scope of the present study.

Page **29**, line **15-20**: 'Another limitation of the study is that microbubble-based prediction was evaluated only for catheter position changes, using standard injection parameters. Alternative injection strategies, such as pulsatile injections, increased injection velocities, or the use of different catheters, may influence microbubble and microsphere biodistribution patterns [71], potentially improving or compromising the accuracy of the prediction method. On the other hand, the real-time feedback provided by DCE-US could prove to be ideal to assess these parameters, which may instead further improve optimization of the catheter configuration.'

3) Two-Dimensional Imaging Limitation: DCE-US analysis was restricted to a fixed transducer position, providing only a 2D view of microbubble distribution. This limited spatial coverage and contributed to off-target deposition in Case 1.

We agree with the reviewer that the DCE-US analysis in this study was restricted by the fixed transducer positions. In the present experimental setup, the transducer was fixed intentionally to focus on the comparison of the different catheter positions. However, the transducer should also be moved after creating a TIC to scan for depositions around the region of interest.

Page **25**, line **17-23**: 'Finally, DCE-US did not provide a three-dimensional overview of the expected microsphere biodistribution, as the region of analysis is restricted to the two-dimensional imaging plane. In this study, the assessment of different catheter positions relied on comparing their resulting peak intensities, which required transducer fixation and provided limited spatial coverage. To address this limitation, the transducer could be moved during an additional microbubble injection to scan a wider area and help identify the biodistribution boundaries of the candidate catheter configuration.'

4) Perfusion Model Instability: Blood gas analysis revealed suboptimal pH and oxygen saturation during perfusion, which could influence vascular dynamics and microbubble/microsphere behavior, introducing uncertainty in experimental validity.

Although these factors may influence vascular dynamics, previous MRI-based perfusion measurements in this model (source [27] in the manuscript) demonstrated uniform perfusion despite suboptimal oxygen saturation and pH levels. This suggests that the overall vascular flow conditions were maintained. Therefore, these conditions are not expected to have a substantial effect on the behavior or distribution of microbubbles and microspheres.

Minor Comments:

1) Figures are well-illustrated but could benefit from clearer labeling of transducer positions and catheter paths for reproducibility.

We have updated the figures in the manuscript. Moreover, we have added the x-ray images displaying each catheter position and the ultrasound transducer positions to the Supplementary Materials (1), which are now referred to in the text:

Page **16**, line **22-23**: 'The fluoroscopy images that have been used for determining the exact catheter and ultrasound transducer positions can be found in **Supplemental Materials 1 (Figures 1 & 2)**.'

Page **17**, line **18-19**: 'Representative fluoroscopy images for each catheter position are provided in **Supplemental Material 1 (Figures 3 to 6)**.'

Page **19**, line **3-4**: 'Fluoroscopy images for each catheter position are provided in **Supplemental Material 1 (Figures 7 to 9)**.'

Page **21**, line **16**: 'Fluoroscopy images for each catheter position are provided in **Supplemental Material 1 (Figures 10 to 15)**.'

2) The discussion section is comprehensive but could be condensed for clarity, focusing on clinical implications and future directions.

While we agree that improving clarity and maintaining a clear focus on the clinical implications and future directions is important for the discussion section, other reviewers and the Editor requested additional context in relation to the existing literature. Specifically, they suggested further discussion of the differences between ex vivo and in vivo ultrasound imaging (including limitations such as the rib cage), the potential influence of motion artefacts, and the differences in embolization behavior between microspheres and microbubbles. We have incorporated these aspects (Page **29/30**, line **25-9**) into the discussion while carefully revising the section to maintain clarity and readability.

3) Consider adding quantitative correlation metrics (e.g., Pearson correlation coefficients) between TIC peak intensities and MRI-derived microsphere distributions for stronger statistical support.

A direct quantitative correlation between TIC peak intensities and the MRI-derived microsphere distributions using correlation coefficients, e.g., Pearson correlation coefficient, is not feasible, as the domains of the two datasets are different (temporal vs. spatial). Moreover, due to the intrinsic features of the embolization, microspheres can only be injected once. Therefore, optimization using TICs is essential to achieve a focused distribution, however, this also prevents comparison of each TIC with a subsequent resulting microsphere distribution. Additional quantification based on the analyzed TICs was performed to gain further insight into the resulting distributions from different injection positions, as shown in Tables 3, 4, and 5.

Reviewer: 2

I received the manuscript entitled "Dynamic contrast-enhanced ultrasound predicts microsphere biodistribution in real-time during transarterial radioembolization" authored by van der Hoek et al. for review.

Th article described the use of contrast enhance ultrasound scans prior to prior to TARE using e.g. Ho or Y-beads to optimize catheter position. Such an optimization may provide better bead coverage of the intended target area. As US contrast agents and TARE-beads differ about a factor 10 and more in diameter, a different biodistribution and flow pattern may arise, which was subject of the investigation. To this aim, the authors performed ex vivo experiments in a perfused pig liver model and compared US based contrast agent distribution with MRI based quantification of Ho-beads.

The authors suggest that CE-US is a suitable tool to predict and optimize the TARE procedure, thus providing improved outcome.

Overall the article is very well written with a detailed description for all experimental details. The introduction puts the work into current state of the art and highlights how clinical practice could be improved. The analysis is well done and provides a comprehensive data set that substantiates the aims as well as the claims of the paper. Overall, I am absolutely supportive of publication of this article as this procedure once shown in clinical practice may improve treatment outcome. Also, the entire workflow should be easily translatable into at least a clinical trial as no substantial new equipment or contrast agents are used, requiring extensive regulatory hurdles.

I also appreciate the very detailed discussion on limitations, which highlights all issues that need to be addressed e.g. in a clinical study to establish this procedure for routine use.

We would like to thank the reviewer for their assessment of the manuscript and for the positive remarks.

However, the most important aspect in my understanding is mentioned in the end, page 24 until end of the section. "Finally, the use of a healthy ex-vivo perfused porcine liver to demonstrate DCE-US guided TARE limits the direct clinical translation of the prediction method. First, the absence of tumors results in different hemodynamic conditions, as microspheres lack a preferential deposition site after arterial injection. Second, the ex-vivo liver allowed direct fixation of the ultrasound transducer, enabling stable and consistent ultrasound imaging. In-vivo, however, factors such as the presence of surrounding organs and respiratory motion may pose additional challenges. Lastly, although anatomical and vascular structures are similar between porcine livers and human livers, differences in their overall shape may affect the generalizability of the findings in this study [67]."

Indeed, all experiments were performed ex vivo on a healthy, perfused pig liver. Doing in vivo pig studies myself, I do understand the effort it takes to perform in vivo studies and also the necessity to start with ex-vivo experiments. A few aspects could however be discussed in more depth also taking existing literature into account.

1) How different is US imaging ex-vivo vs. in vivo? Especially for ultrasound imaging? Wouldn't it be easily to simply test this? If for example the presence of the rib cage is a problem, then this would also hamper the translation into clinical practice.

There are indeed differences between ex vivo and in vivo ultrasound imaging, for example due to acoustic shadowing from the rib cage and bowel gases. However, several practical strategies are available that sonographers can use to reliably image the liver, and they are therefore not expected to substantially hinder clinical translation. We elaborated on this in the last paragraph of the revised discussion:

Page **29/30**, line **26-1**: 'Nevertheless, these difficulties can largely be mitigated through current ultrasound scanning protocols, in which imaging from an alternative angle or under breath-hold helps

prevent acoustic shadowing (e.g. by the rib cage), as well as through appropriate patient preparation, such as fasting to reduce the effects of bowel gas [72].'

2) New ultrasound approaches use particle tracking, which can compensate also for motion. Could this potentially be an alternative to simply follow the flow trajectories of CA with US.

Our perfusion approach has shown successful correlation with microsphere distributions with changing catheter positions, which was the primary aim of this study. We are fully aware of the novel ultrasound techniques such as ultrafast plane wave imaging that allow for reconstructing flow velocity vectors and maps [Mirgolbabaee et al., https://pubmed.ncbi.nlm.nih.gov/38149463/], even super-resolved [Blanken et al., https://pubmed.ncbi.nlm.nih.gov/35404813/], by tracking particle trajectories, and that these can compensate for motion artefacts. Hepatic blood flow however is complex, multiscale (large hepatic vessels vs. microvasculature) and operates at multiple timescales, which would require a significant effort in developing a valuable representation of flow in the vast liver vasculature if we are interested in catheter position alone. We do think particle tracking and super-resolved ultrasound localization microscopy could fit in is in the evaluation of the embolization degree of the microvasculature. This will be the subject of future work.

3) The authors mention the different hemodynamics due to the presence of a tumor. This problem may change the distribution of particles and contrast agents due to their different size, as the particles are embolizing, while the US -CA are not. In the context of TACE, a very quick literature search showed me that contrast enhanced US has been compared to particle distribution in TACE procedures, which is a similar problem for this work. Few aspects are mention on Page 23, "Previous studies have explored the use of contrast-enhanced ultrasound to assess the biodistribution in transarterial chemoembolization [44], [45], [46]…." . It is highlighted that only qualitative analysis was done to optimize the procedure.

I find it an important aspect to show that these "qualitative" approaches don't yield the same outcome, or in other words, does the current approach really approve it?

A quick literature search shows more articles in this direction:

See for example:

doi: 10.4254/wjh.v10.i1.95
doi: 10.3390/diagnostics11020291
DOI: 10.21037/tcr.2020.04.17
Doi: 10.1007/s00261-021-03059-y

More article may exist, but I didn't do a systematic search. Also, the publication 25 by the same first author contains on this aspect already some more details worth to expand here.

We agree that we can elaborate more on the added value of quantitative analysis with respect to the qualitative approaches in the cited articles. We have added the following text to our discussion:

Page **26**, line **20-27**: 'Quantitative analysis provides greater control in treatment planning by enabling direct comparison of different catheter configurations. Particularly in cases where contrast enhancement is observed at multiple locations and visual comparison is non-trivial (e.g., Case 3, **Error! Reference source not found.**), quantification supports a more objective and substantiated choice for the optimal catheter configuration. Furthermore, quantitative evaluation facilitates assessment of tumor perfusion, contributing to more comprehensive tumor localization and potentially enabling detection of lesions not evident on other imaging modalities [31], [47]. To our knowledge, this study is the first to employ ultrasound-based analysis to quantitatively optimize the biodistribution in locoregional therapy.'

Could the authors expand the discussion and also the section of limitations taking above questions into account? Nevertheless, after revision, this article should certainly be published.

The discussion has been expanded accordingly (Page **26**, line **20-27**), and we appreciate the support of the reviewer for the publication of our work.

Reviewer: 3

van der Hoek et al. present an interesting study on using catheter-based delivery of ultrasound contrast agents to predict TARE microsphere distribution. This is a clinically relevant problem and current approaches are suboptimal. The team demonstrates that CEUS predicts microsphere delivery in three ex vivo porcine livers, with confirmation determined using MRI of the holmium spheres. Analysis is robust, the ex vivo model quite elegant, and the manuscript well written. Results are very preliminary (n=3 with limited analysis), but given the novelty and stated goal of demonstrating feasibility I do not believe this to be a major weakness. I have only minor suggested edits below.

We thank the reviewer for the positive appraisal of our work

Figure 1: I understand that this is the envisioned approach but was initially confused by the presence of tumor in the diagram. I would make this clearer in the legend.

We agree that the distinction between the clinically envisioned approach and the experimental model using healthy livers was not clear. The caption of Fig. 1 has now been changed to emphasize this difference:

Page **6**, line **13-18**: 'Figure 1: Overview of the envisioned clinical dynamic contrast-enhanced ultrasound-guided radioembolization procedure. A) After identification of a tumor (or target site in this study), the ultrasound transducer is focused on this region and B) the catheter is inserted under fluoroscopy guidance to several locations that are deemed suitable for targeting the tumor. C) Microbubbles are injected at these catheter positions, D) which are directly quantified to provide real-time feedback of the suitability of a catheter position by the resulting peak of the time intensity curve (TIC). The optimum catheter positions can then be chosen for the injection of radioactive ^{166}Ho microspheres.'

Some of the included material is likely not needed and the manuscript is quite long given the n=3. I would recommend incorporating the information in Table 1 into the text and likely removing Figures 2 and 4.

We thank the reviewer for this suggestion. The LiverTwin machine perfusion system is an experimental setup that has been developed specifically for these experiments and has not been described previously in the literature. For this reason, we believe that including Table 1 and Figures 2 and 4, are required to document the experimental setup. We therefore would prefer to retain these parts for clarity and reproducibility of the model.

Thinking of the TIC curves in eventual clinical practice, you would expect a delayed arrival of contrast agents after the initial first vascular pass given the blood is circulating. This presumably does not happen in your ex vivo setup due to filtering (?) but this should be clarified and discussed.

We thank the reviewer for this comments and agree that it should be discussed. We have added the following limitation to our study:

Page **30**, line **4-9**: 'Third, microbubble recirculation was not observed in the ex vivo perfused liver. This is likely due to microbubble destruction in the oxygenators and buoyancy toward the free interface of the reservoir, preventing re-entry into the perfusion circuit. In vivo, however, microbubbles recirculate and can produce delayed secondary peaks after approximately 60 seconds [69], potentially affecting TIC-derived parameters that rely on the wash-out phase and requiring correction of TICs when comparing curves for rapid catheter position optimization in clinical practice.'

10% variability is described across acquisitions. It would be interesting to note if this was a gradual increase or decrease. I ask because authors state high MI imaging was used to clear contrast between injections, but some work as demonstrated alterations in perfusion resulting from bubble cavitation.

The observed variability was not gradual over successive acquisitions. It most likely reflects small differences in the injected bolus, as the use of small volume injections introduces some uncertainty in

the exact number of administered microbubbles. While ultrasound bursts with high MI were used to clear contrast, alterations to perfusion due to cavitation were not observed in the TICs.

A more detailed analysis is fluoro data would be helpful. Can contrast of the iodanted agents show similar results? This may be more practical in an actual tumor model but would be helpful to describe in more detail.

Although iodinated contrast injection is currently used in clinical practice for similar purposes, it has been shown to yield inferior functional information compared to contrast-enhanced ultrasound (CEUS). The following has been added to the discussion:

Page **28**, line **5-7**: 'However, contrast-enhanced ultrasound (CEUS) has already been shown in transarterial chemoembolization to alter treatment planning by identifying superselective feeding arteries that were subsequently targeted instead of those selected based on angiography [59], [60].'

Page **28**, line **11-14**: 'Finally, CBCT provides only static images, which limits its utility in predicting depositions in non-tumor tissue and in tumors with different vascularity [63], whereas CEUS enhancement patterns have been reported to outperform CBCT-derived patterns in delineating tumor boundaries to enhance treatment planning [64].'

The overall sample size is quite limited but it would still be nice to try and show some level of correlation across the entire dataset (similar to reference 25 from the group's in vitro work).

The aim of this study was to demonstrate proof-of-concept for the feasibility of intra-procedural feedback using DCE-US for catheter optimization. The experiments were intentionally designed to produce different microsphere distributions. For example, in one case the distribution was largely confined to a single lobe, whereas in the two other cases distributions were observed across multiple lobes. While this variability limits quantitative comparisons across experiments, we have added additional quantification within individual cases. Ratios were defined based on the first analyzed catheter position for both the target and reference location (T_P/T_1 and R_P/R_1) to define improvement, and target to reference signal was defined by T_P/R_P for direct comparison of the distributions over the two analyzed regions. The additional quantification can now be found in the methods and results sections, including tables 3, 4 and 5 in the results section:

Page **16**, line **9-14**: 'Three ex vivo livers were analyzed on a case-by-case basis. For each case, all generated TICs were normalized to the maximum peak intensity measured at the target position within that case. For each injection location, mean peak intensities were calculated from three measurements, and the corresponding standard deviations were reported (±σ). In addition, two ratios were computed using the mean peak intensities: the relative signal improvement with respect to the first injection location, calculated separately for the target and reference positions (T_P/T_1 and RP/R_1), and the target-to-reference signal ratio (T_P/R_P).'

Page **19**, line **6-11**: 'From position P1 to P2, peak intensity at the target area increased by $T_2/T_1 = 1.5$. For positions P2 and P3, the signal in the reference area dropped to $R_2/R_1 = 0.04$ and $R_3/R_1 = 0.03$, indicating catheter advancement beyond the feeding artery of the reference area. From position P2 to P3, signal intensity decreased noticeably due to only targeting a part of the ROI, evidenced by the heterogeneous biodistribution of microbubbles resulting from injection at position P3, as shown in Figure 9. While a higher target-to-reference signal ratio was found for position P3 ($T_2/R_2 = 48$, $T_3/R_3 = 58$), microspheres were injected at position P2 for better coverage of the target region.'

Page **21/22**, line **19-3**: 'From the initial position P1 to the final position P6, peak intensity at the target area increased by $T6/T1 = 3.0$, while the peak intensity at the reference area decreased to $R_6/R_1 = 0.6$. For positions P1 to P5, the signal intensity at the reference area consistently exceeded that of the target area. Small displacements of approximately 1 mm resulted in significant differences between target and reference signals. Microspheres were injected at position P6, where the target-to-reference

signal first exceeded 1 (T_6/R_6 = 3.4), resulting in a microsphere deposition in the target area and the reference area (70.2% vs. 29.8%), based on analysis regions defined similarly to the previous case.'

Moving into in vivo or clinical trials, solely basing results on signal intensity may be challenging due to variations in attenuation. Has the group considered looking at perfusion or possibly tumor to tissue enhancement ratios? This seems like it will be a challenge moving forward.

We agree that this represents one of the technical challenges for clinical translation. Several approaches reported in the literature may help mitigate these issues, including defining tumor-specific regions of interest (ROIs) based on CEUS-guided identification and employing more robust time–intensity curve (TIC) analyses, such as calculating the area under the curve rather than relying solely on peak intensity. We have updated the limitations section to reflect this consideration.

Page **30**, line **1-4**: 'It should also be noted that the deeper location of the liver and the presence of tumors in vivo can introduce variations in ultrasound attenuation, for which several strategies exist to enhance the robustness of the demonstrated DCE-US approach, such as defining tumor-specific ROIs or using alternative TIC-derived parameters, including time to peak or area under the curve [73].'

Reviewer #4:

I co-reviewed this manuscript with one of the reviewers who provided the listed reports. This is part of the Communications Medicine initiative to facilitate training in peer review and to provide appropriate recognition for Early Career Researchers who co-review manuscripts.

This manuscript, “Dynamic contrast-enhanced ultrasound predicts microsphere biodistribution in real-time during transarterial radioembolization,” presents an interesting and technically strong idea. Using DCE-US for real-time guidance in TARE is innovative and addresses a real clinical gap. The work is promising, but in its current form it requires major revision before it could be considered for publication in Communications Medicine.

1 - The main assumption of the paper (that microbubble distribution on DCE-US reliably reflects Y-90/Ho-166 microsphere deposition), is not fully convincing as currently presented. The Background section should more clearly acknowledge the physical and flow-related differences between microbubbles and microspheres, and frame this relationship as something being tested rather than treated as established.

The differences between microbubbles and microspheres have indeed been described in detail in the in vitro study conducted by our group [25]. In our revised manuscript, we have highlighted embolization as the main difference in the introduction and emphasized that the correlation should be established in a realistic perfused liver model:

Page **5**, lines **10-16**: ‘Given that these microbubbles are sufficiently small to pass through the capillary bed (mean diameter of 2.5 µm, with 90% of all microbubbles smaller than 8 µm [26]), unlike embolizing microspheres, sequential microbubble injections can be used to iteratively optimize the catheter configuration for improved tumor targeting during TARE. The resulting microbubble biodistribution can then be quantified using dynamic contrast-enhanced ultrasound (DCE-US), thereby establishing what we term the DCE-US guided TARE approach. However, it remains to be shown whether microbubbles can accurately predict the microsphere biodistribution in a realistic perfused liver model.’

The final paragraph of the introduction was revised to clearly indicate that this study tests the correlation between microbubbles and microspheres:

Page 5, line **17-22**: ‘This study presents a proof-of-concept for DCE-US guided TARE in healthy ex-vivo machine-perfused porcine livers. We show that real-time DCE-US feedback can be used to optimize catheter positioning for targeting specific regions. Microsphere injections after optimization produce depositions that correlate with the DCE-US predictions, demonstrating that microbubbles can accurately predict the microsphere biodistribution. These results suggest that DCE-US could be a promising tool to improve treatment planning for TARE, although the findings are preliminary and clinical feasibility remains to be established.’

2 - Although the technical setup is impressive, the Methods section lacks a clear, predefined framework for evaluating how accurate the predictions really are. The very small sample size (n = 3), lack of power calculations, and absence of reproducibility testing make it hard to judge how robust or generalizable the findings are.

This study is indeed presented as a proof-of-concept, focusing on the development and demonstration of the approach. We agree that the accuracy of the prediction method should be further investigated, however, we do believe that a solid foundation is given in this study and that we could turn towards a (pre-)clinical study to determine the clinical feasibility and accuracy of this method. Several changes in the revised manuscript were made to better frame the study:

Page **27**, line **12-14**: ‘The DCE-US approach shows potential to improve TARE treatment planning, building on the established safety and efficacy of contrast microbubbles, combined with the advantages of ultrasound imaging in this setting, i.e. real-time and portable.’

Page **28**, line **3-4**: 'First, current results indicate that ultrasound contrast microbubbles can predict the microsphere biodistribution, a relationship that has yet to be established for iodinated contrast agents used in CBCT.'

Page **30**, line **16-18**: 'These results demonstrate that DCE-US guided TARE can enhance microsphere targeting and hold promise for improved treatment outcomes, warranting further clinical investigation.'

3 - The Results are mostly descriptive. Much of the evidence relies on visual comparisons and qualitative interpretation of time–intensity curves and MRI maps. The manuscript would be much stronger if it included formal quantitative analyses (e.g., correlations, error metrics, confidence intervals) to support the claims of predictive accuracy.

We thank the reviewer for this comment. As discussed earlier, the aim of this study was to demonstrate a proof-of-concept of the feasibility of DCE-US for catheter optimization. Future studies with larger sample sizes and more standardized acquisition conditions will enable more extensive statistical analyses. However, we have added additional quantification for each individual case. Ratios were defined based on the first analyzed catheter position for both the target and reference location (T_P/T_1 and R_P/R_1) to define improvement, and target to reference signal was defined by T_P/R_P for direct comparison of the distributions over the two analyzed regions. The additional quantification can now be found in the methods and results sections, including tables 3, 4 and 5 in the results section:

Page **16**, line **9-14**: 'Three ex vivo livers were analyzed on a case-by-case basis. For each case, all generated TICs were normalized to the maximum peak intensity measured at the target position within that case. For each injection location, mean peak intensities were calculated from three measurements, and the corresponding standard deviations were reported (±σ). In addition, two ratios were computed using the mean peak intensities: the relative signal improvement with respect to the first injection location, calculated separately for the target and reference positions (T_P/T_1 and RP/R_1), and the target-to-reference signal ratio (T_P/R_P).'

Page **19**, line **5-11**: 'From position P1 to P2, peak intensity at the target area increased by $T_2/T_1 = 1.5$. For positions P2 and P3, the signal in the reference area dropped to $R_2/R_1 = 0.04$ and $R_3/R_1 = 0.03$, indicating catheter advancement beyond the feeding artery of the reference area. From position P2 to P3, signal intensity decreased noticeably due to only targeting a part of the ROI, evidenced by the heterogeneous biodistribution of microbubbles resulting from injection at position P3, as shown in Figure 9. While a higher target-to-reference signal ratio was found for position P3 ($T_2/R_2 = 48$, $T_3/R_3 = 58$), microspheres were injected at position P2 for better coverage of the target region.'

Page **21/22**, line **19-3**: 'From the initial position P1 to the final position P6, peak intensity at the target area increased by $T_6/T_1 = 3.0$, while the peak intensity at the reference area decreased to $R_6/R_1 = 0.6$. For positions P1 to P5, the signal intensity at the reference area consistently exceeded that of the target area. Small displacements of approximately 1 mm resulted in significant differences between target and reference signals. Microspheres were injected at position P6, where the target-to-reference signal first exceeded 1 ($T_6/R_6 = 3.4$), resulting in a microsphere deposition in the target area and the reference area (70.2% vs. 29.8%), based on analysis regions defined similarly to the previous case.'

4 - A major concern is that the "ground truth" used for comparison (MRI R2* maps) could not be validated against a clinical standard such as Q-Suite. This creates a logical gap, since the method is being validated against a reference whose accuracy is itself uncertain within this study.

We thank the reviewer for raising this point. The MRI R2* maps are not intended as a formal "ground truth", but rather as a visualization of the relative microsphere distribution. Moreover, the analysis method is based on the same physical principles used in Q-Suite. Hence, the accuracy of the microsphere depositions are not uncertain, and they are clearly visible on the raw MRI MGRE images,

providing direct qualitative confirmation of the distribution. As this part was unclear in the original text, we have now rephrased as follows:

Page **26**, line **1-3**: 'While the determined relative microsphere distributions visually corresponded to the T2*-weighted multi-gradient MRI scans (see **Supplemental Materials 2**), it should be considered an approximate representation rather than an exact measurement.'

5 - The Discussion at times feels overly optimistic about clinical translation. Phrases suggesting near-term clinical readiness are difficult to justify given that the work was done in an ex vivo, non-tumor, metabolically unstable porcine liver model. The conclusions should be more clearly framed as early proof-of-concept results.

We agree with the reviewer that the discussion was probably too optimistic regarding clinical translation. The study has now been framed as a proof-of-concept demonstration of the concept, and proposed workflow in an ex vivo machine-perfused liver model, rather than its immediate clinical applicability. Kindly see our revised formulations as addressed in our response to remark 2 of this reviewer.

We thank the reviewers again for their critical review that helped improve the quality of the manuscript.
