# Supplementary material for: Dynamic contrast-enhanced ultrasound predicts microsphere biodistribution in real-time during transarterial radioembolization
Source: Commun Med (Lond). 2026 Jun 30;6:546. doi: 10.1038/s43856-026-01760-x (PMC13683390; doi:10.1038/s43856-026-01760-x)
Supplement: Supplementary file 2 [file 43856_2026_1760_MOESM2_ESM.pdf]

Supplemental Materials DCE-US guided TARE proof-of-concept

1. LiverTwin machine perfusion platform

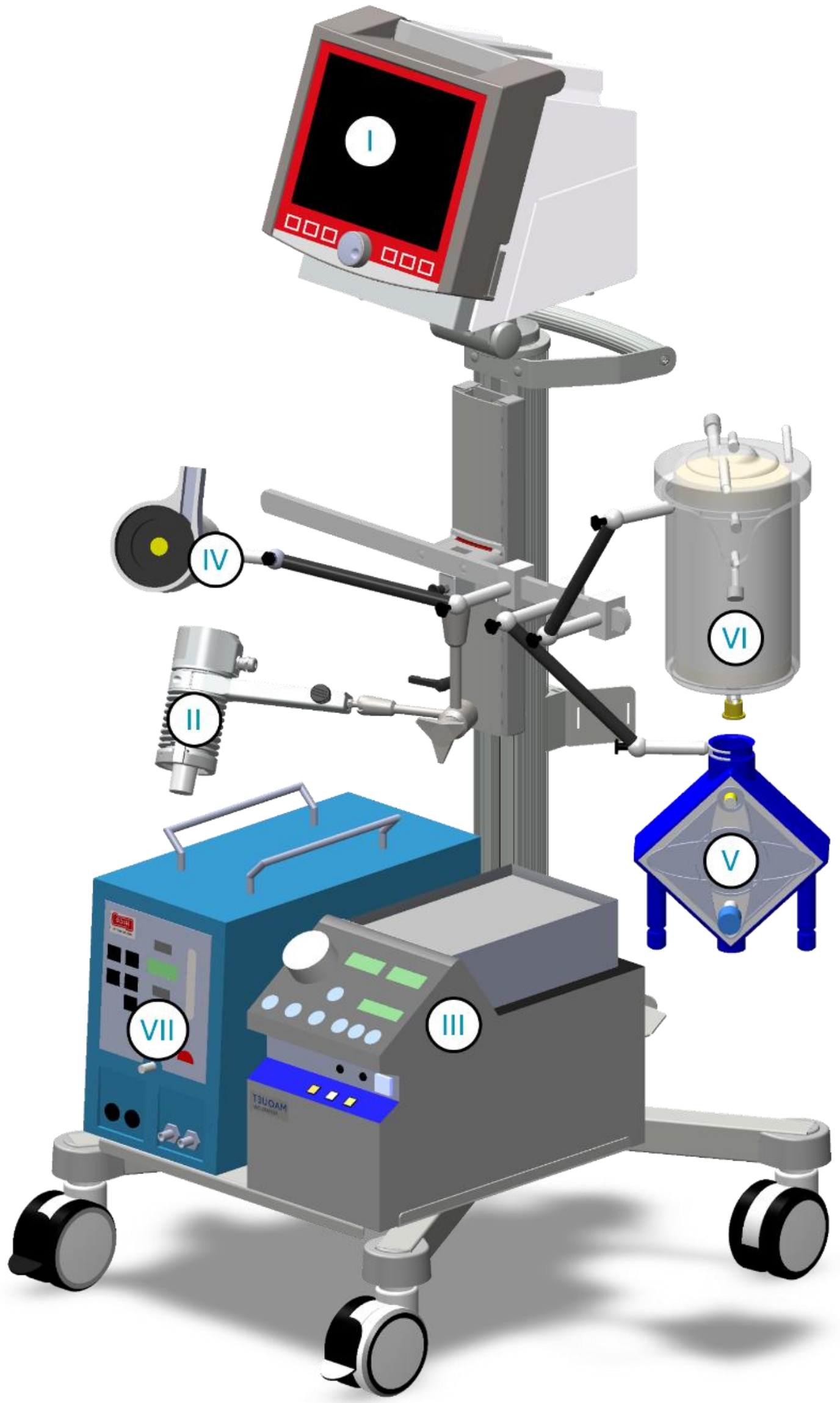

Figure S1: The LiverTwin machine perfusion platform. Multiple components were incorporated in the system to regulate the blood supply to the hepatic arterial (HA) and portal venous (PV) side of the liver, including pump consoles (I (HA), III (PV)), pump drivers for centrifugal pump heads (II (HA), IV (PV)), hollow-fiber oxygenators with integrated heat exchangers (V (HA), VI (PV)), and a heater-cooler system (VII (HA & PV)). The figure was created using SolidWorks® Education Edition (Dassault Systèmes).

2. Fluoroscopy images for determining the catheter position

Real-time DCE-US prediction example

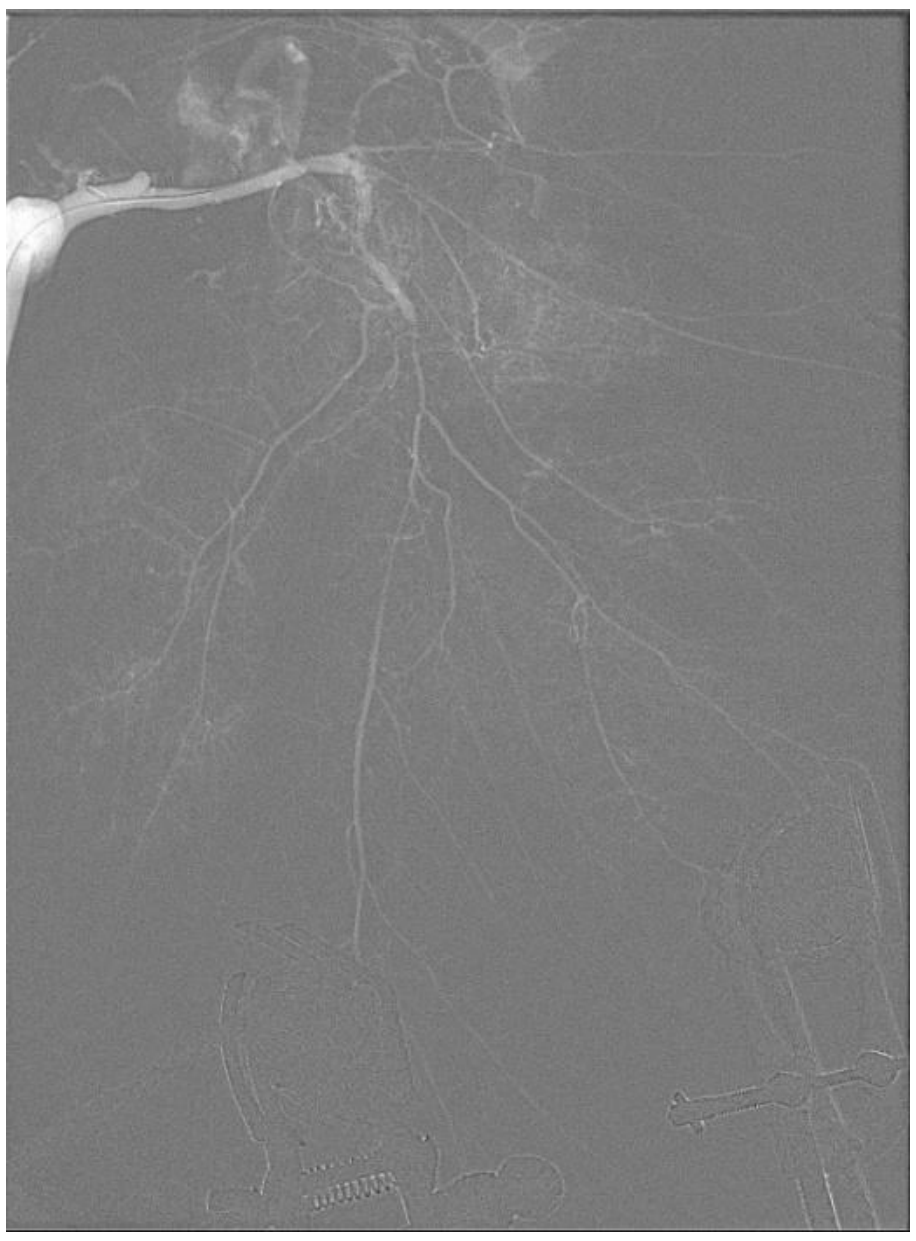

Figure S2: Fluoroscopy roadmap of catheter position P1 of the real-time DCE-US prediction example.

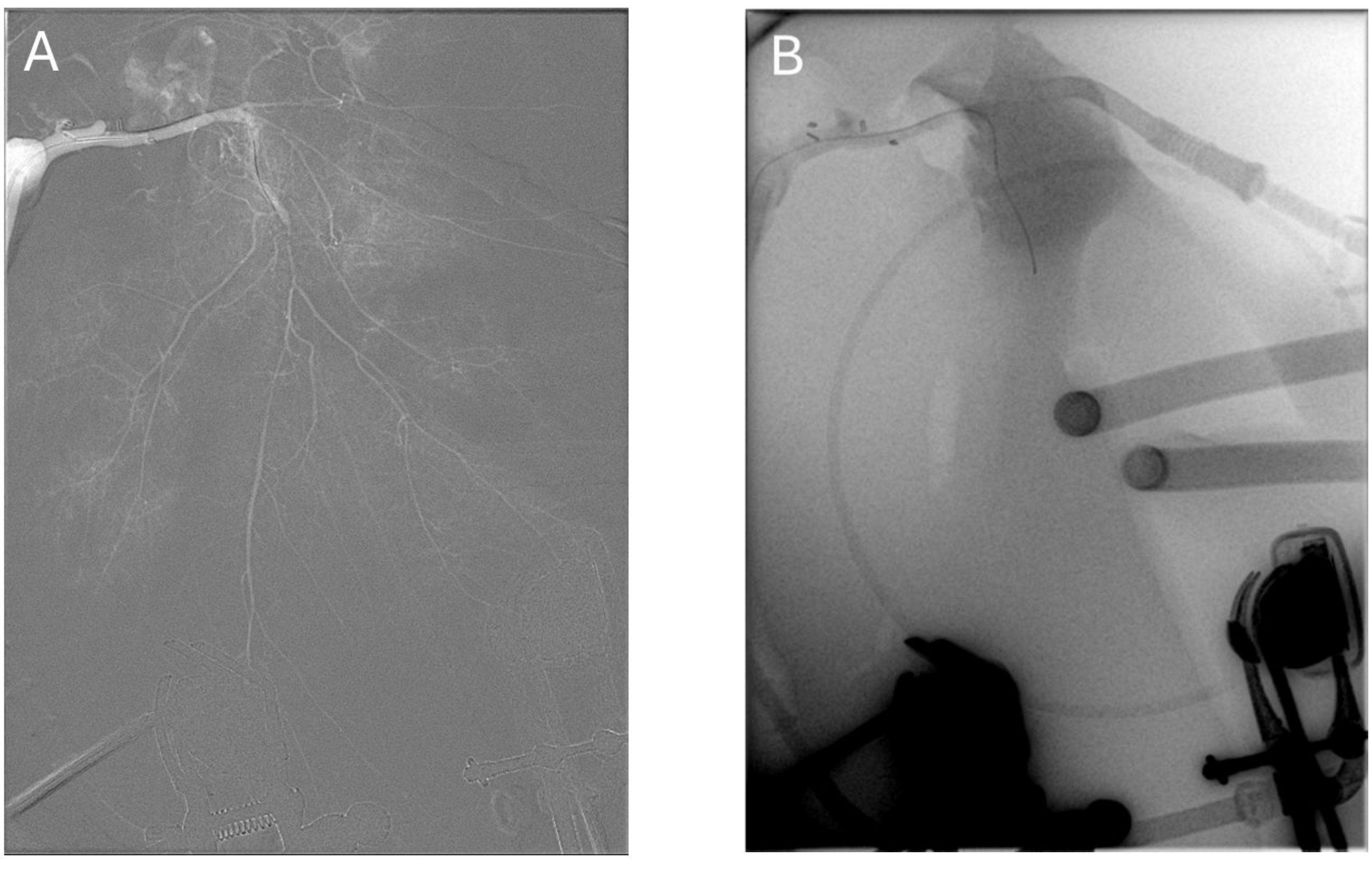

Figure S3: (A) Fluoroscopy roadmap of catheter position P2 of the real-time DCE-US prediction example, and (B) the isolated catheter including the clamps with ultrasound transducers in the x-ray image.

Case 1

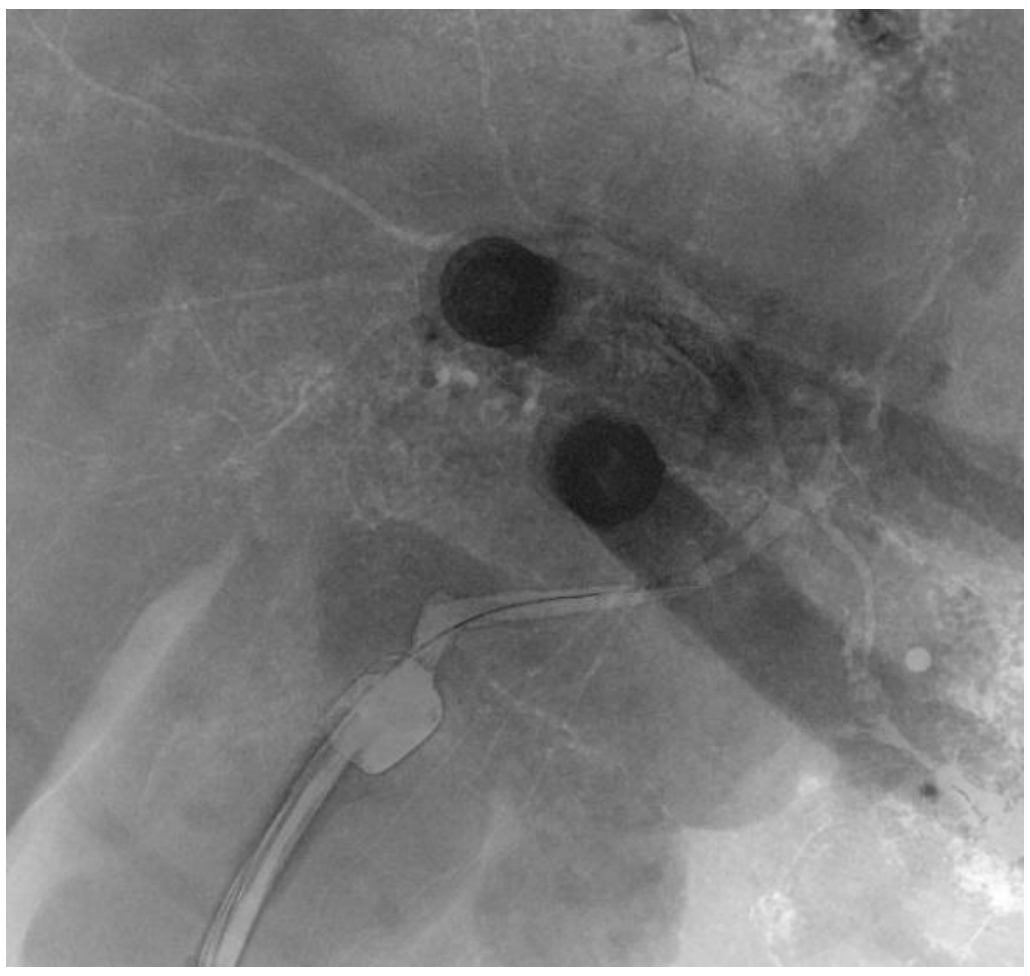

Figure S4: Fluoroscopy roadmap of catheter position P1 of Case 1.

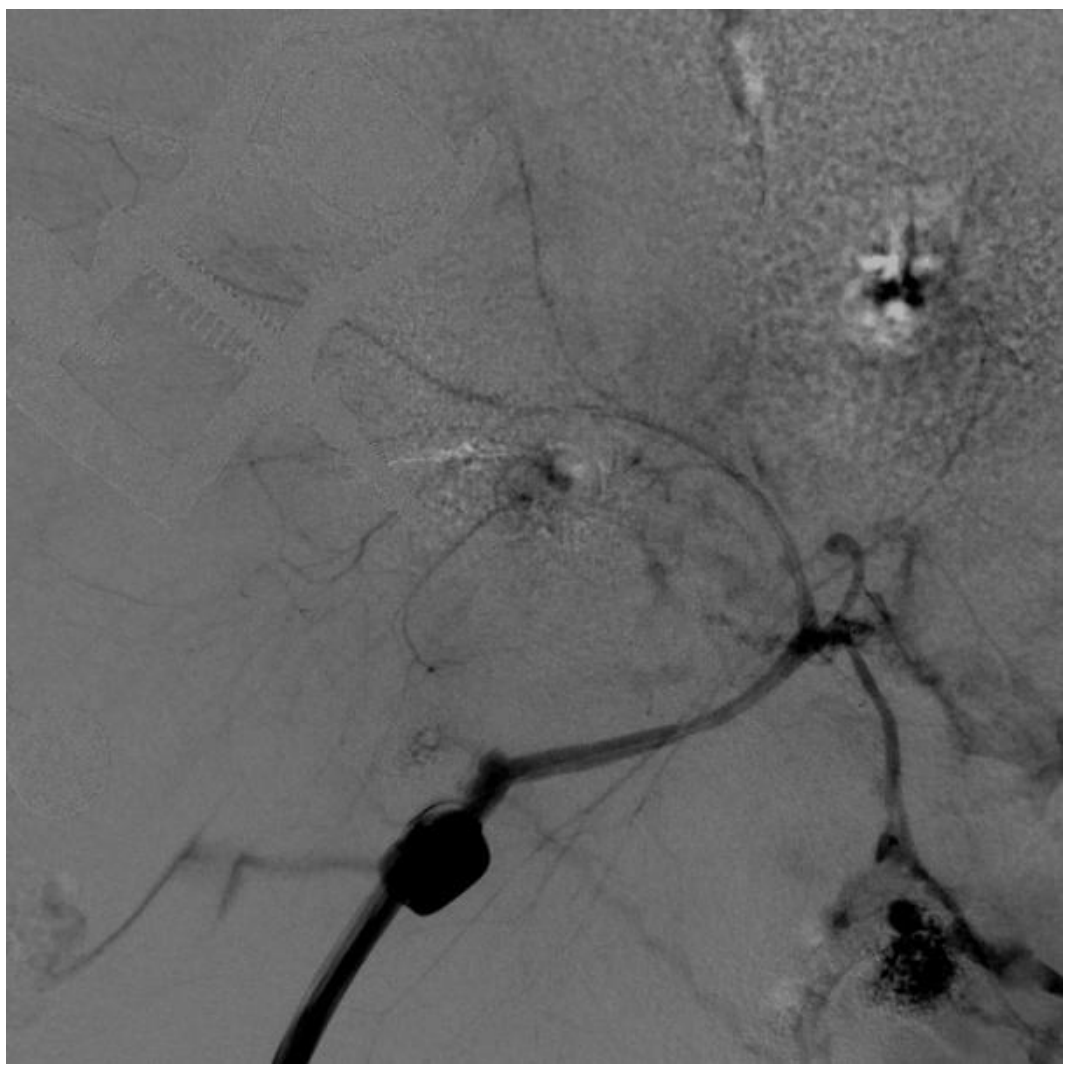

Figure S5: Digital subtraction angiography of catheter position P2 of Case 1. Note that the clamp with the ultrasound transducer is located in the upper left part of the angiography.

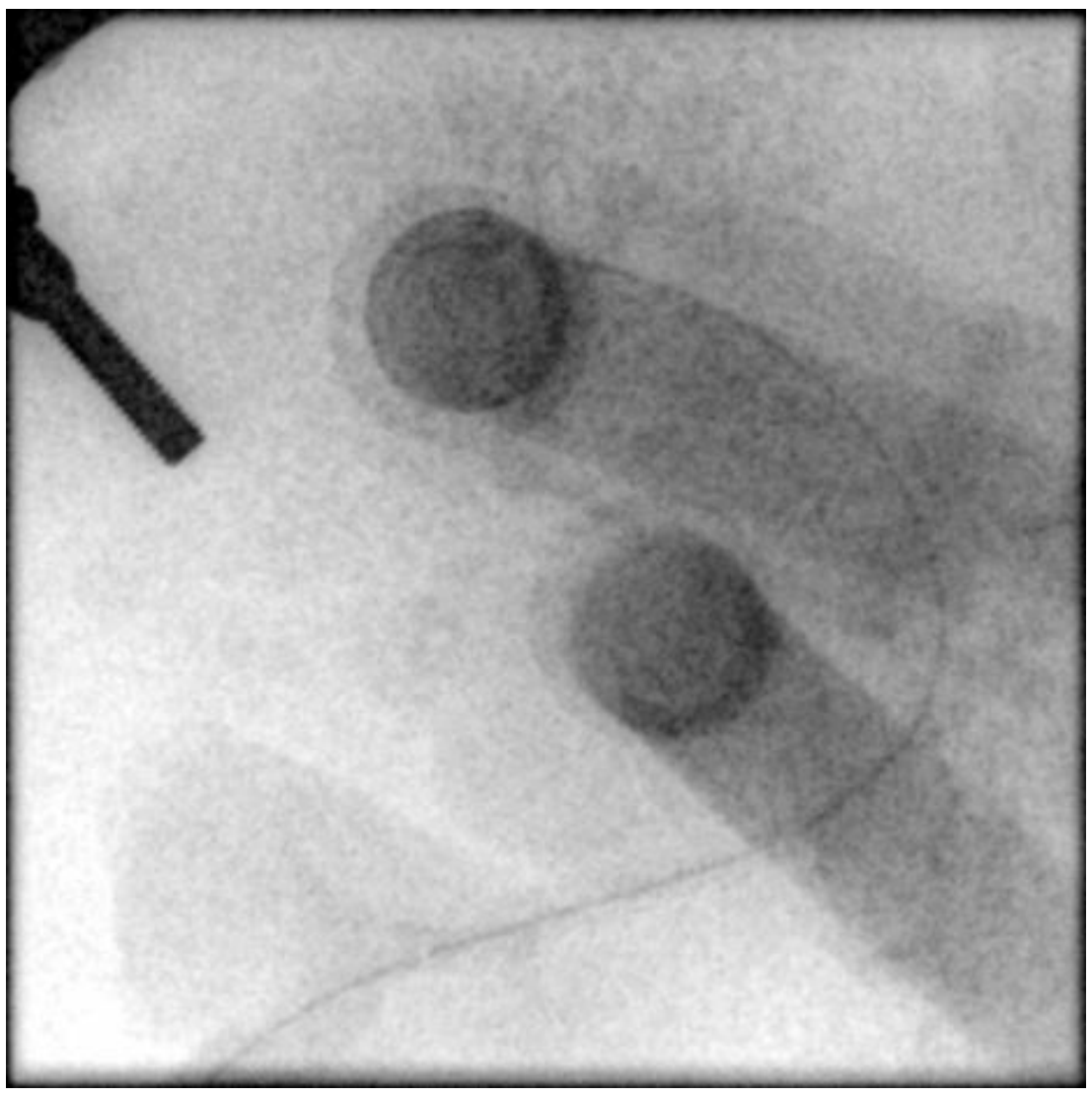

Figure S6: X-ray with the isolated catheter. Using the roadmap in Figure S4, the catheter tip location was identified at the top left of the upper circular structure.

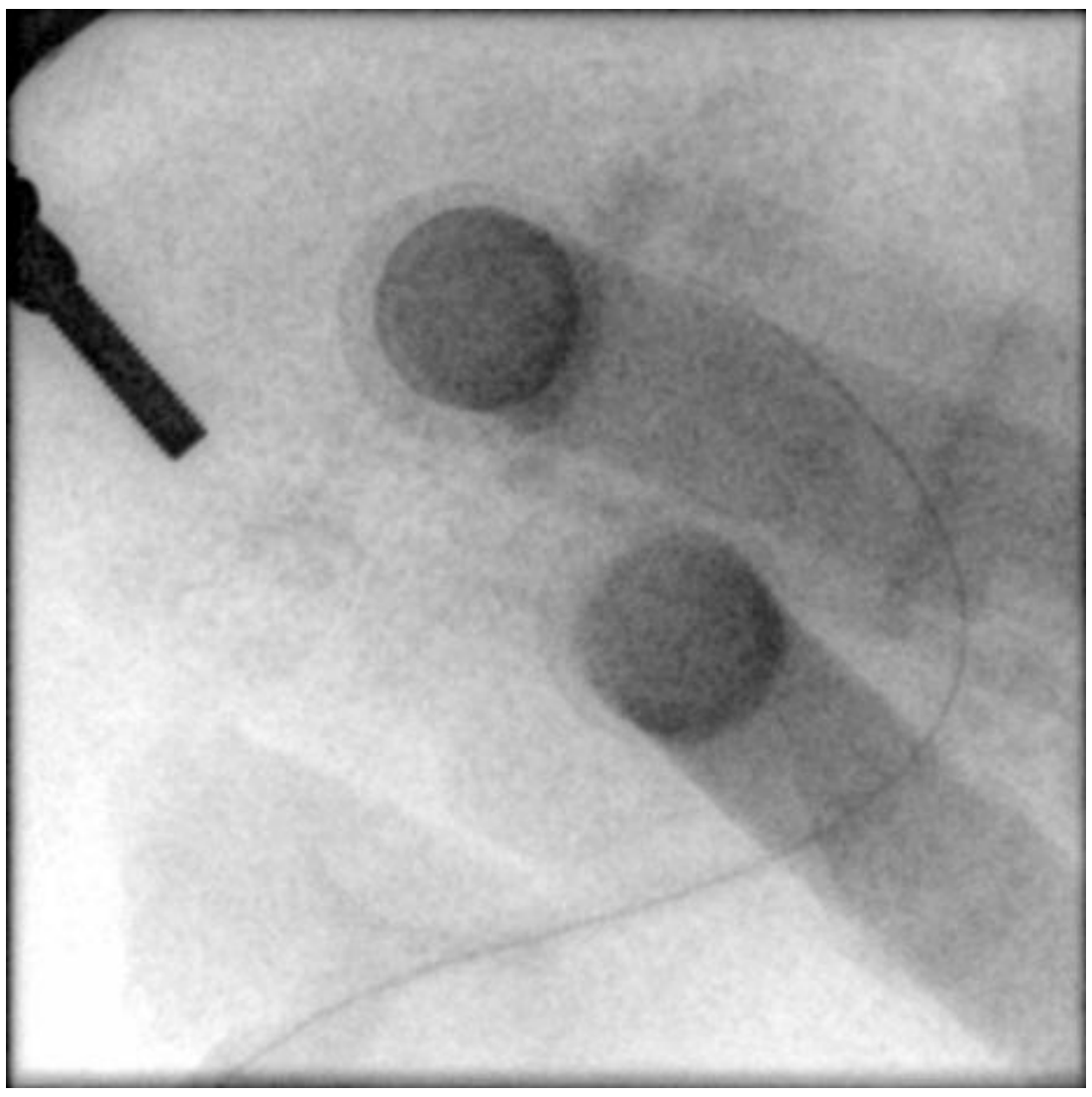

Figure S7: X-ray with the isolated catheter. Using the roadmap in Figure S4, the catheter tip location was identified on the right side of the upper circular structure.

Case 2

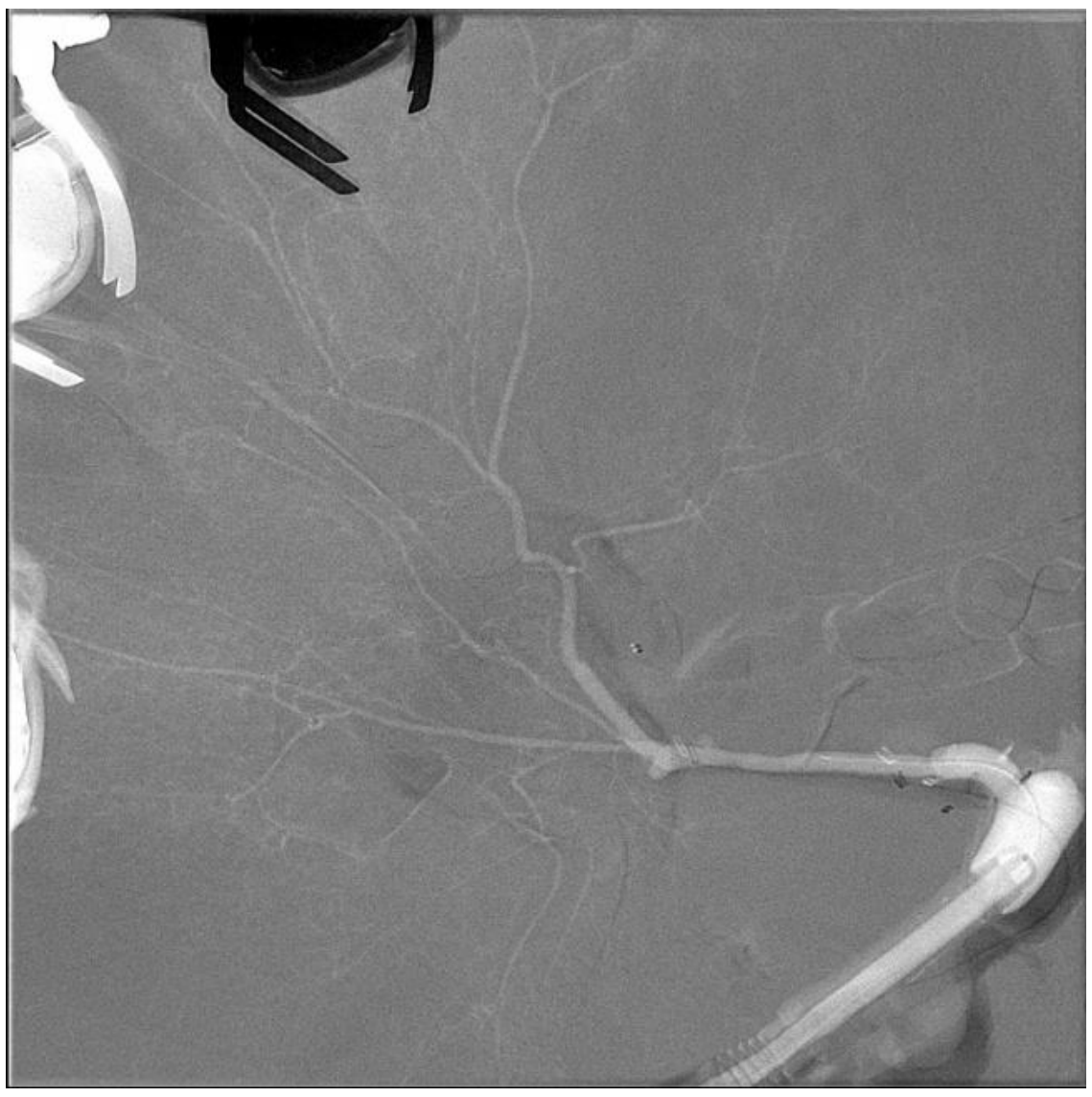

Figure S8: Fluoroscopy roadmap of catheter position P1 of Case 2, including the transducer positions in white (target probe on the top left, reference probe on the bottom left).

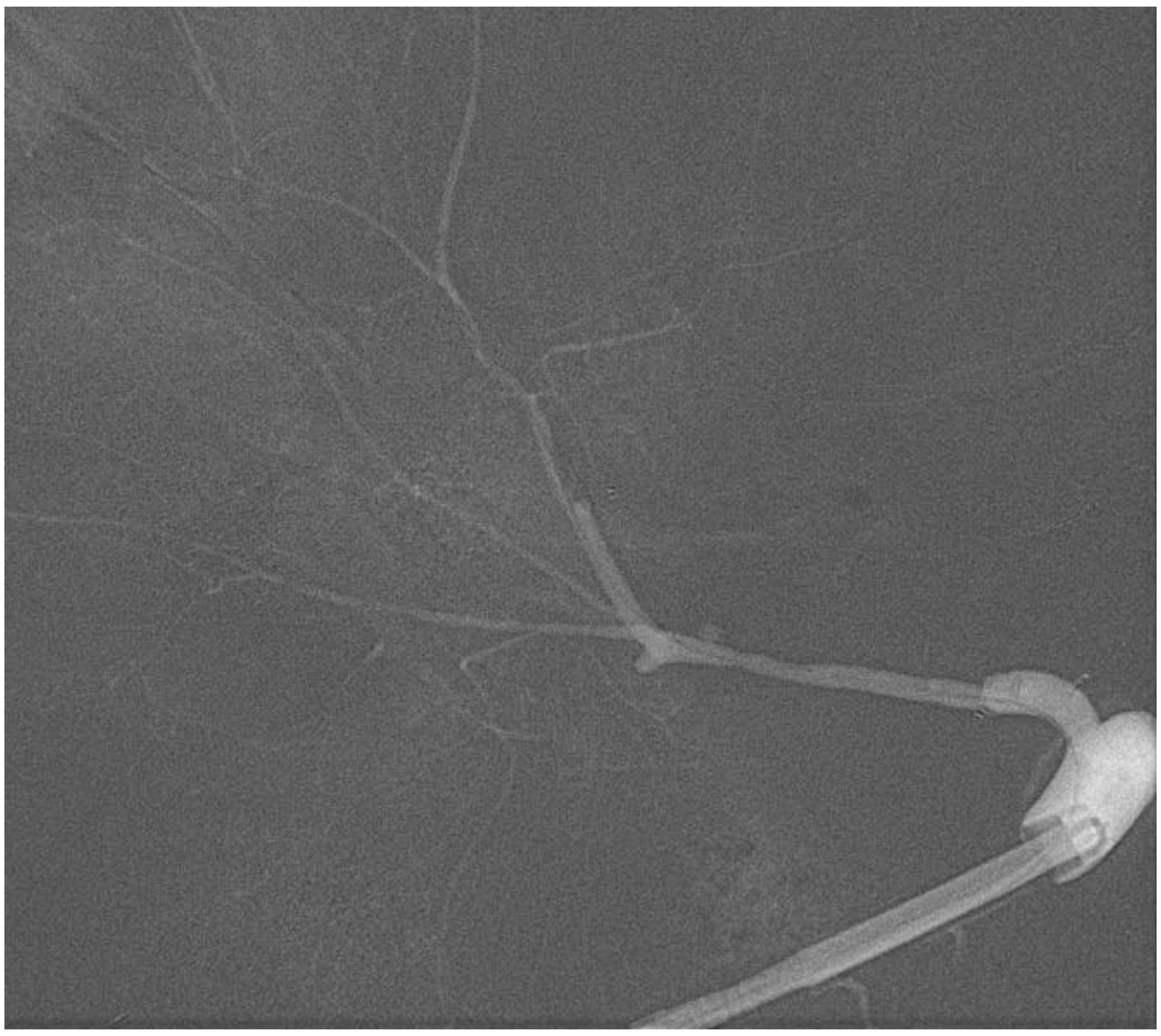

Figure S9: Fluoroscopy roadmap of catheter position P2 of Case 2.

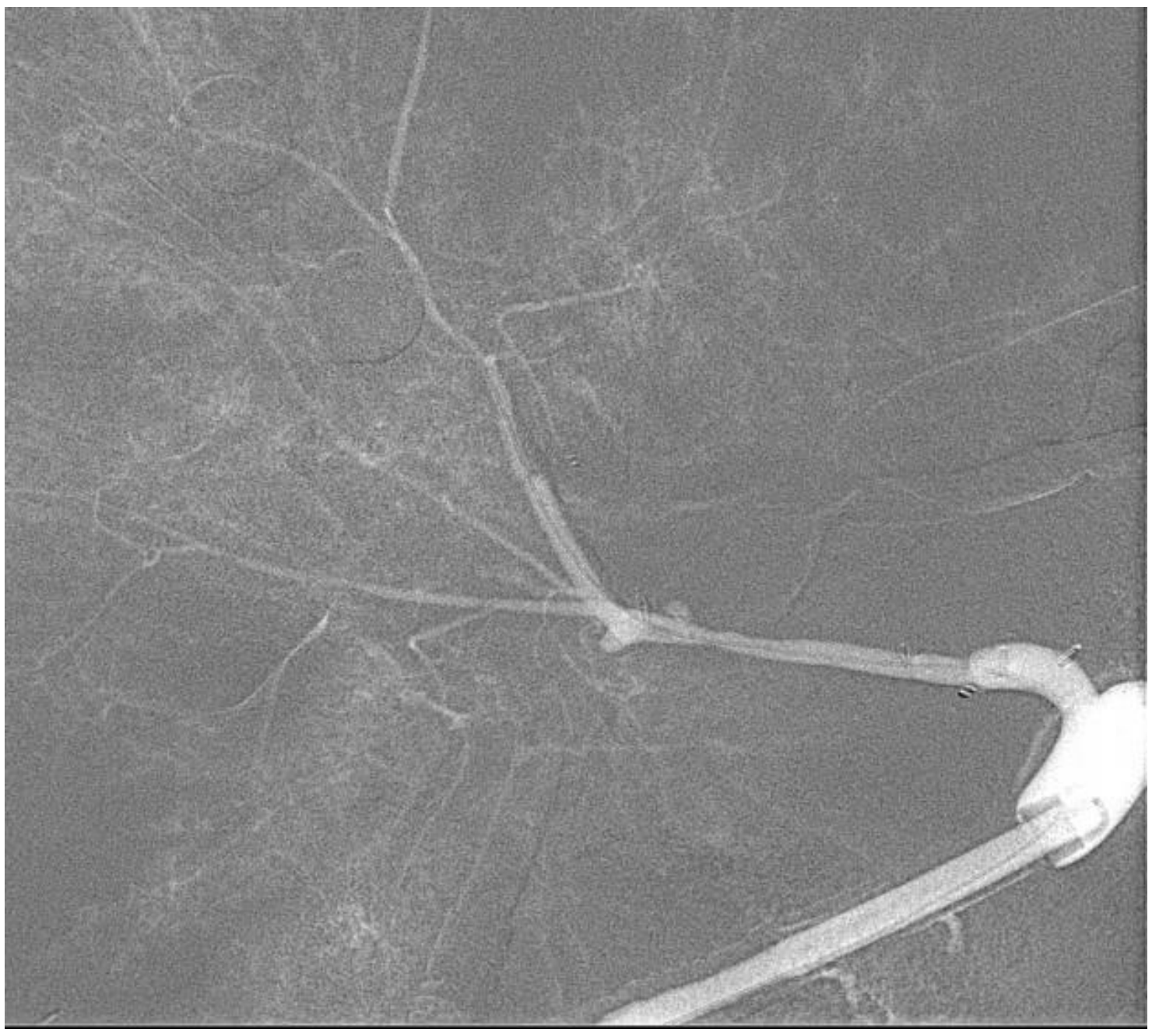

Figure S10: Fluoroscopy roadmap of catheter position P3 of Case 2.

Case 3

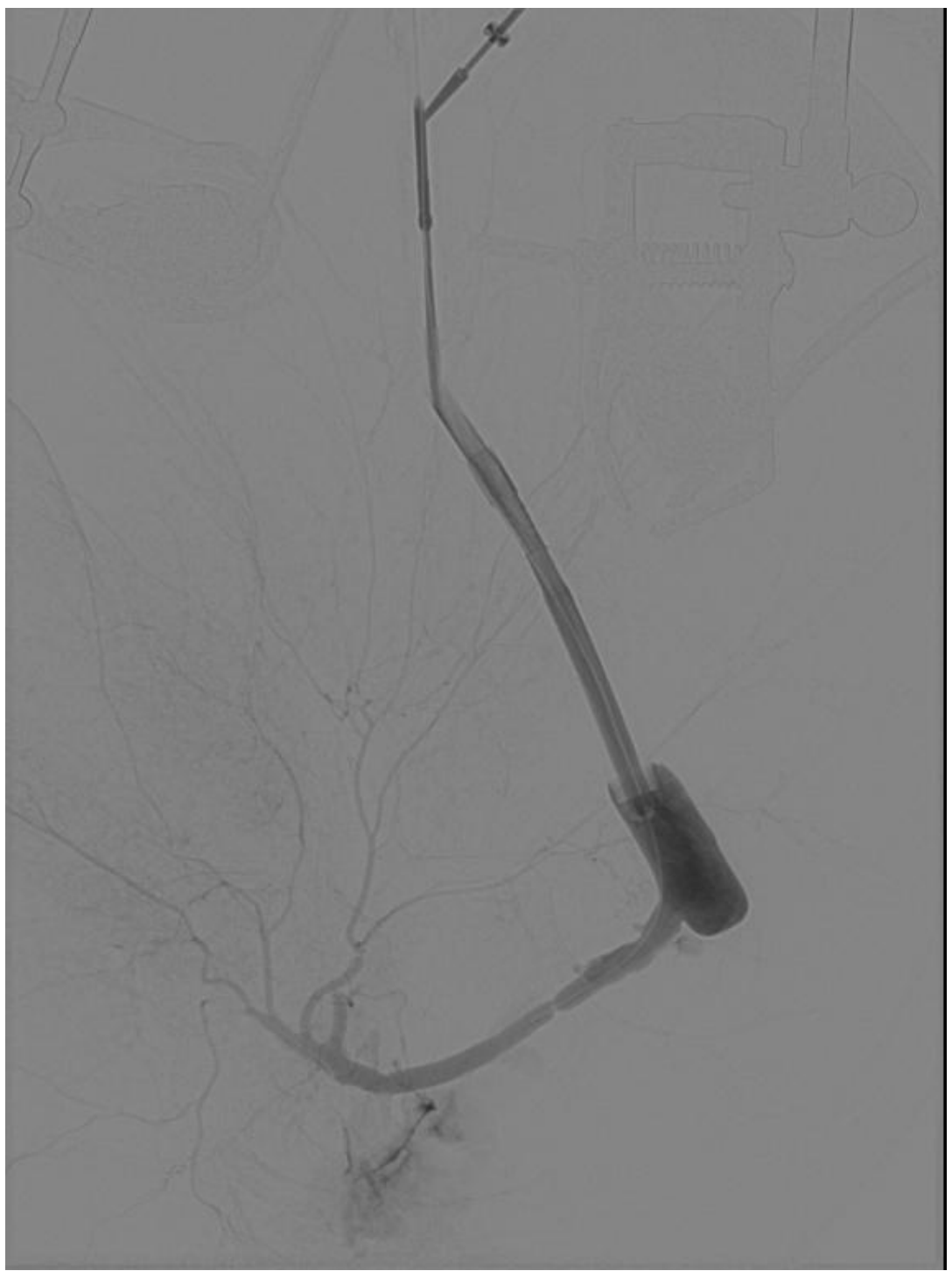

Figure S11: Digital subtraction angiography of catheter position P1 of Case 3.

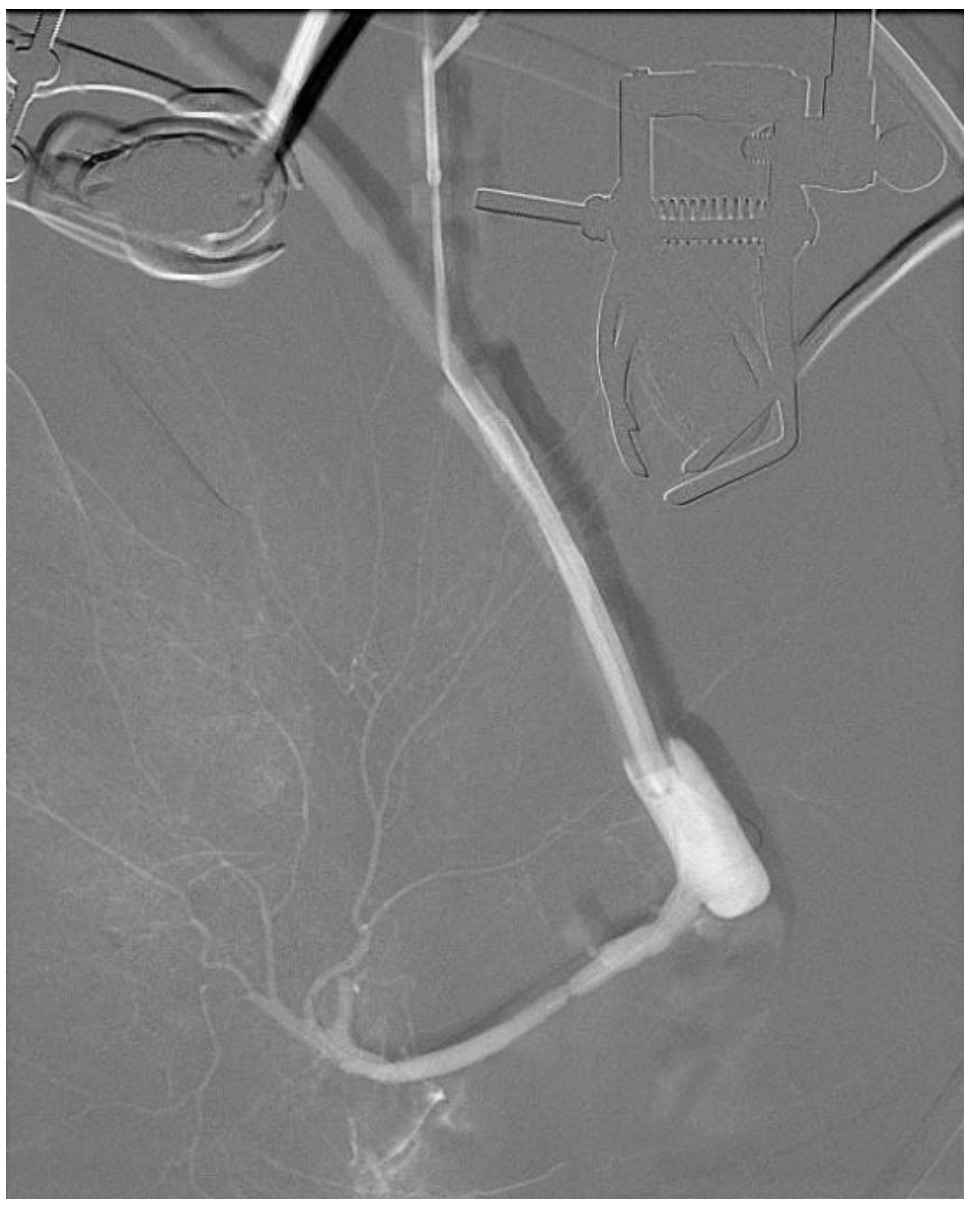

Figure S12: Fluoroscopy roadmap of catheter position P2 of Case 3, including the clamps and ultrasound transducers on the top, with the target probe on the left and the reference probe on the right.

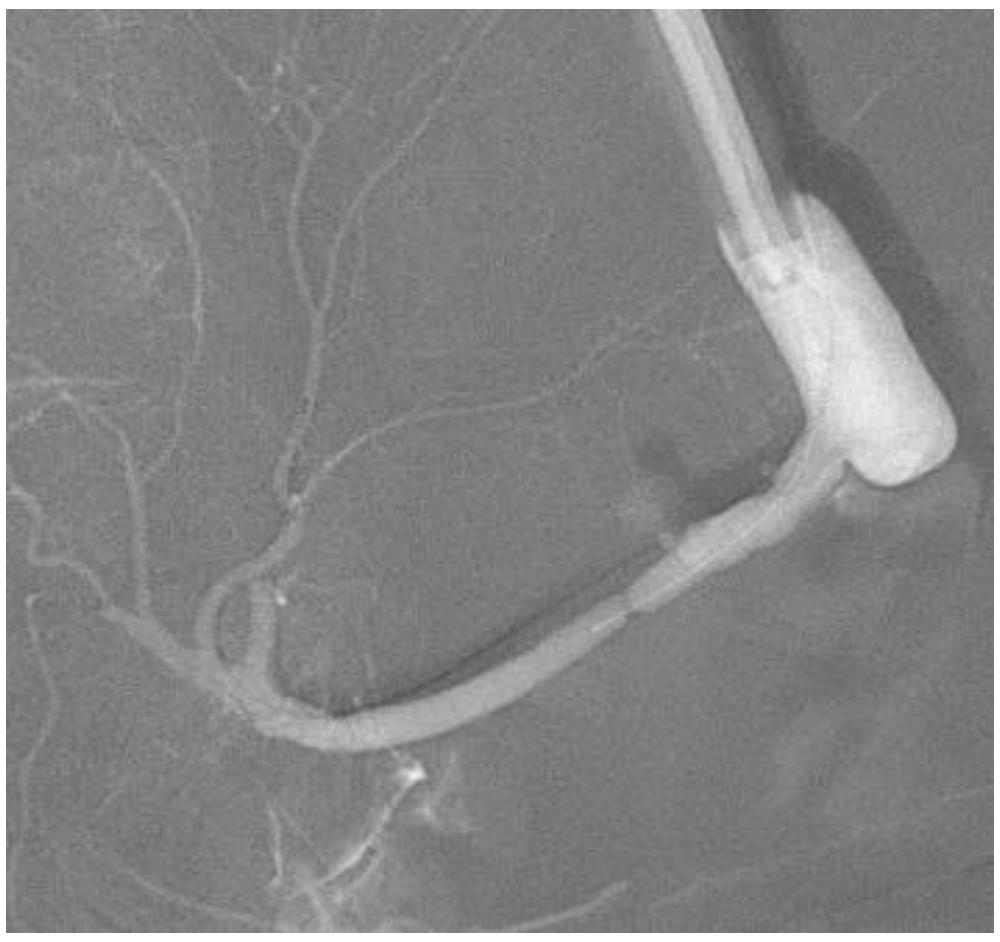

Figure S13: Fluoroscopy roadmap of catheter position P3 of Case 3.

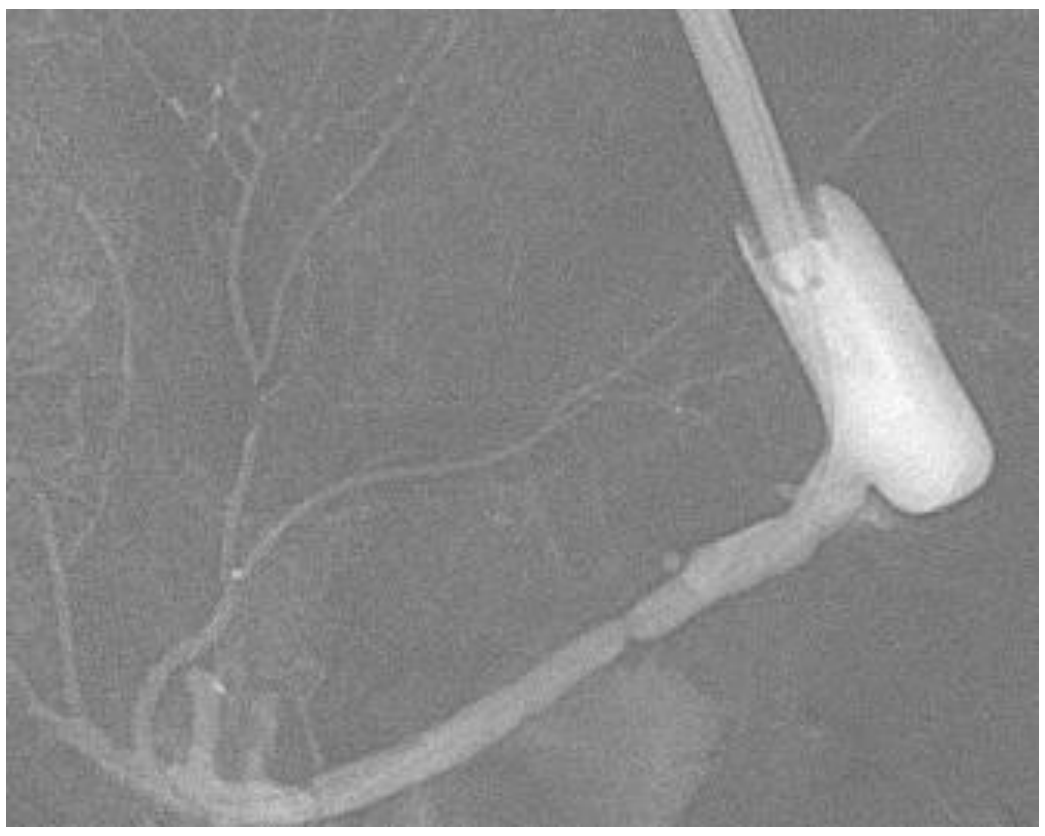

Figure S14: Fluoroscopy roadmap of catheter position P4 of Case 3.

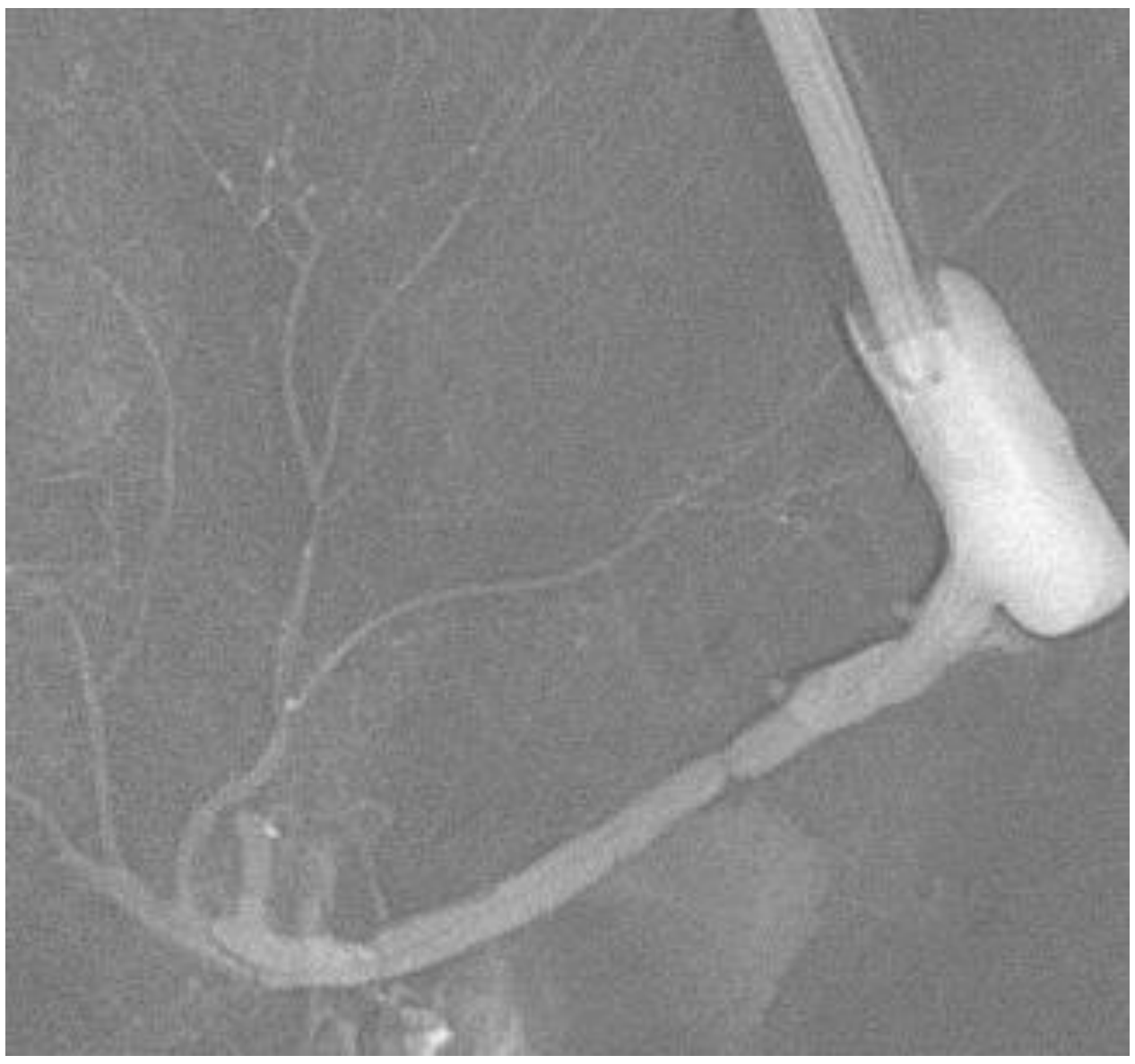

Figure S15: Fluoroscopy roadmap of catheter position P5 of Case 3.

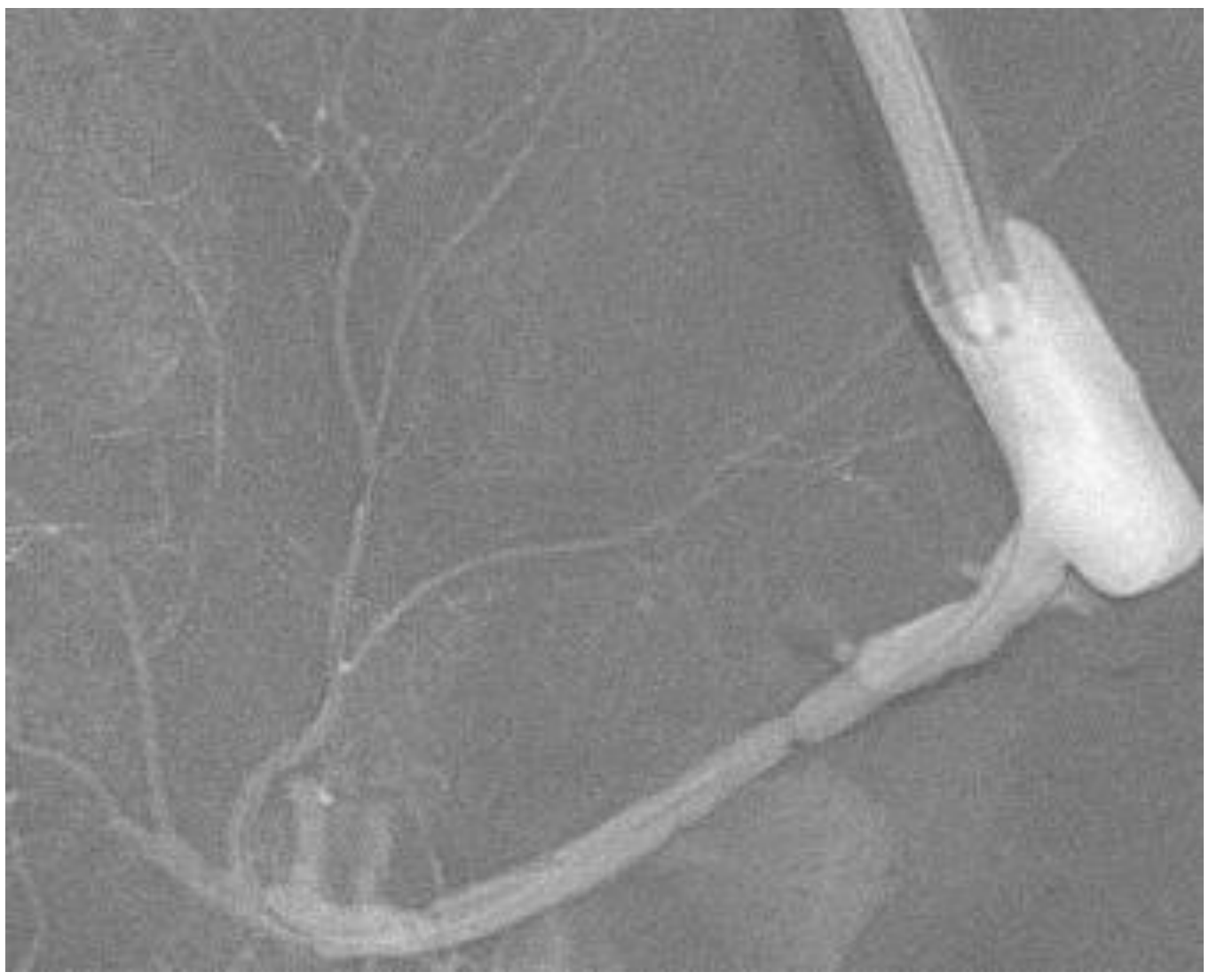

Figure S16: Fluoroscopy roadmap of catheter position P6 of Case 3.

3. Magnetic Resonance imaging liver condition

Case 1

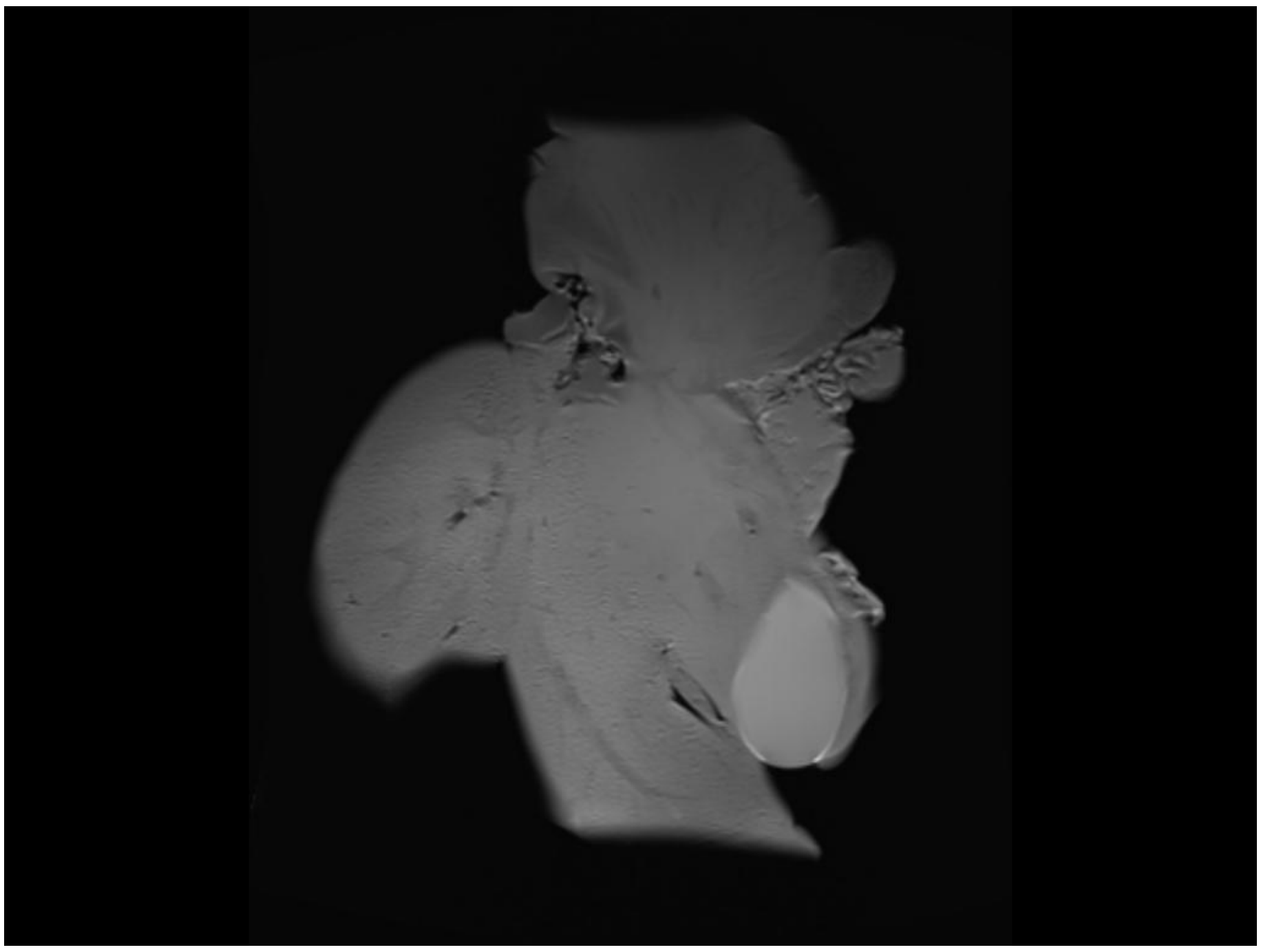

Figure S17: Anatomical T1-weighted spin-echo MRI scan of the liver (coronal plane) from case 1, acquired post-perfusion and after ^{165}Ho-microsphere administration. Image corresponds to slice 15.

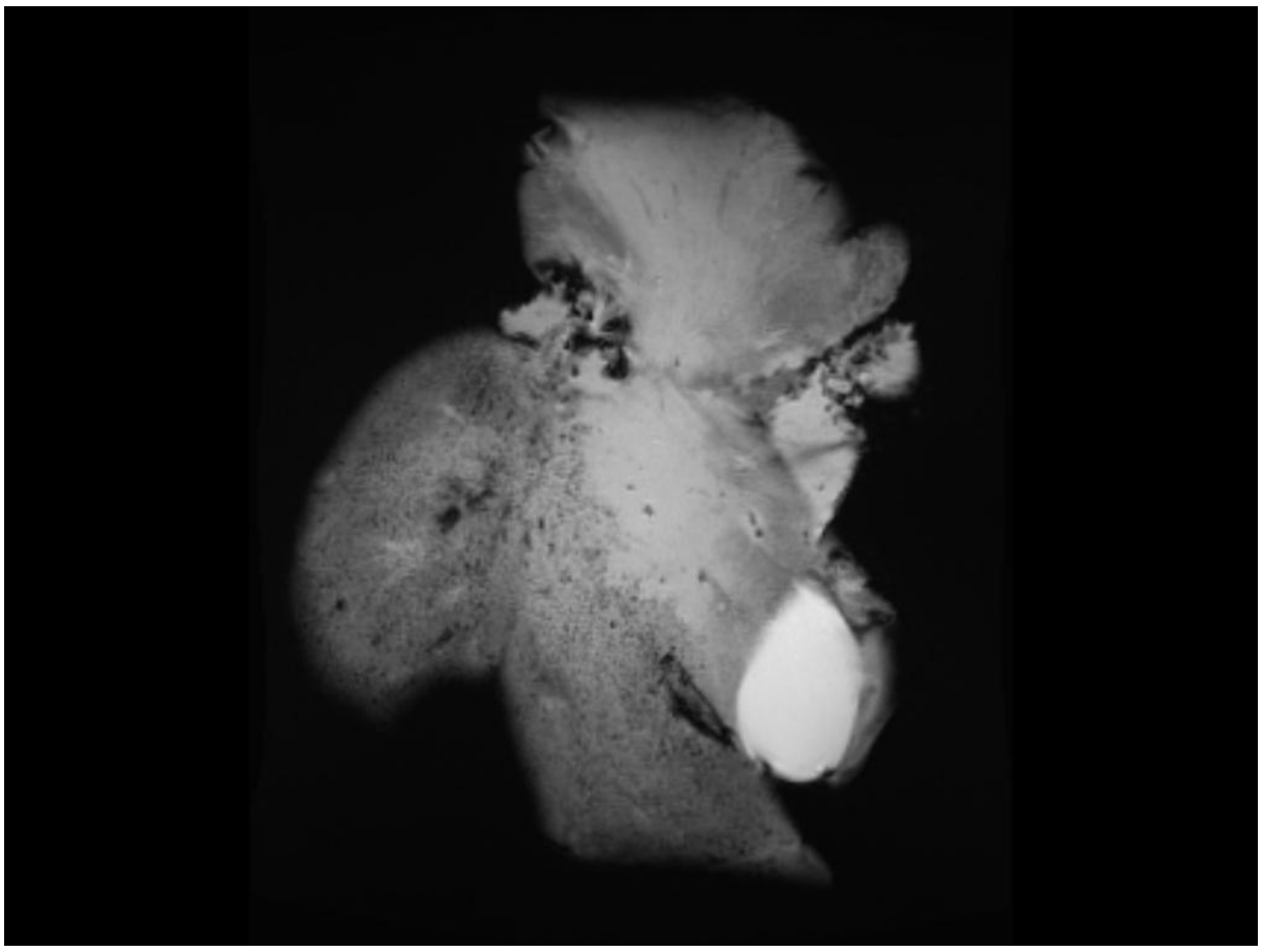

Figure S18: Holmium-sensitive T2-weighted multi-gradient MRI scan of the liver (coronal plane) from case 1, acquired post-perfusion and after ^{165}Ho-microsphere administration. Image corresponds to slice 15 at TE = 8.7 ms (echo number 5).*

Case 2

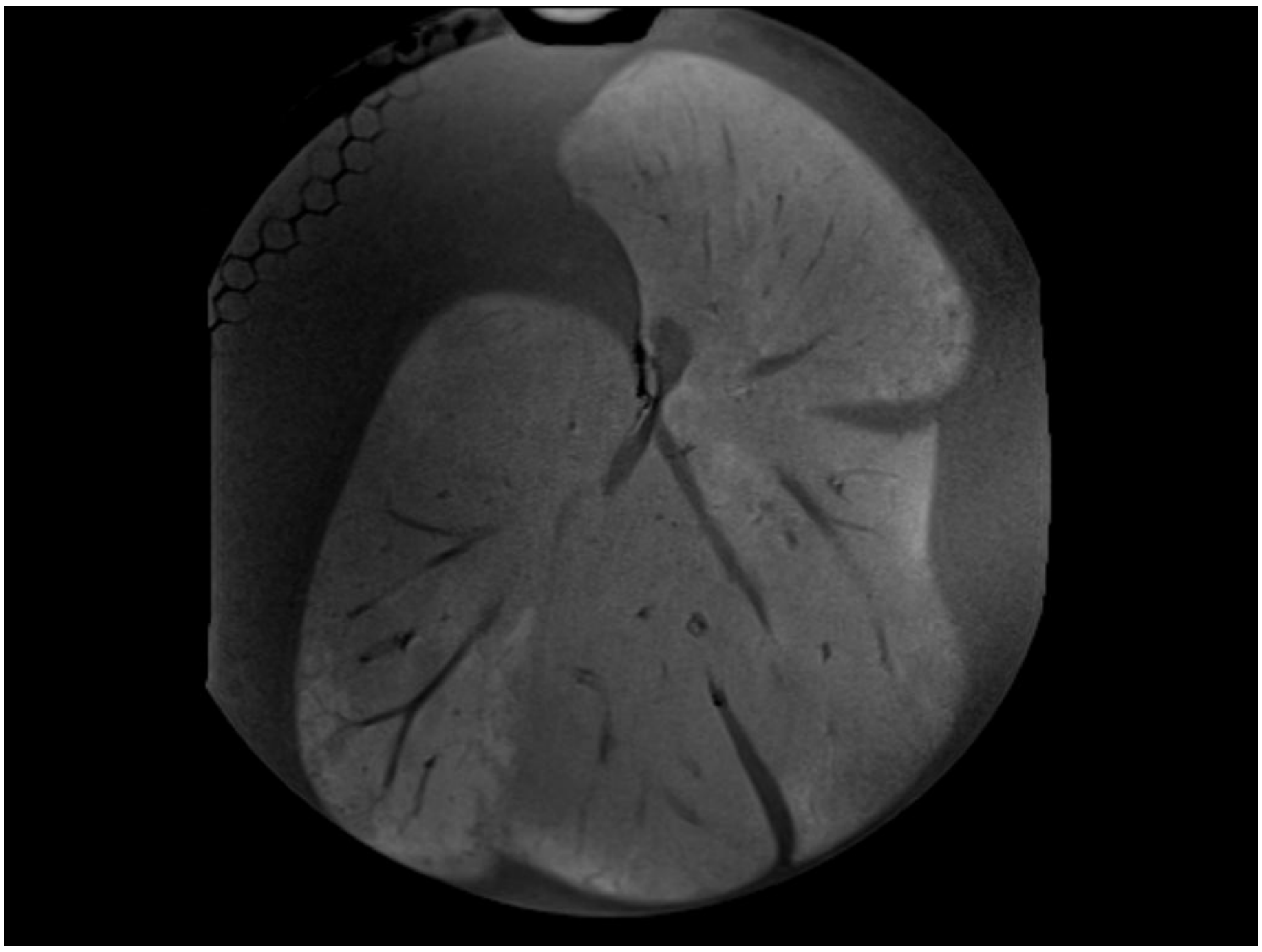

Figure S19: Anatomical T1-weighted VIBE-DIXON (water only) MRI scan of the liver (coronal plane) from case 2, acquired pre-perfusion and prior to ^{165}Ho-microsphere administration. Image corresponds to slice 67.

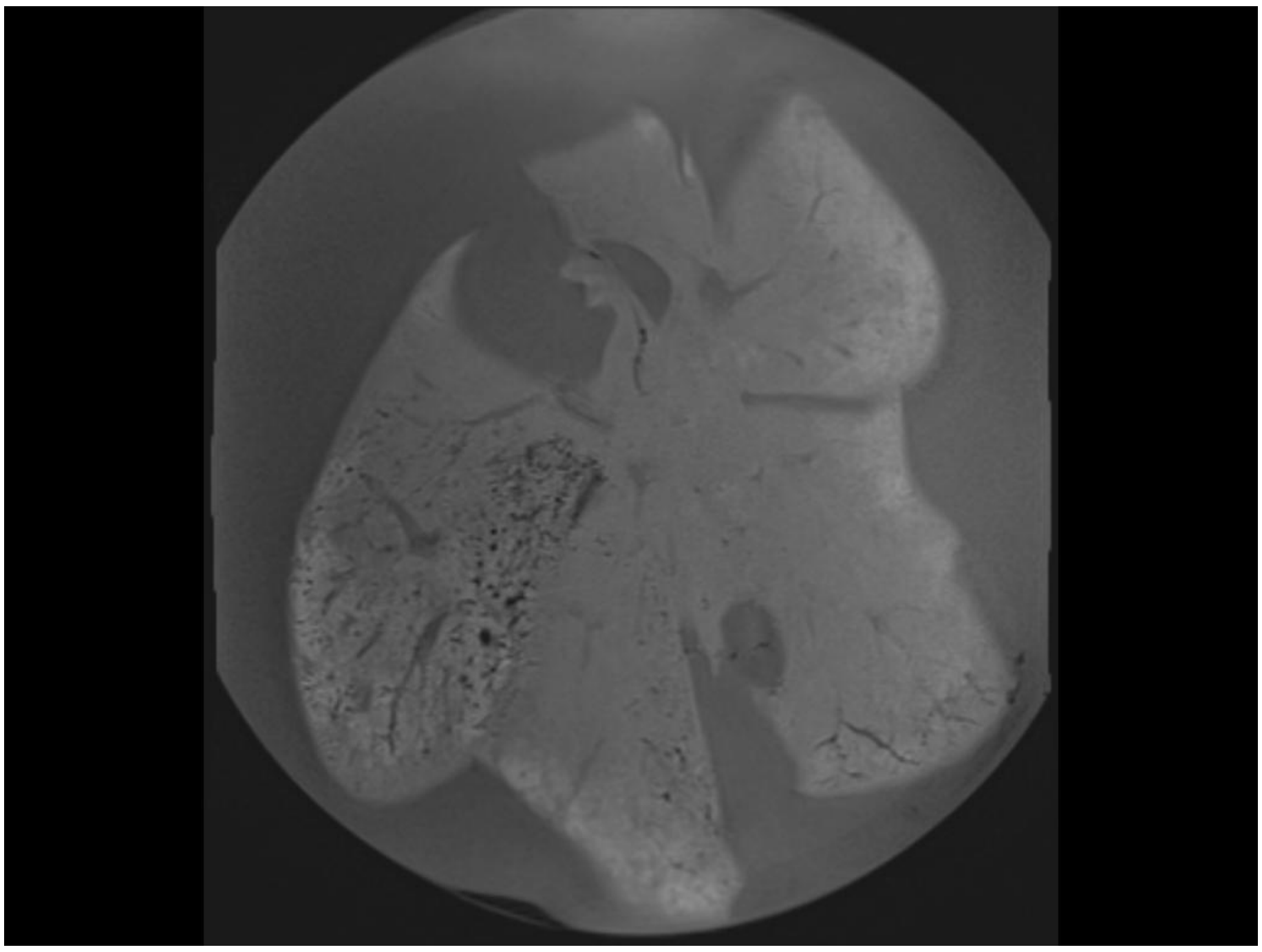

Figure S20: Anatomical T1-weighted VIBE-DIXON (water only) MRI scan of the liver (coronal plane) from case 2, acquired post-perfusion and after ^{165}Ho-microsphere administration. Image corresponds to slice 61.

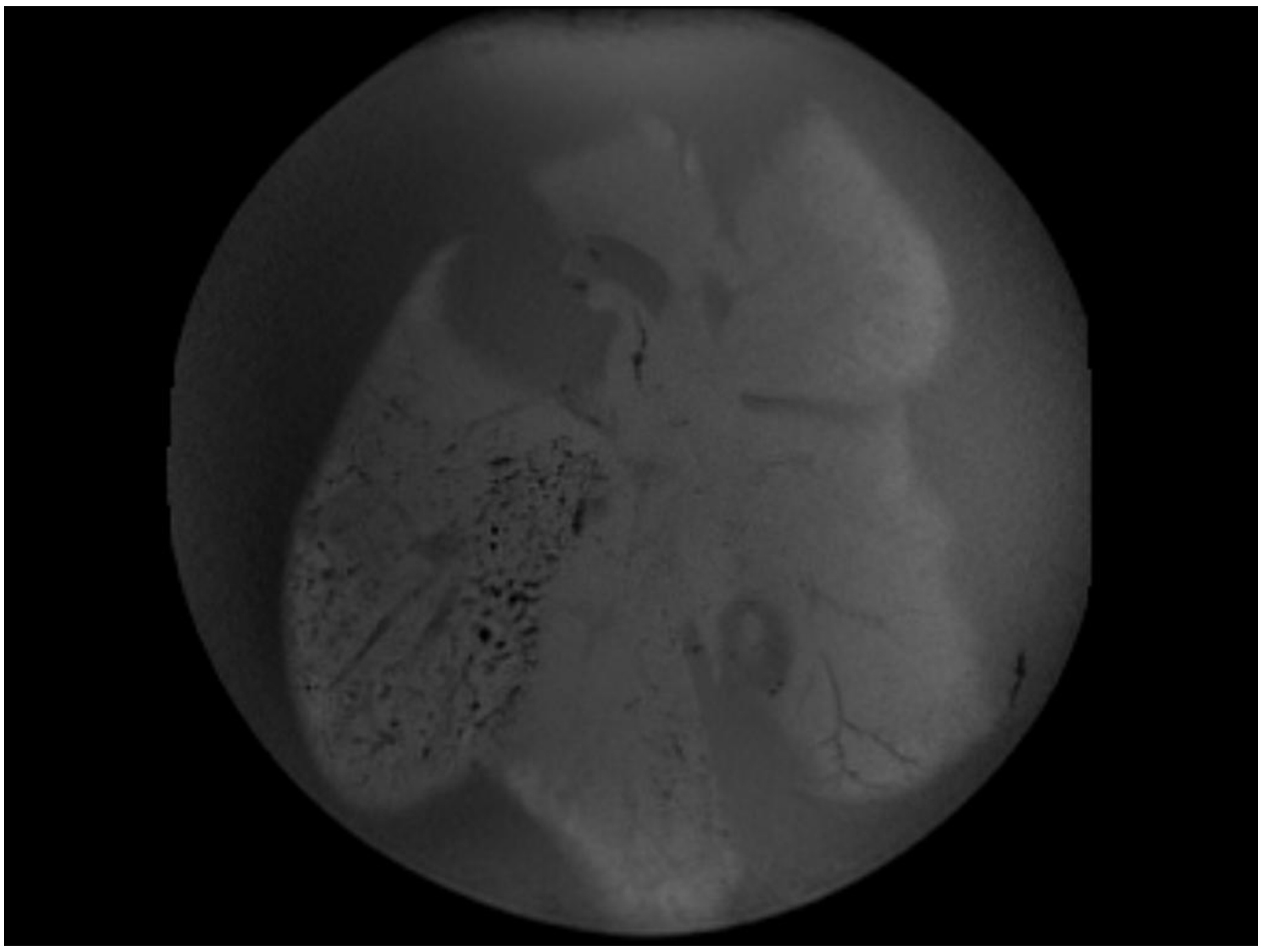

Figure S21: Holmium-sensitive T2-weighted multi-gradient MRI scan of the liver (coronal plane) from case 2, acquired post-perfusion and after ^{165}Ho-microsphere administration. Image corresponds to slice 16 at TE = 3.0 ms (echo number 2).*

Case 3

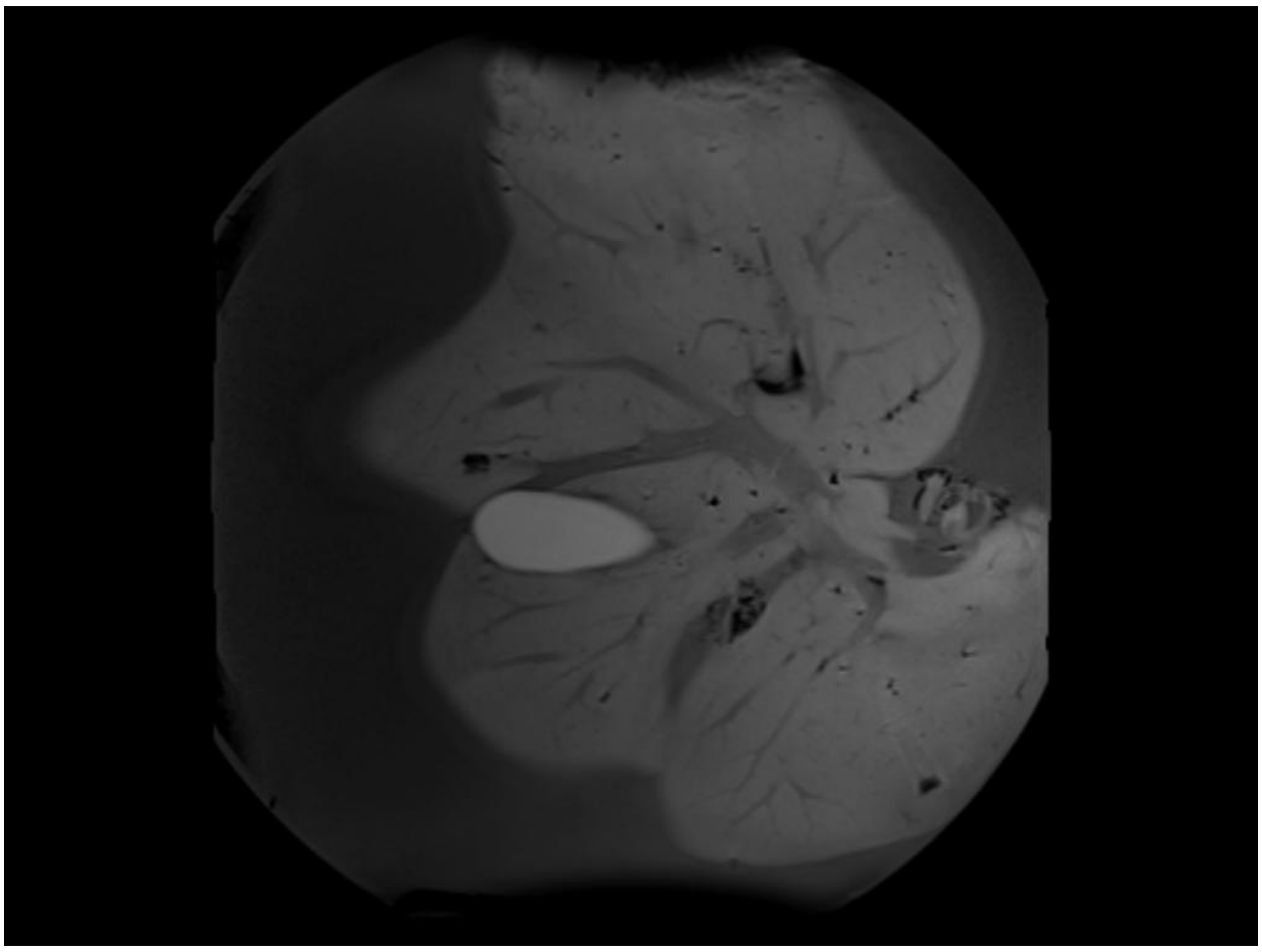

Figure S22: Anatomical T1-weighted VIBE-DIXON (water only) MRI scan of the liver (coronal plane) from case 3, acquired pre-perfusion and prior to ^{165}Ho-microsphere administration. Image corresponds to slice 34.

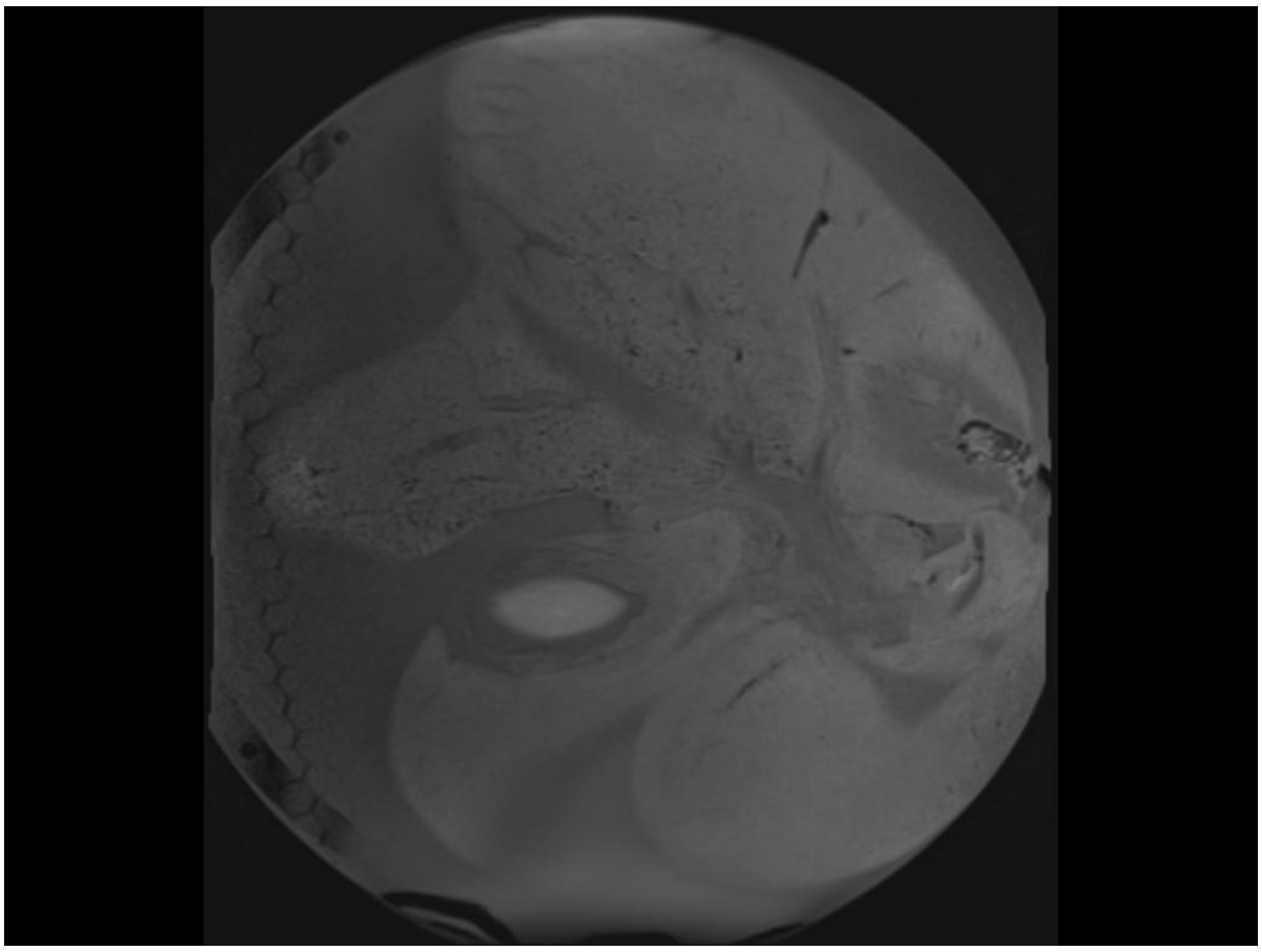

Figure S23: Anatomical T1-weighted VIBE-DIXON (water only) MRI scan of the liver (coronal plane) from case 3, acquired post-perfusion and after ^{165}Ho-microsphere administration. Image corresponds to slice 53.

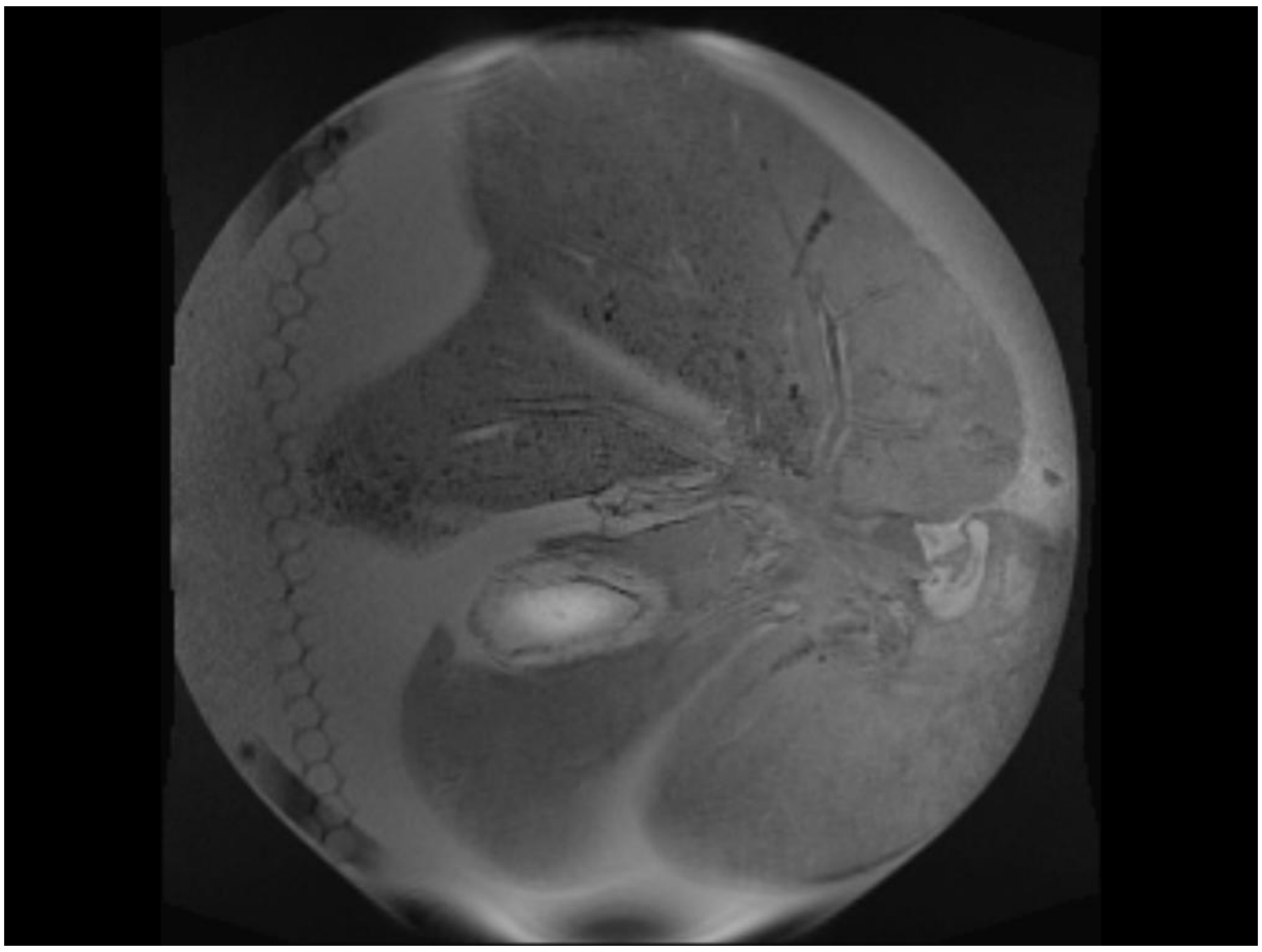

Figure S24: Holmium-sensitive T2-weighted multi-gradient MRI scan of the liver (coronal plane) from case 3, acquired post-perfusion and after ^{165}Ho-microsphere administration. Image corresponds to slice 15 at TE = 8.8 ms (echo number 5).*

4. Hemodynamic parameters

Case 2

Table S1: Hemodynamic parameters for reperfusion and subsequent normothermic perfusion of the porcine liver used for case 2

Time	Flow HA (ml/min)	Flow PV (ml/min)	P HA (mmHg)	P PV (mmHg)	Air flow (mL/min)
**0**	70-110	300	330	60	100
**15**	130-190	450	408	60	200
**30**	210-320	750	308	68	200
**45**	310-470	1150	240	70	300
**50**	360-540	1300	258	70	500
**end**	360-540	1250	240	64	500

Case 3

Table S2: Hemodynamic parameters for reperfusion and subsequent normothermic perfusion of the porcine liver used for case 3

Time	Flow HA (ml/min)	Flow PV (ml/min)	P HA (mmHg)	P PV (mmHg)	Air flow (mL/min)
**0**	80-120	320	316	62	100
**15**	180-220	650	340	70	200
**30**	220-270	920	308	82	200
**45**	360-540	1320	290	110	300
**50**	360-540	1320	258	90	400
**end**	400-550	1290	168	80	400

5. Blood gas parameters

Case 2

CG4+ analysis

Table S3: Blood gas parameter results from the CG4+ cartridge, measured at several time instances in the hepatic artery of the porcine liver used in case 2

Time	pH	PCO2 (kPa)	PO2 (kPa)	HCO3 (mmol/L)	BE (mmol/L)	sO2 (%)	TCO2 (mmol/L)	Lac (mmol/L)
**0**	7.14	6.27	8	16	-13	82	17	11.76
**50**	7.052	9.89	9.1	20.6	-10	83	23	11.82
**120**	7.051	7.12	7	14.8	-15	70	16	13.74
**end**	7.149	4.27	8.2	11.1	-17	84	12	14.87

CG8+ analysis

Table S4: Blood gas parameter results from the CG8+ cartridge, measured at several time instances in the hepatic artery of the porcine liver used in case 2

Time	pH	PCO2 (kPa)	PO2 (kPa)	HCO3 (mmol/L)	BE (mmol/L)	sO2 (%)	TCO2 (mmol/L)
**0**	7.057	8.15	5.2	17.2	-14	51	19
**50**	7.056	10.18	8.5	21.4	-10	80	24
**120**	7.067	6.8	6.7	14.7	-15	69	16
**end**	7.174	4.07	8.5	11.2	-16	86	12
**Time**	**Na (mmol/L)**	**K (mmol/L)**	**iCa (mmol/L)**	**Glu (mmol/L)**	**Hct (%PCV)**	**Hb* (mmol/L)**	
**0**	149	8.1	1.4	21.1	43	9.1	
**50**	140	> 9	0.98	33.2	35	7.4	
**120**	139	> 9	0.95	> 38.9	31	6.5	
**end**	140	> 9	0.89	> 38.9	31	6.5	

Case 3

CG4+ analysis

Table S5: Blood gas parameter results from the CG4+ cartridge, measured at several time instances in the hepatic artery of the porcine liver used in case 3

Time	pH	PCO2 (kPa)	PO2 (kPa)	HCO3 (mmol/L)	BE (mmol/L)	sO2 (%)	TCO2 (mmol/L)	Lac (mmol/L)
**0**	6.854	> 17.33	2.6	-	-	-	-	12.81
**50**	7.072	8.76	5	19.1	-11	49	21	12.86
**end**	7.12	9.33	4.2	22.7	-8	41	25	13.71

CG8+ analysis

Table S6: Blood gas parameter results from the CG8+ cartridge, measured at several time instances in the hepatic artery of the porcine liver used in case 3

Time	pH	PCO2 (kPa)	PO2 (kPa)	HCO3 (mmol/L)	BE (mmol/L)	sO2 (%)	TCO2 (mmol/L)
**0**	6.85	> 17.33	2.6	-	-	-	-
**50**	7.065	8.95	5.02	19.2	-12	51	21
**end**	7.126	9.6	4.2	23.7	-7	41	26
**Time**	**Na (mmol/L)**	**K (mmol/L)**	**iCa (mmol/L)**	**Glu (mmol/L)**	**Hct (%PCV)**	**Hb* (mmol/L)**	
**0**	140	> 9	1.3	16.8	45	9.5	
**50**	137	> 9	0.88	11.1	39	8.2	
**end**	144	> 9	0.74	8.5	40	8.4	

6. Time-intensity curve metrics

Case 1

Table S7: Time-intensity curve metrics for case 1 (single-transducer). Mean values are given relative to position P4, the position used for the ^{165}Ho-microsphere injection. Standard deviations (STD) are given in 1 σ. From the mean values, the T_P/T_1 ratio is calculated to quantify the relative improvement with respect to the first injection location.

Pos	Mean (%)	STD (%)	Ratio T_P/T_1
1	34.7	11.2	-
2	86.3	11.0	2.5
3	4.8	3.3	0.14
**4**	100.0	9.7	2.9

Case 2

Table S8: Time-intensity curve metrics for case 2 (dual transducer). Mean values are given relative to position P2, the position used for the ^{165}Ho-microsphere injection. Standard deviations (STD) are given in 1 σ. Two ratios are computed from the mean values: T_P/T_1 and R_P/R_1, quantifying the relative improvement with respect to the first injection location, and T_P/R_P, indicating the target-to-reference signal ratio.

Pos	Transducer	Mean (%)	STD (%)	T_P/T_1 and R_P/R_1	T_P/R_P
1	***Tar***	65.8	3.2	-	1.2
	Ref	54.1	3.1	-	
2	***Tar***	**100.0**	3.3	1.5	48
	Ref	2.1	1.5	0.04	
3	***Tar***	80.9	11.3	1.2	58
	Ref	1.4	1.5	0.03	

Case 3

Table S9: Time-intensity curve metrics for case 2 (dual transducer). Mean values are given relative to position P6, the position used for the ^{165}Ho-microsphere injection. Standard deviations (STD) are given in 1 σ. Two ratios are computed from the mean values: T_P/T_1 and R_P/R_1, quantifying the relative improvement with respect to the first position, and T_P/R_P, indicating the target-to-reference signal ratio.

Pos	Transducer	Mean (%)	STD (%)	T_P/T_1 and R_P/R_1	Ratio T_P/R_P
1	**Tar**	33.8	2.6	-	0.69
	**Ref**	49.0	4.6	-	
2	**Tar**	72.1	1.7	2.1	0.79
	**Ref**	90.8	1.1	1.9	
3	**Tar**	61.0	18.2	1.8	0.53
	**Ref**	114.3	8.6	2.3	
4	**Tar**	0.3	0.3	0.009	0.0025
	**Ref**	118.9	8.7	2.4	
5	**Tar**	90.1	12.8	2.7	0.92
	**Ref**	98.3	11.4	2.0	
6	**Tar**	**100.0**	3.8	3.0	3.4
	**Ref**	29.2	15.2	0.6	

7. In house DCE-US algorithm comparison to Vuebox

For the comparison of the in house DCE-US algorithm with Vuebox, a preliminary dataset was analyzed by both methods. The dataset consisted of 25 DICOM clips of DCE-US measurements recorded at three ultrasound transducer positions (Figure S25).

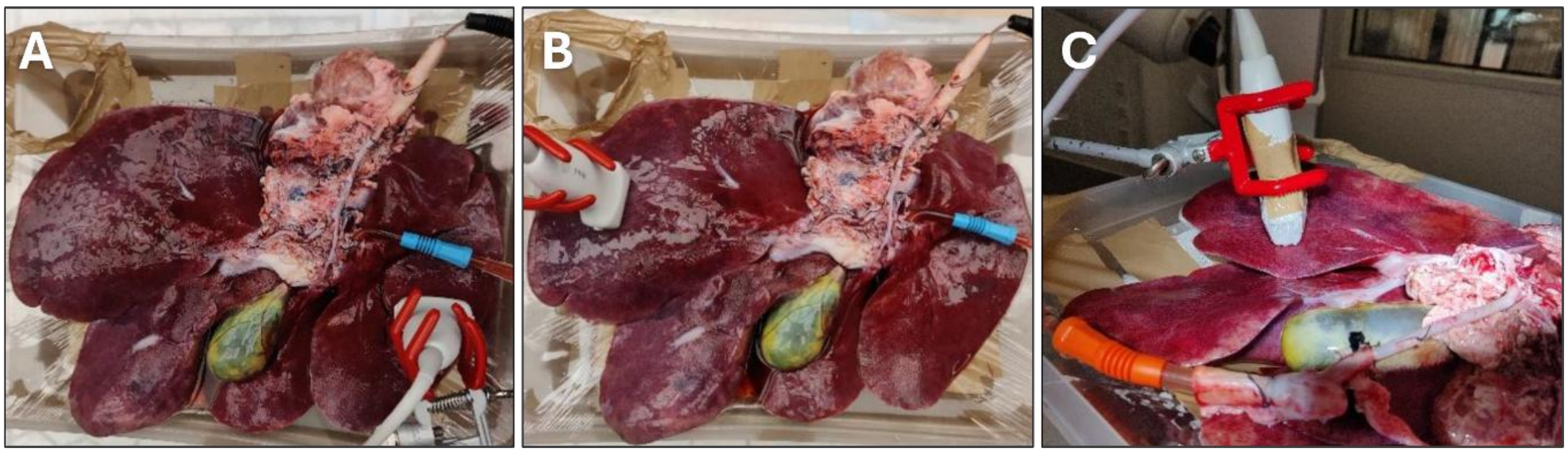

Figure S25: The three analyzed ultrasound transducer positions for comparison of the in house DCE-US algorithm and Vuebox.

For each measurement, the catheter position was slightly adjusted to acquire different peak intensities (PI). The injection protocol followed the method described in the main text. For lobe 1, the catheter was positioned at five different locations, and three repeated measurements were acquired at each location. For lobes 2 and 3, the catheter was placed at two different positions, with three repeated measurements per location. For two measurements, comparison of the time–intensity curves was not possible because the signal characteristics did not allow Vuebox to generate a reliable curve.

The resulting PIs from the in-house algorithm and Vuebox are shown in Table 1. The in-house algorithm calculates the time–intensity curve in arbitrary units, whereas Vuebox reports PI values in decibels (dB). A translation factor between the two scales was obtained using the MATLAB Curve Fitter toolbox, which minimized the sum of squared errors (SSE):

$$PI_{vuebox} = PI_{in\ house} * 0.12475 + 24.5051.$$

After conversion to decibels, comparison with the Vuebox results showed a mean difference of 0.59 dB and a maximum difference of 1.29 dB. The individual values and the resulting linear regression are presented in Figure S26.

Table S10: An overview of the measurements (mes) and the resulting peak intensities (PI) on the different lobes (lobe 1 – 3). For the in house algorithm, the PI values converted to decibels (dB) using the linear regression with Vuebox are shown alongside the Vuebox results. The last column reports the difference between both methods (in dB), with a mean absolute difference of 0.59 dB.

Measurement	**PI – In house (-)**	**PI - VueBox (dB)**	**PI – In house (dB)**	**Difference (dB)**
Lobe 1, mes 1	98.65	35.52	36.81	1.29
Lobe 1, mes 2	92.34	35.89	36.02	0.13
Lobe 1, mes 3	87.91	34.62	35.47	0.85
Lobe 1, mes 4	94.94	35.29	36.35	1.059
Lobe 1, mes 5	95.32	35.31	36.40	1.09
Lobe 1, mes 6	96.67	36.89	36.56	-0.33
Lobe 1, mes 7	93.87	35.92	36.21	0.29
Lobe 1, mes 8	78.78	35.18	34.33	-0.85
Lobe 1, mes 9	86.48	34.74	35.29	0.55
Lobe 1, mes 10	62.20	31.19	32.26	1.07
Lobe 1, mes 11	63.68	31.77	32.45	0.68
Lobe 1, mes 12	51.98	30.99	30.99	0.00
Lobe 1, mes 13	56.25	30.33	31.52	1.19
Lobe 1, mes 14	39.66	28.22	29.45	1.23
Lobe 2, mes 1	52.43	31.12	31.05	-0.07
Lobe 2, mes 2	51.32	30.67	30.91	0.24
Lobe 2, mes 3	51.73	30.85	30.96	0.11
Lobe 2, mes 4	52.06	30.28	31.00	0.72
Lobe 2, mes 5	53.53	30.81	31.18	0.37
Lobe 2, mes 6	48.81	30.27	30.59	0.32
Lobe 3, mes 1	54.43	32.18	31.29	-0.89
Lobe 3, mes 2	46.13	30.83	30.26	-0.57
Lobe 3, mes 3	54.61	31.68	31.32	-0.36
Lobe 3, mes 4	44.34	29.68	30.04	0.36
Lobe 3, mes 5	39.88	29.22	29.48	0.26

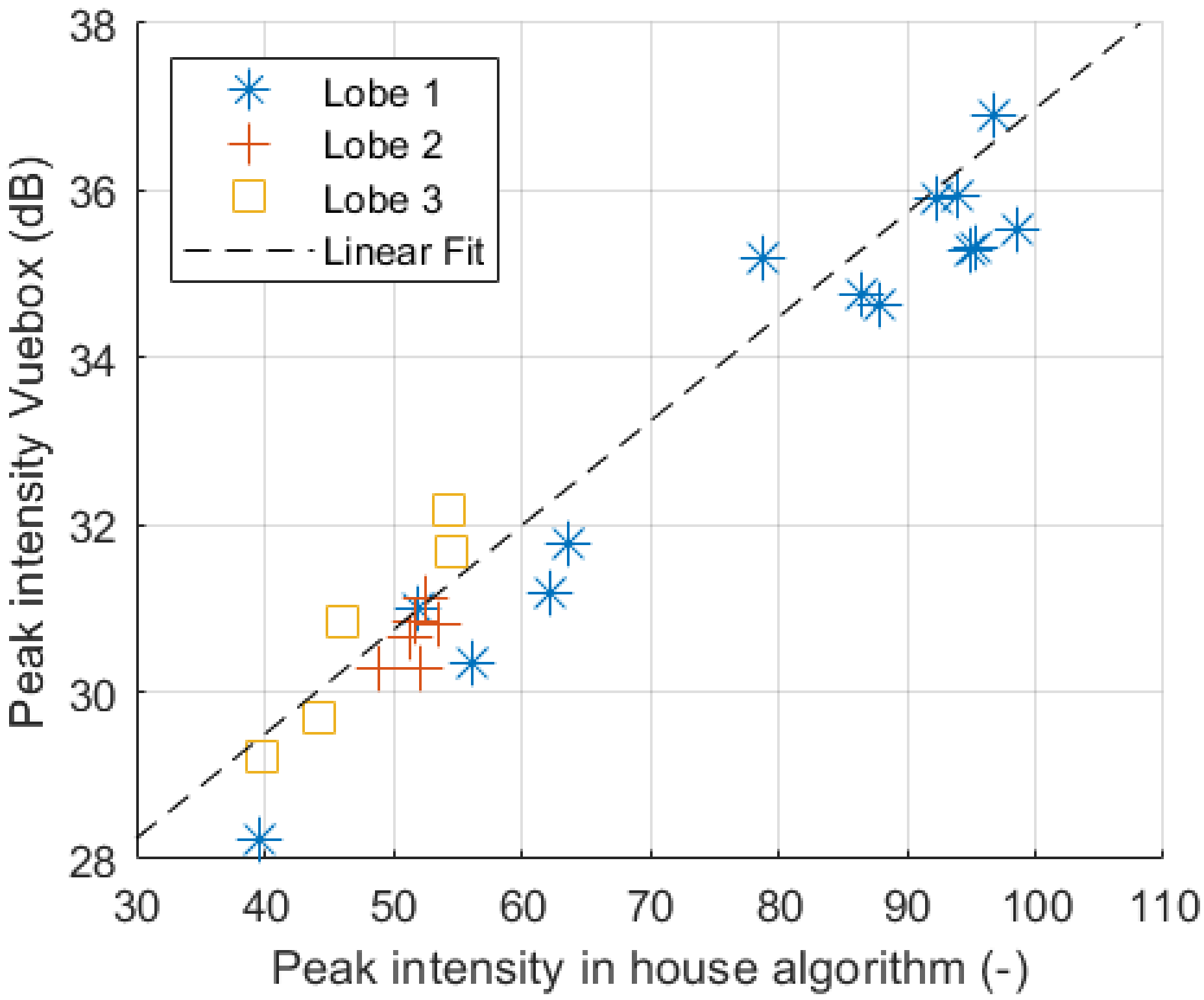

Figure S26: Linear relationship between the Vuebox peak-intensity results and the in house algorithm peak intensities converted to decibels (dB), shown for all measurements across the different lobes.
