# Supplementary material for: Dynamic contrast-enhanced ultrasound predicts microsphere biodistribution in real-time during transarterial radioembolization
Source: Commun Med (Lond). 2026 Jun 30;6:546. doi: 10.1038/s43856-026-01760-x (PMC13683390; doi:10.1038/s43856-026-01760-x)
Supplement: Supplementary file 3 [file 43856_2026_1760_MOESM3_ESM.pdf]

Description of Additional Supplementary Files

File name: Supplementary Data 1

Description: Source Data for all time-intensity curves
